# PSMD11 promotes hepatocellular carcinoma progression by reprogramming lipid metabolism via SREBP1/2 and PPAR-γ

**DOI:** 10.1038/s41419-026-09175-1

**Published:** 2026-08-18

**Authors:** Zhihong Huang, Canxue Zhang, Chi Zhang, Yuqi Liu, Chao Ge, Zhujun Wei, Li Zhang, Yue Yang, Wei Tian, Hua Tian

**Affiliations:** 1 State Key Laboratory of Systems Medicine for Cancer, Shanghai Cancer Institute, Renji Hospital, Shanghai Jiao Tong University School of Medicine, Shanghai, China; 2 Department of Pathology, The Affiliated Hospital of Youjiang Medical University for Nationalities, Baise, China; 3 The Key Laboratory of Molecular Pathology (Hepatobiliary Diseases) of Guangxi, Baise, China

**Keywords:** Liver cancer, Lipid signalling

## Abstract

Dysregulation of the ubiquitin-proteasome system (UPS) plays a pivotal role in tumor progression. PSMD11, a non-ATPase regulatory subunit of the 26S proteasome, has been implicated as an oncogenic factor in several cancer types; however, its precise biological function and molecular mechanisms in hepatocellular carcinoma (HCC) remain incompletely understood. In this study, we demonstrated that PSMD11 was markedly upregulated in HCC tissues and correlated with poor patient prognosis. Functionally, PSMD11 overexpression promoted HCC cell proliferation, migration, and invasion, whereas its knockdown exerted opposite effects. Mechanistically, we reveal that PSMD11 drives HCC progression by reprogramming lipid metabolism. We identified a novel pathway in which PSMD11 physically interacted with PSMD7 and PSMD14, leading to decreased ubiquitination of the transcription factors SREBP1/2 and PPAR-γ. Notably, PPAR-γ directly bound to the CPT1A promoter to enhance its transcription, thereby promoting fatty acid oxidation (FAO) and oxidative phosphorylation (OXPHOS). In conclusion, our findings uncover a PSMD11–PSMD14–SREBP1/2-PPAR-γ–CPT1A regulatory axis that orchestrates metabolic reprogramming in HCC. Targeting this signaling cascade may represent a promising therapeutic strategy for the precision treatment of HCC, particularly in tumors exhibiting UPS dysregulation.

## Introduction

Hepatocellular carcinoma (HCC) is the sixth most common cancer and the third leading cause of cancer-related mortality worldwide [1]. Although surgical resection remains a curative option for early-stage disease, overall treatment outcomes remain unsatisfactory despite significant advances in molecularly targeted therapies and immunotherapy over the past decade. This is largely due to frequent metastatic progression and limited responsiveness to pharmacological interventions [2, 3]. Therefore, a deeper understanding of the oncogenic signaling pathways driving HCC progression is crucial for developing more effective therapeutic strategies and improving patient outcomes.

The ubiquitin-proteasome system (UPS) plays a pivotal role in regulating intracellular protein degradation and maintaining cellular homeostasis through dynamic post-translational modifications. In tumor biology, ubiquitination has garnered significant attention for its critical impact on the stability and activity of various tumor suppressors and oncogenes [4]. Specifically, ubiquitin-tagged proteins are recognized and degraded by the 26S proteasome, which mediates the majority (over 80%) of regulated protein turnover in proliferating mammalian cells. Notably, specific proteasome subunits, such as PSMD1, have been shown to promote HCC progression by modulating the RTKN/β-catenin/PD-L1 axis [5]. Our previous studies have highlighted the oncogenic roles of specific 26S proteasome subunits in HCC. We demonstrated that PSMD4, a regulatory subunit of the 26S proteasome, promotes tumor progression by stabilizing hypoxia-inducible factor 1 alpha (HIF-1α) via the PTEN/Akt signaling pathway [6]. Furthermore, we found that PSMD9 drives HCC malignancy by interacting with c-Cbl to enhance EGFR signaling and receptor recycling [7]. PSMD11 is a non-ATPase regulatory subunit of the 26S proteasome that facilitates the recognition and processing of ubiquitinated substrates. Accumulating evidence implicates PSMD11 in cancer progression across various malignancies. Specifically, PSMD11 has been shown to promote lung adenocarcinoma progression [8], is overexpressed in relapsed and drug-resistant acute myeloid leukemia (AML) cell lines [9], and serves as a prognostic biomarker in pancreatic ductal adenocarcinoma [10]. In HCC, elevated PSMD11 expression correlates with poor prognosis and enhanced cell proliferation [11, 12], however, the underlying molecular mechanisms remain incompletely understood. In this study, we investigate the functional role of PSMD11 in HCC progression and elucidate the mechanistic pathways by which it drives tumorigenesis.

## Results

### PSMD11 promotes HCC cell proliferation, migration and invasion

**Fig. 1:**
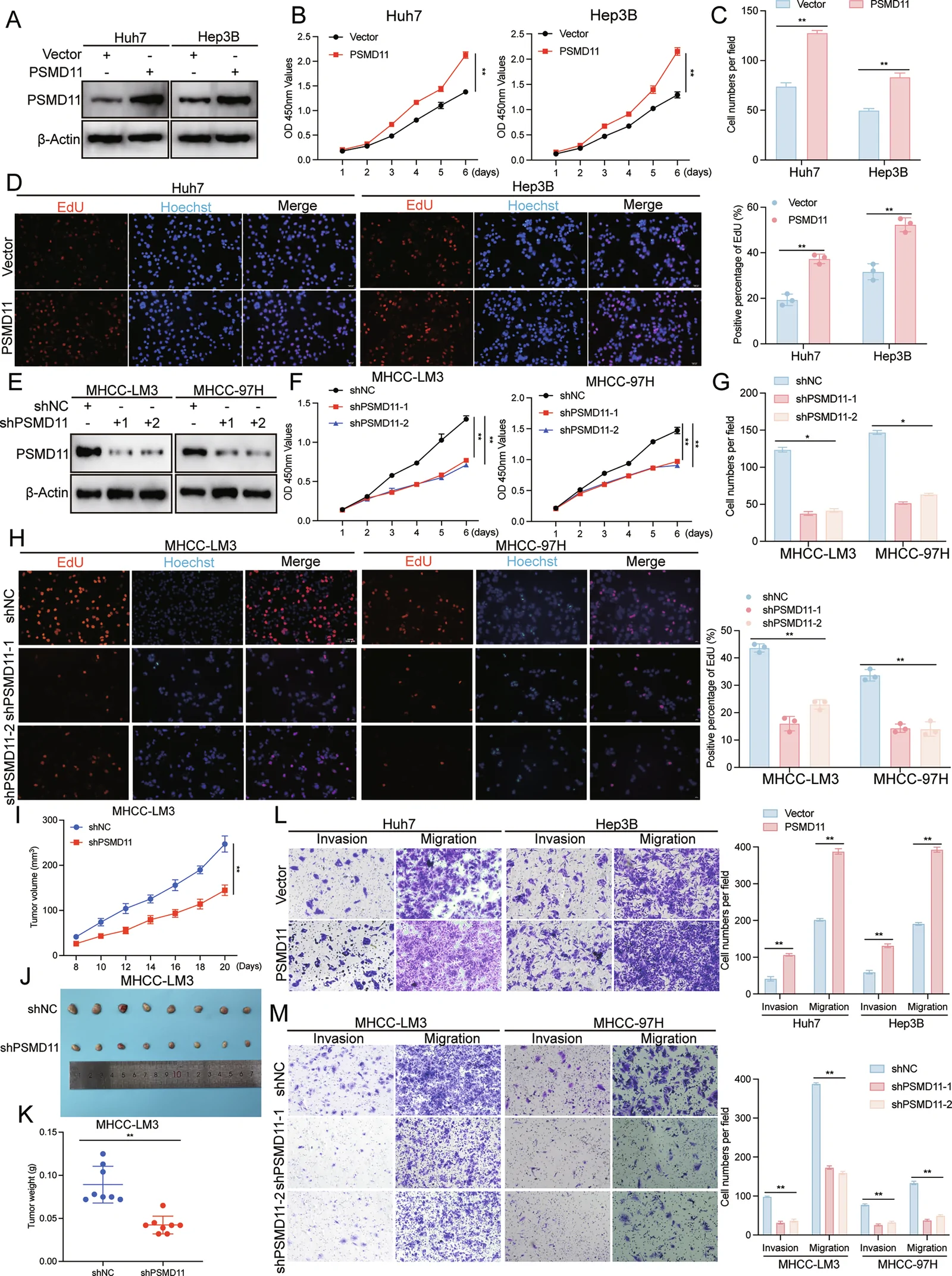
PSMD11 promotes HCC cell proliferation, migration and invasion. **A** Validation of PSMD11 overexpression efficiency by Western blotting in Huh7 and Hep3B cells. Impact of PSMD11 overexpression on proliferation in Huh7 and Hep3B cells, evaluated by CCK-8 assay (**B**), colony formation assay (**C**), and EdU incorporation assay (**D**). **E** Validation of PSMD11 knockdown efficiency by Western blotting following shRNA transduction in MHCC-LM3 and MHCC-97H cells. Impact of PSMD11 knockdown on proliferation in MHCC-LM3 and MHCC-97H cells, evaluated by CCK-8 assay (**F**), colony formation assay (**G**), and EdU incorporation assay (**H**). **I**–**K** Knockdown of PSMD11 inhibits MHCC-LM3 cell growth in vivo. Tumor volume was measured and plotted every 2 days (**I**). **J** Images of resected tumors. **K** The dot plots showed the results of the quantitative analysis of tumor weight. **L** Effects of PSMD11 overexpression on HCC cell migration and invasion in Huh7 and Hep3B cells, assessed by transwell assays. **M** Effects of PSMD11 knockdown on HCC cell migration and invasion in MHCC-LM3 and MHCC-97H cells, assessed by transwell assays. Data were presented as mean ± SD. Student’s t-test was used for comparisons between two groups, while one-way ANOVA was applied for multiple groups. *p < 0.05; **p < 0.01.

To validate the function of PSMD11 in HCC, we first assessed its expression across a panel of HCC cell lines. Based on their endogenous PSMD11 levels (Supplementary Fig. S1), we selected MHCC-97H and MHCC-LM3 cells for loss-of-function studies and Huh7 and Hep3B cells for gain-of-function studies. We found that exogenous expression of PSMD11 markedly promoted cell growth and colony formation (Fig. 1A–D). Conversely, PSMD11 knockdown significantly inhibited HCC cell proliferation and reduced the proportion of EdU-positive cells (Fig. 1E–H and Supplementary Fig. S2). To evaluate the role of PSMD11 in HCC tumorigenesis in vivo, we established a subcutaneous xenograft model in nude mice using MHCC-LM3 cells stably expressing PSMD11-targeting shRNA or control shRNA (shNC). Tumors derived from PSMD11-knockdown cells exhibited significantly suppressed growth, characterized by reduced volumes and lower final weights compared to the control group (Fig. 1I–K). Furthermore, overexpression of PSMD11 facilitated HCC cell migration and invasion, whereas silencing PSMD11 attenuated these malignant phenotypes (Fig. 1L, M).

### Knockdown of PSMD11 induces cell cycle arrest and apoptosis

**Fig. 2:**
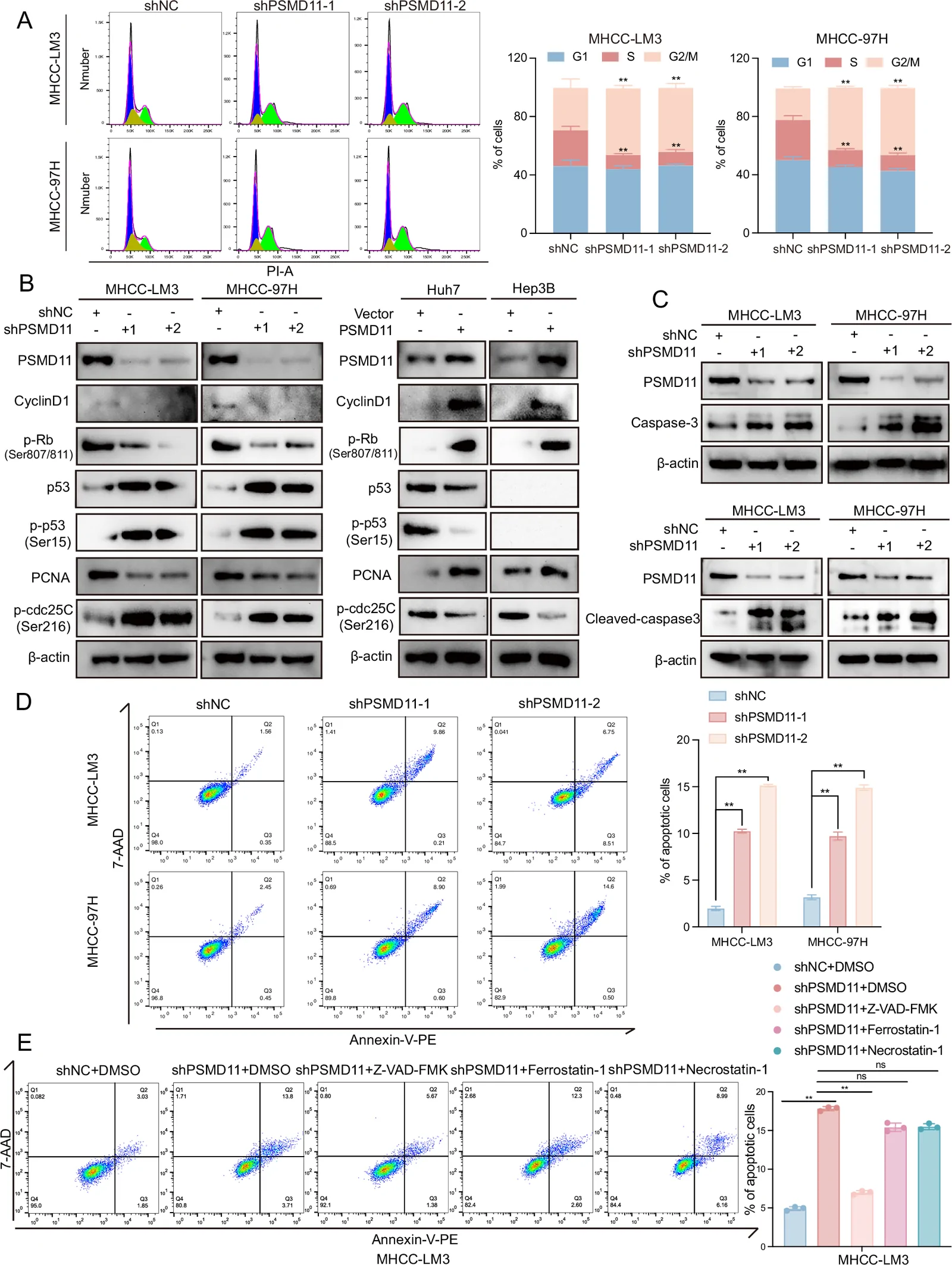
Knockdown of PSMD11 induces cell cycle arrest and apoptosis. **A** Cell cycle distribution analysis of negative control (NC) and PSMD11 knockdown MHCC-97H and MHCC-LM3 cells, as determined by flow cytometry. **B** Western blotting analysis of cell cycle-related protein expression in HCC cells following PSMD11 overexpression (Huh7 and Hep3B) or knockdown (MHCC-97H and MHCC-LM3). **C** Western blotting detection of caspase-3 and cleaved caspase-3 levels in PSMD11 knockdown MHCC-97H and MHCC-LM3 cells. **D** Apoptotic rates were assessed by flow cytometry in PSMD11 knockdown MHCC-97H and MHCC-LM3 cells. **E** Following PSMD11 knockdown, HCC cells were subjected to pharmacological inhibition of distinct cell death modalities using Z-VAD-FMK (10 µM; apoptosis), ferrostatin-1 (2 µM; ferroptosis), and Necrostatin-1 (10 µM; necroptosis). Apoptotic rates were assessed by flow cytometry. Data were shown as mean ± SD. Student’s t-test was used for comparisons between two groups, while one-way ANOVA was applied for multiple groups. **p < 0.01.

To investigate the mechanism by which PSMD11 regulates HCC proliferation, we examined its effects on the cell cycle progression and apoptosis. Knockdown of PSMD11 resulted in a significant accumulation of cells in the G2/M phase and a concomitant reduction in the S phase (Fig. 2A). Consistent with this cell cycle arrest, PSMD11 knockdown led to a downregulation of phosphorylated Rb, a key regulatory protein, compared to controls. The expression of phosphorylated p53 and cdc25c was increased in PSMD11-depleted cells. Meanwhile, PCNA and Cyclin D1 was decreased in PSMD11-knockdown cells (Fig. 2B). PSMD11 overexpression resulted in the opposite effects (Fig. 2B). Furthermore, PSMD11 knockdown markedly induced apoptosis, evidenced by increased levels of cleaved caspase-3 (Fig. 2C, D). To definitively identify the specific mode of cell death, we treated PSMD11-knockdown cells with selective inhibitors: Z-VAD-FMK (pan-caspase inhibitor for apoptosis), Ferrostatin-1 (for ferroptosis), and Necrostatin-1 (Nec-1; for necroptosis). Notably, only Z-VAD-FMK significantly rescued cell viability, whereas Ferrostatin-1 and Nec-1 conferred no protective effect (Fig. 2E). Collectively, these findings indicate that PSMD11 promotes HCC proliferation by driving cell cycle progression and suppressing apoptosis, thereby facilitating tumor progression.

### PSMD11 potentiates lipid and cholesterol metabolism in HCC cells

**Fig. 3:**
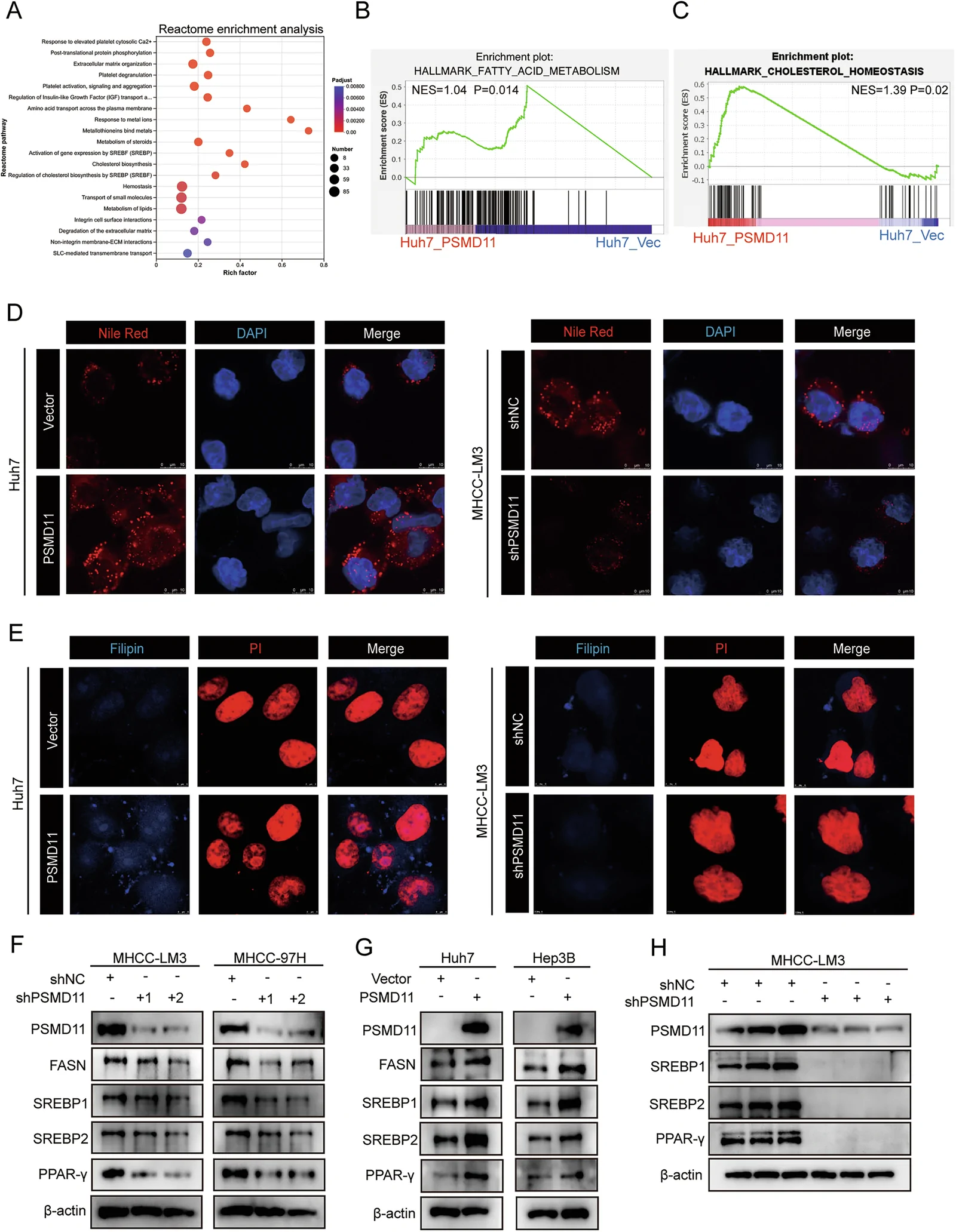
PSMD11 reprogramms lipid metabolism. **A** Reactome pathway analysis of RNA-seq data from Huh7 cells overexpressing PSMD11. GSEA of transcriptomes from PSMD11-overexpressing Huh7 cells reveals fatty acid metabolism (**B**) and cholesterol homeostasis (**C**) as top enriched pathways. **D** Representative images showing lipid droplets stained with Nile Red in PSMD11-overexpressing Huh7 cells or PSMD11-knockdown MHCC-LM3 cells. **E** Representative images showing intracellular cholesterol stained with Filipin in PSMD11-overexpressing Huh7 cells or PSMD11-knockdown MHCC-LM3 cells. **F** Western blotting analysis of FASN, SREBP1/2, and PPAR-γ protein levels in HCC cells with PSMD11 knockdown (MHCC-97H and MHCC-LM3). **G** Western blotting analysis of FASN, SREBP1/2, and PPARγ protein levels in HCC cells with PSMD11 overexpression (Huh7 and Hep3B). **H** The protein levels of PSMD11, SREBP1/2, and PPAR-γ in PSMD11-knockdown MHCC-LM3 xenograft tissues were assessed by Western blotting.

To elucidate the molecular mechanism of PSMD11 in HCC, RNA sequencing was performed in PSMD11-overexpressing HCC cells. Analysis of differentially expressed genes (DEGs) via Reactome pathway enrichment revealed a significant overrepresentation of pathways related to lipid and cholesterol metabolism (Fig. 3A). Consistently, Gene Set Enrichment Analysis (GSEA) demonstrated a positive correlation between PSMD11 expression and genes involved in fatty acid metabolism and cholesterol homeostasis (Fig. 3B, C). Given these findings, we next investigated the functional impact of PSMD11 on lipid metabolism. We observed that PSMD11 overexpression markedly increased intracellular lipid droplet accumulation and cholesterol levels, whereas PSMD11 knockdown suppressed these effects (Fig. 3D, E).

Fatty acid synthase (FASN) is a key lipogenic enzyme that catalyzes the terminal step in de novo fatty acid biosynthesis and is essential for maintaining lipid homeostasis [13]. We found that PSMD11 overexpression upregulated FASN expression, while PSMD11 knockdown reduced FASN expression (Fig. 3F). Since sterol regulatory element-binding proteins (SREBP1 and SREBP2) and peroxisome proliferator-activated receptor gamma (PPAR-γ) are master transcription factors regulating fatty acid and cholesterol synthesis [14, 15]. Furthermore, SREBP1 directly binds to the promoter region of FASN to activate its transcription [16]. We therefore examined their expression in PSMD11-modulated cells. Notably, PSMD11 overexpression increased the levels of SREBP1, SREBP2, and PPAR-γ, whereas PSMD11 knockdown decreased their expression (Fig. 3F, G). These findings were further corroborated in PSMD11-knockdown MHCC-LM3 xenograft tissues (Fig. 3H). Collectively, these results indicate that PSMD11 drives lipid and cholesterol metabolism in HCC cells by upregulating the SREBP/PPARγ axis and its downstream target, FASN.

### PSMD11 promotes HCC cells proliferation and invasion through CPT1A-mediated fatty acid oxidation

**Fig. 4:**
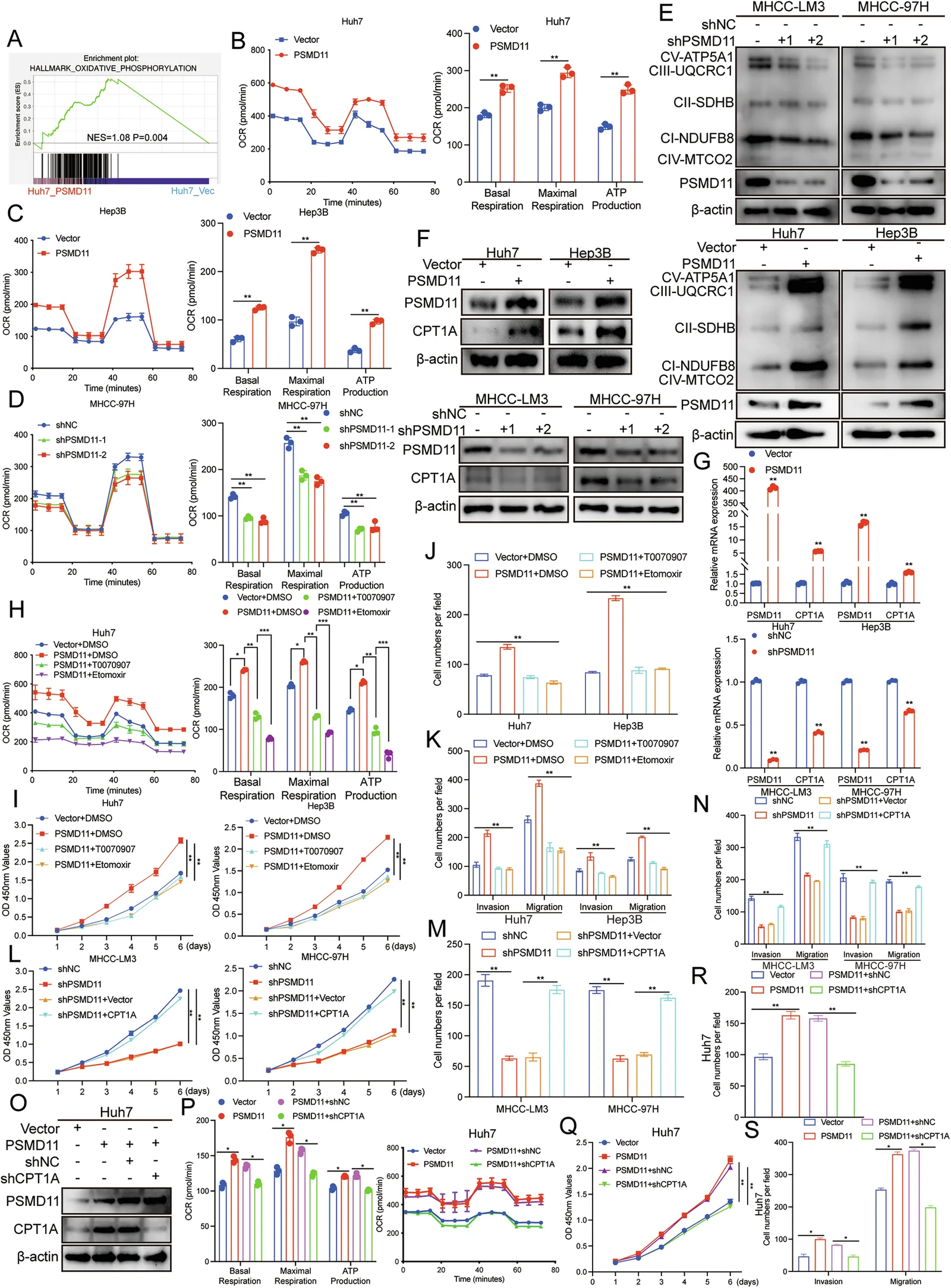
PSMD11 promotes HCC cells proliferation and invasion through CPT1A-mediated fatty acid oxidation. **A** GSEA of transcriptomes from PSMD11-overexpressing Huh7 cells reveals enrichment of OXPHOS pathways. The OCR, basal mitochondrial respiration rate, maximal respiratory capacity and ATP production were calculated and statistically analyzed in PSMD11 overexpressing (Huh7 and Hep3B) (**B**, **C**) and PSMD11 knockdown MHCC-97H cells (**D**). **E** OXPHOS complexes were detected by Western Blotting in PSMD11 knockdown MHCC-97H and MHCC-LM3 cells or PSMD11 overexpression Huh7 and Hep3B cells. The expression of CPT1A protein (**F**) and mRNA (**G**) level was detected by Western Blotting and qRT-PCR. **H** The OCR, basal mitochondrial respiration rate, maximal respiratory capacity and ATP production were calculated and statistically analyzed in PSMD11 overexpressing Huh7 cells treated with etomoxir and T0070907. PSMD11-overexpressing HCC cells were treated with etomoxir or DMSO as indicated, and cell proliferation, migration, and invasion were evaluated by CCK-8 assay (**I**), colony formation (**J**), and Transwell assays (**K**). PSMD11-knockdown HCC cells were transfected with CPT1A or Vector as indicated, and cell proliferation, migration, and invasion were evaluated by CCK-8 assay (**L**), colony formation (**M**), and Transwell assays (**N**). **O**–**S** PSMD11-overexpressing HCC cells were transfected with CPT1A shRNA or shNC. The expression of CPT1A protein level was detected by Western Blotting (**O**). The OCR, basal mitochondrial respiration rate, maximal respiratory capacity and ATP production were calculated and statistically analyzed (**P**). Cell proliferation was evaluated by CCK-8 assay (**Q**) and colony formation (**R**). Migration and invasion were evaluated by Transwell assays (**S**). Data were shown as mean ± SD. Student’s t-test was used for comparisons between two groups, while one-way ANOVA was applied for multiple groups. *p < 0.05; **p < 0.01.

In human HCC cells, fatty acid oxidation (FAO) is highly active and serves to replenish the mitochondrial acetyl-CoA pool required for oxidative phosphorylation (OXPHOS) [17]. Furthermore, GSEA demonstrated a positive correlation between PSMD11 expression and OXPHOS-related gene signatures (Fig. 4A). To functionally validate these findings, we measured mitochondrial respiration using the Seahorse XF Analyzer. PSMD11 overexpression significantly increased basal respiration, maximal respiratory capacity, and intracellular ATP levels, whereas PSMD11 knockdown markedly suppressed oxygen consumption rates (OCR) (Fig. 4B–D). Furthermore, the expression of key OXPHOS complex subunits-including NDUFB8 (Complex I), SDHB (Complex II), UQCRC1 (Complex III), MTCO2 (Complex IV), and ATP5A1 (Complex V), was significantly upregulated upon PSMD11 overexpression and downregulated upon PSMD11 knockdown in HCC cells (Fig. 4E). These data suggest that PSMD11 drives mitochondrial bioenergetics by coordinating the expression of essential electron transport chain components.

Carnitine palmitoyltransferase 1 A (CPT1A), the rate-limiting enzyme of FAO located on the mitochondrial outer membrane, facilitates the transport of long-chain fatty acids into mitochondria for β-oxidation [18]. We found that PSMD11 overexpression increased CPT1A expression at both the mRNA and protein levels, whereas PSMD11 knockdown suppressed its expression (Fig. 4F, G). Immunoblotting analysis of xenograft tissues further revealed that PSMD11 knockdown led to a marked downregulation of CPT1A (Supplementary Fig. S3). To determine whether CPT1A is functionally involved in PSMD11-mediated metabolic reprogramming, we treated cells with etomoxir, a specific CPT1A inhibitor (Selleck, Houston, TX, USA). Etomoxir treatment effectively abrogated the PSMD11 overexpression-induced increases in basal respiration, maximal respiration, and intracellular ATP production (Fig. 4H). Notably, the pro-tumorigenic effects of PSMD11 overexpression-enhanced cell proliferation, migration, and invasion were attenuated by etomoxir treatment (Fig. 4I–K and Supplementary Fig. S4A, B). To further validate CPT1A-mediated FAO as the key downstream mechanism of PSMD11-driven tumorigenesis, we conducted rescue experiments by ectopic overexpression of CPT1A in PSMD11-knockdown cells. We observed that CPT1A overexpression partially rescued the suppressive effects of PSMD11 silencing on these phenotypes (cell proliferation, migration and invasion, Fig. 4L–N and Supplementary Fig. S4C, D). Conversely, CPT1A knockdown not only weakened mitochondrial function (basal/maximal respiration and ATP levels) but also abrogated the pro-tumorigenic effects of PSMD11, including cell proliferation, migration, and invasion (Fig. 4O–S and Supplementary Fig. S5). Collectively, these findings demonstrate that PSMD11 drives HCC progression by upregulating CPT1A to enhance FAO and mitochondrial OXPHOS.

### PPAR-γ inhibitor suppresses PSMD11-mediated OXPHOS and tumor promotion

To determine whether PPAR**-**γ is functionally involved in PSMD11-mediated OXPHOS and tumor promotion, we treated cells with T0070907, a specific PPAR**-**γ inhibitor (Selleck, Houston, TX, USA). Pharmacological inhibition of PPAR-γ effectively abrogated the PSMD11-induced enhancements in basal respiration, maximal respiration, and intracellular ATP production (Fig. 4H). Notably, the pro-tumorigenic effects of PSMD11 overexpression-enhanced cell proliferation, migration, and invasion were attenuated by T0070907 treatment (Fig. 4I, K and Supplementary Fig. S4A, B). Collectively, these findings demonstrate that PSMD11 promotes fatty acid oxidation and mitochondrial metabolism in HCC cells by upregulating PPAR**-**γ, thereby enhancing OXPHOS and supporting tumor progression.

### PSMD11 binds with and stabilizes PSMD14 protein to increase SREBP1/2 and PPAR-γ expression

**Fig. 5:**
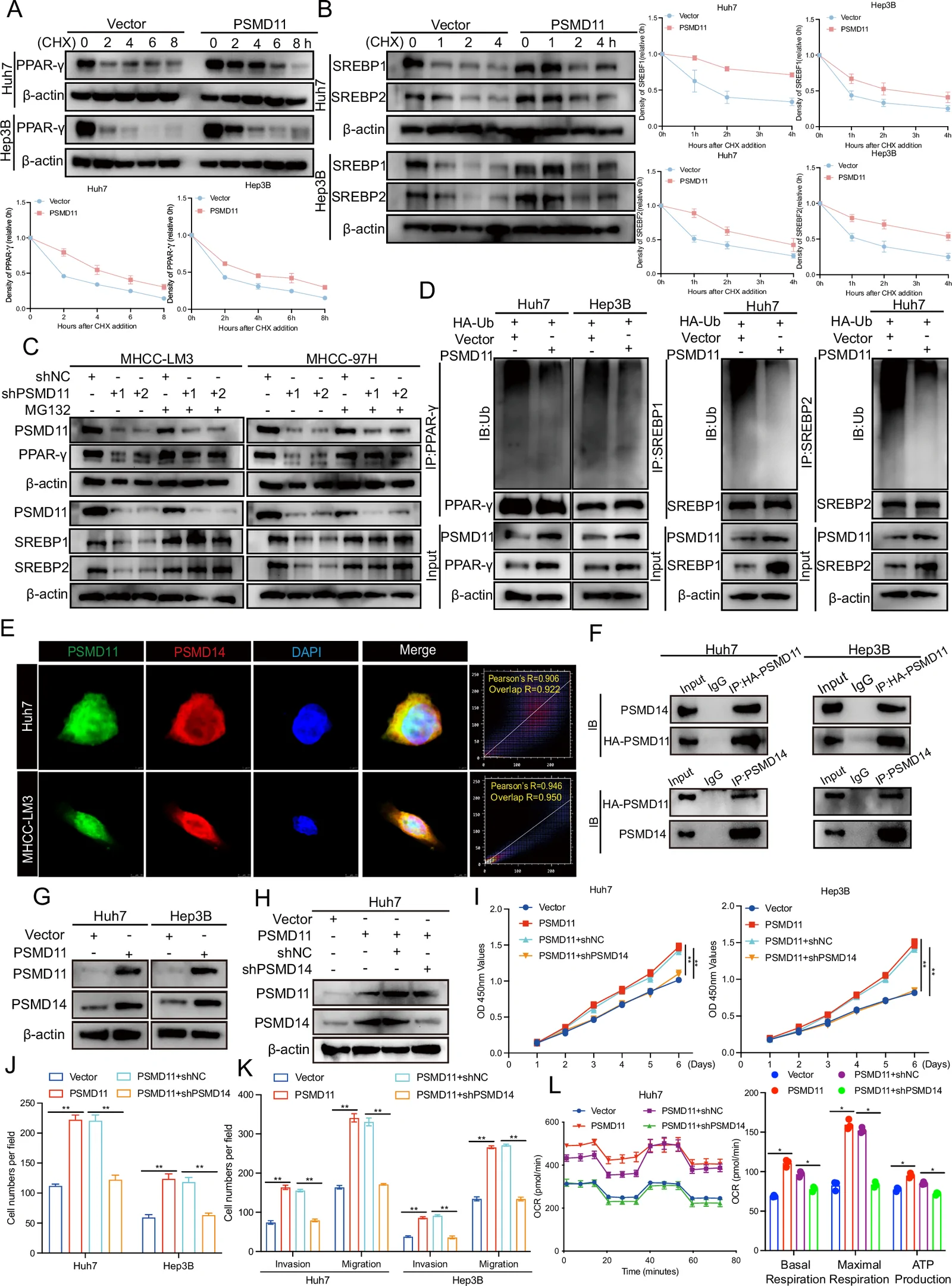
PSMD11 interacts PSMD14 and is associated with SREBP1/2 and PPAR-γ deubiquitination. Expression of PPAR-γ (**A**) and SREBP1/2 (**B**) in PSMD11-overexpressing Huh7 and Hep3B cells treated with cycloheximide (CHX) at the indicated time points. **C** Expression of SREBP1/2 and PPAR-γ in PSMD11-knockdown MHCC-LM3 and MHCC-97H cells treated with MG132 for 6 h. **D** Lysates of PSMD11-overexpressing and control Huh7 and Hep3B cells were immunoprecipitated with antibody for SREBP1/2 and PPAR-γ, and the immunocomplexes were immunoblotted with antibodies against ubiquitinated proteins. **E** Colocalization of PSMD11 and PSMD14 in HCC cells. **F** PSMD11 interacts with PSMD14 in HCC cells. **G** The effects of PSMD11 on the expression of PSMD14 in Huh7 and Hep3B cells. **H**–**L** PSMD11-overexpressing HCC cells were transfected with PSMD14 shRNA or shNC. The expression of PSMD14 protein level was detected by Western Blotting (**H**). Cell proliferation was evaluated by CCK-8 assay (**I**) and colony formation (**J**). Migration, and invasion were evaluated by Transwell assays (**K**). The OCR, basal mitochondrial respiration rate, maximal respiratory capacity and ATP production were calculated and statistically analyzed (**L**). Data are presented as mean ± SD and were analyzed by one-way ANOVA. *p < 0.05; **p < 0.01.

While our results demonstrate that PSMD11 regulates the protein expression of SREBP1, SREBP2, and PPAR-γ, we observed no corresponding changes in their mRNA levels upon PSMD11 overexpression or knockdown (Supplementary Fig. S6). This dissociation suggests that PSMD11 regulates these transcription factors via a post-translational mechanism. To test whether PSMD11 modulates the stability of these transcription factors, we performed cycloheximide (CHX) chase assays to block de novo protein synthesis. In HCC cells stably overexpressing PSMD11, the degradation rates of SREBP1, SREBP2, and PPAR-γ were significantly attenuated compared to vector controls (Fig. 5A, B), resulting in markedly extended protein half-lives. Collectively, these data indicate that PSMD11 promotes the accumulation of SREBP1, SREBP2, and PPAR-γ by enhancing their protein stability.

To determine whether the UPS mediates the degradation of these factors, we treated PSMD11-knockdown cells with MG132, a reversible proteasome inhibitor. MG132 treatment effectively abrogated the reduced protein levels of SREBP1, SREBP2, and PPAR-γ in PSMD11-deficient cells (Fig. 5C), indicating that PSMD11 protects these proteins from proteasomal degradation. Consistent with this protective role, ubiquitination assays revealed that PSMD11 overexpression significantly attenuated the levels of ubiquitinated SREBP1, SREBP2, and PPAR-γ (Fig. 5D), whereas PSMD11 knockdown increased their polyubiquitination (Supplementary Fig. S7). Together, these data demonstrate that PSMD11 stabilizes SREBP1, SREBP2, and PPAR-γ by suppressing their ubiquitin-proteasome-mediated degradation.

To explore the molecular mechanism underlying PSMD11-mediated stabilization, we used GeneMANIA (https://genemania.org/) to identify potential PSMD11-interacting proteins (Supplementary Fig. 8A). Integrating these results with data from deubiquitinase databases, we identified 2 candidate regulators, with PSMD14 emerging as a top hit due to its known role in the 19S proteasome regulatory particle and deubiquitinating activity (Supplementary Fig. 8B). Immunofluorescence staining showed strong co-localization of PSMD11 and PSMD14 in the Huh7 and MHCC-LM3 cells (Fig. 5E). Furthermore, Co-immunoprecipitation (Co-IP) assays confirmed a direct physical interaction between PSMD11 and PSMD14 (Fig. 5F).Notably, PSMD11 overexpression increased PSMD14 protein expression (Fig. 5G). Immunoblotting analysis of xenograft tissues further revealed that PSMD11 knockdown led to a marked downregulation of PSMD14 (Supplementary Fig. S2). Additionally, Co-IP assays revealed a direct interaction between PSMD11 and PSMD7. Furthermore, PSMD11 overexpression upregulated PSMD7 protein levels, whereas PSMD11 knockdown downregulated its expression (Supplementary Fig. S8C, D). PSMD7, an ATP-independent subunit of the 19S regulatory particle, heterodimerizes with the catalytic subunit PSMD14 to facilitate ubiquitinated substrate processing. While PSMD7 acts as a structural ‘pseudo-deubiquitinase’ lacking catalytic activity, its partnership with PSMD14 is essential for function [19]. Consequently, we propose that PSMD11 associates with the PSMD7–PSMD14 heterodimer to form a functional module. In this context, PSMD7 likely provides the structural scaffolding necessary to stabilize PSMD14, enabling its deubiquitinase activity on downstream targets.

**Fig. 6:**
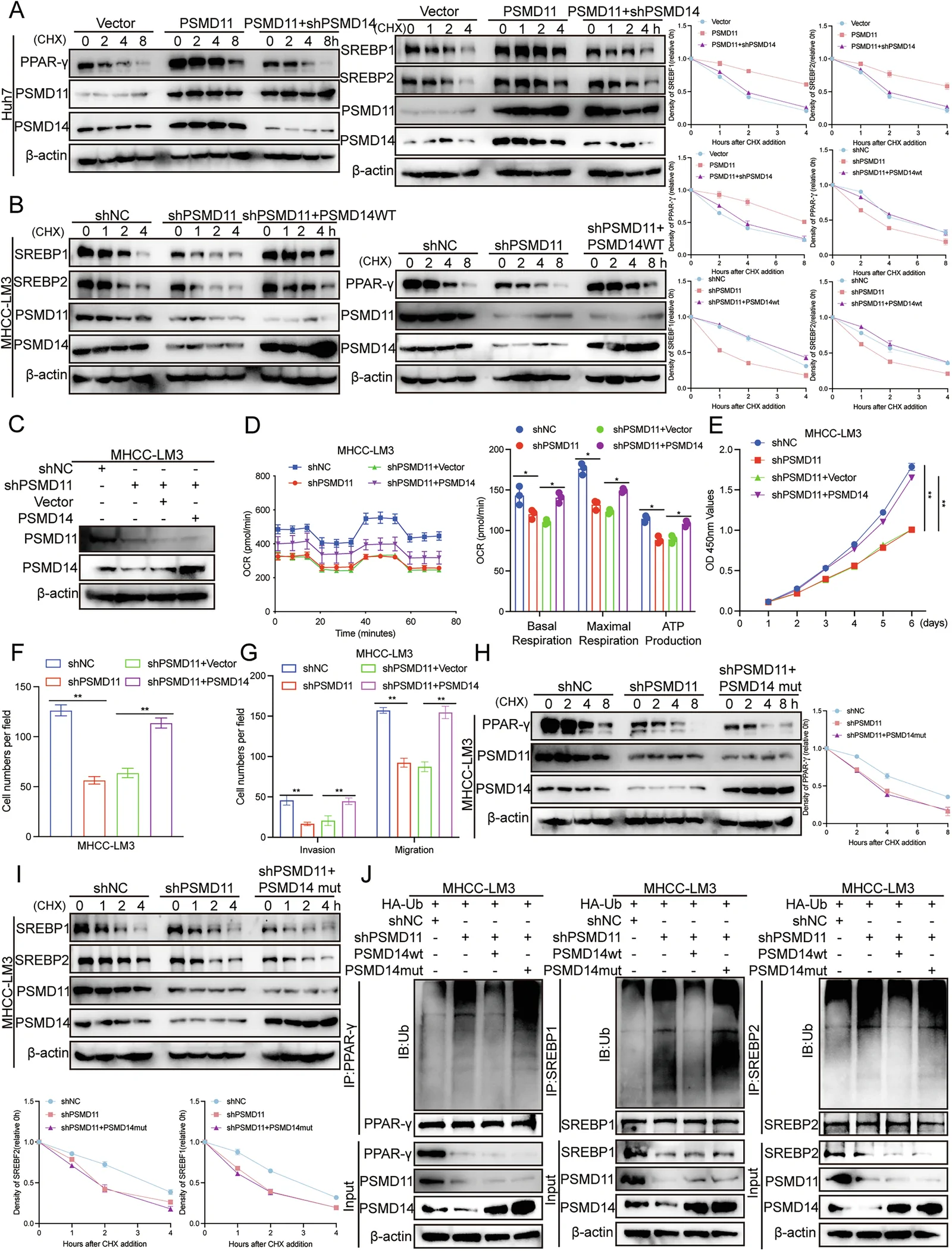
PSMD11 enhances the protein stability of SREBP1/2 and PPAR-γ in a PSMD14 deubiquitinase activity-dependent manner. **A** Huh7 cells overexpressing PSMD11 were co-transfected with PSMD14 shRNA or shNC and treated with cycloheximide (CHX) for the indicated time periods. The expression levels of PPAR-γ and SREBP1/2 were subsequently analyzed. **B** MHCC-LM3 cells with PSMD11 knockdown were transfected with PSMD14 or vector and subjected to CHX treatment. Expression of PPAR-γ and SREBP1/2 was monitored over time. **C**–**G** Functional rescue assays in PSMD11-knockdown MHCC-LM3 cells. **C** Western blot analysis confirmed PSMD14 protein expression. **D** Mitochondrial function was assessed by measuring OCR, basal respiration, maximal respiratory capacity, and ATP production. **E**, **F** Cell proliferation was evaluated by CCK-8 and colony formation assays, respectively. **G** Transwell assays were performed to assess migration and invasion. **H**, **I** PSMD11-knockdown MHCC-LM3 cells were transfected with catalytically inactive PSMD14. The expression of PPAR-γ (**H**) and SREBP1/2 (**I**) was determined following CHX treatment. **J** Lysates from cells transfected with wild-type or catalytically inactive PSMD14 were subjected to immunoprecipitation using antibodies against SREBP1/2 and PPAR-γ. The immunocomplexes were then immunoblotted with an anti-ubiquitin antibody. Data are presented as mean ± SD and were analyzed by one-way ANOVA. **p < 0.01.

To determine whether PSMD14 mediates PSMD11-dependent substrate stabilization and phenotypic outcomes, we conducted rescue experiments in PSMD11-overexpressing HCC cells. PSMD14 knockdown significantly attenuated the functional phenotypes induced by PSMD11 overexpression, including enhanced cell proliferation, migration, invasion, and OXPHOS (Fig. 5H–L and Supplementary Fig. S9). Furthermore, we found that PSMD11 overexpression markedly delay the degradation of SREBP1, SREBP2, and PPAR-γ, knockdown of PSMD14 accelerated the degradation of these proteins (Fig. 6A).

### PSMD11 promotes SREBP1/2 and PPAR-γ stability through PSMD14-mediated deubiquitination

Previous structural and functional studies have established that the JAMM domain of PSMD14, particularly residues H113, is critical for its deubiquitinating enzymatic activity [20]. To confirm the role of PSMD14 deubiquitinating enzymatic activity in maintaining SREBP1/2 and PPAR-γ stability, we transfected PSMD14-H113Q mutant into HCC cells. We observed that wild-type PSMD14 overexpression rescued PSMD11 silencing-induced impairments in SREBP1/2 and PPAR-γ stability, as well as in OXPHOS, cell proliferation, migration, and invasion (Fig. 6B–G and Supplementary Fig. S10). However, overexpression of the catalytically inactive PSMD14-H113Q mutant failed to restore protein stability (Fig. 6H, I). In addition, ubiquitination assays revealed that PSMD11 knockdown significantly increased the levels of ubiquitinated SREBP1/2 and PPAR-γ. Notably, this effect was reversed by wild-type PSMD14, but not by the PSMD14-H113Q mutant (Fig. 6J). Furthermore, Co-IP assays demonstrated that PSMD14 directly interacts with SREBP1/2 and PPAR-γ (Supplementary Fig. S11). Collectively, these findings demonstrate that PSMD11 promotes SREBP1/2 and PPAR-γ stability through PSMD14-mediated deubiquitination.

Given the crucial role of PSMD14 deubiquitinase activity, we hypothesized that targeting PSMD14 could inhibit HCC progression. In vitro assays demonstrated that the PSMD14 deubiquitinase inhibitor Capzimin suppressed HCC cell proliferation, migration, and invasion. To validate these findings in vivo, we established a subcutaneous xenograft model and confirmed that Capzimin significantly impeded HCC tumor growth (Supplementary Fig. S12).

### PPAR-γ directly binds to the CPT1A promoter and induces its expression in HCC cells

**Fig. 7:**
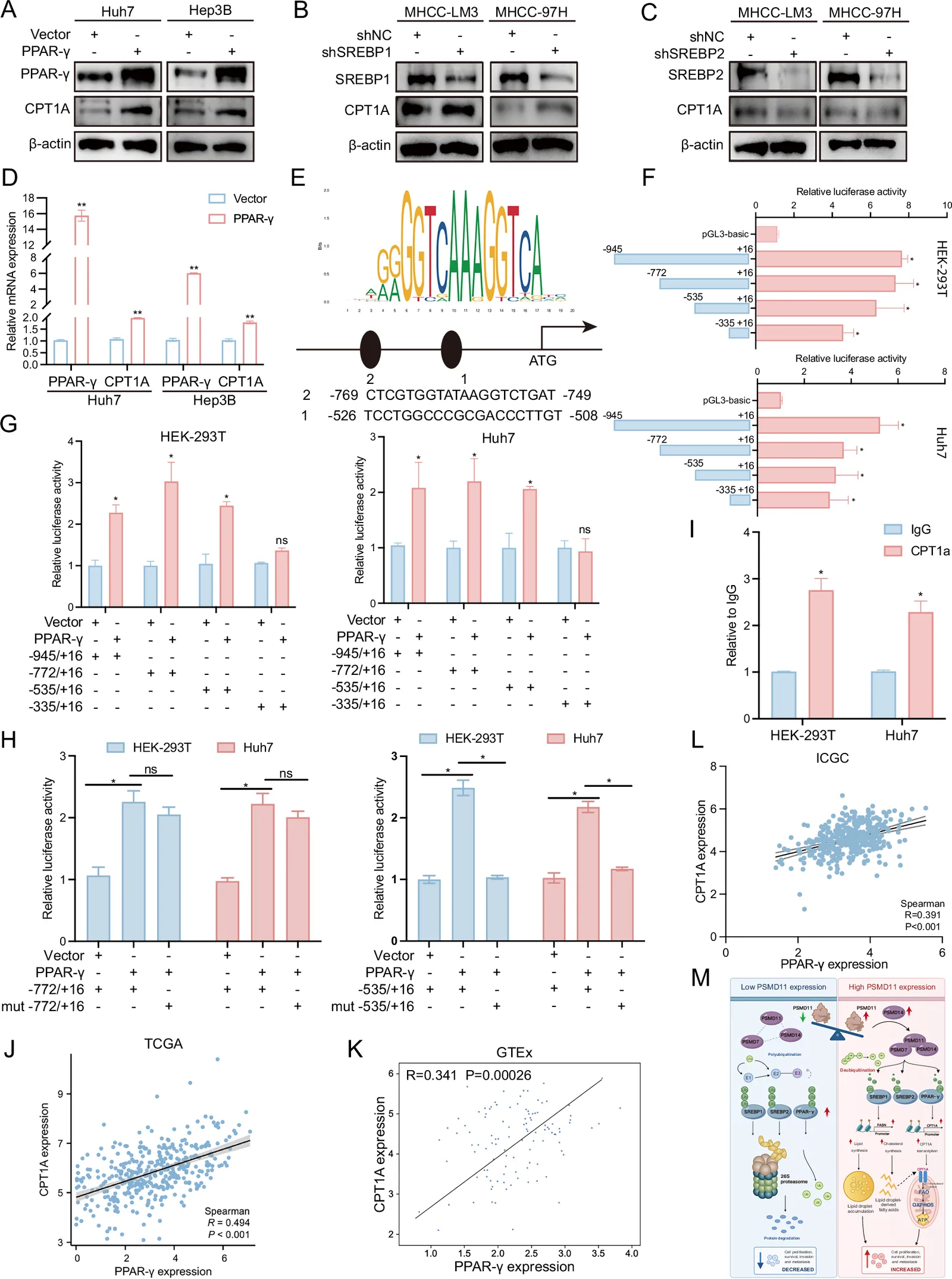
PPAR-γ binds to the CPT1A promoter and increases its expression. **A** Expression of CPT1A in PPAR-γ-overexpressing HCC cells. **B**, **C** Expression of CPT1A in SREBP1/2-knockdown HCC cells. **D** Expression of CPT1A mRNA in PPAR-γ-overexpressing HCC cells. **E** PPAR-γ binding motif. **F** Huh7 and HEK-293T cells were transfected with different luciferase reporter vectors. The corresponding luciferase activities were measured via reporter gene assays. **G** Huh7 and HEK-293T cells were transfected with CPT1A luciferase reporter vectors and PPAR-γ. The corresponding relative luciferase activities were determined by reporter gene assays. **H** Huh7 and HEK-293T cells were transfected with CPT1A luciferase reporter vectors (wild-type or mutant PPAR-γ-binding sites) and PPAR-γ. The corresponding relative luciferase activities were determined by reporter gene assays. **I** ChIP followed by qRT-PCR analysis was performed to evaluate PPAR-γ binding to the CPT1A promoter. The correlation between PPAR-γ and CPT1A expression in HCC tissues using TCGA data (**J**), GTEx (**K**) and ICGC (**L**). **M** Proposed model of PSMD11 mechanisms in HCC progression. PSMD11 physically interacts with PSMD7 and PSMD14, resulting in decreased ubiquitination of the transcription factors SREBP1/2 and PPAR-γ. SREBP1 subsequently binds to the *FASN* promoter to enhance lipid and cholesterol synthesis. Concurrently, PPAR-γ directly binds to the *CPT1A* promoter to promote FAO and oxidative phosphorylation OXPHOS. These metabolic alterations collectively drive HCC progression. Consequently, targeting PSMD11 represents a potential strategy to inhibit HCC progression. Data are presented as mean ± SD and were analyzed by one-way ANOVA. *p < 0.05.

Having established that PSMD11 drives HCC progression via CPT1A activation, we sought to identify the upstream transcriptional regulator responsible for *CPT1A* induction. To determine whether SREBP1/2 or PPAR-γ directly regulate CPT1A expression, we first evaluated their effects on CPT1A expression in HCC cells. While neither SREBP1 nor SREBP2 significantly altered *CPT1A* mRNA or protein levels, PPAR-γ markedly upregulated *CPT1A* expression at both levels (Fig. 7A–D), implicating PPAR-γ as a direct transcriptional activator of *CPT1A* in HCC.

To test this hypothesis, we cloned the CPT1A promoter into the pGL3-enhancer luciferase vector. Bioinformatic analysis (JASPAR v3.0) identified two putative PPAR-γ binding motifs within the proximal promoter region (–945 to +16 bp; Fig. 7E). We generated a series of 5′-truncated promoter constructs based on these motifs (Fig. 7F).

Luciferase assays showed that PPAR-γ enhanced promoter activity in all constructs except for the shortest construct (–335/ + 16) (Fig. 7G), indicating that the critical PPAR-γ-responsive region lies between –772 and –335 bp upstream of the transcription start site. Notably, mutation of putative PPAR-γ-binding site 1 (but not sites 2) abolished PPAR-γ-induced promoter activation (Fig. 7H), suggesting that this site is essential for PPAR-γ-mediated transactivation. Chromatin immunoprecipitation (ChIP) assays further confirmed that PPAR-γ directly occupies the endogenous *CPT1A* promoter in HCC cells in vivo (Fig. 7I). Finally, to assess the clinical relevance of this regulatory axis, we examined the correlation between PPAR-γ and CPT1A expression in human HCC tissues. A significant positive correlation was observed, further supporting the physiological relevance of PPAR-γ-driven CPT1A expression in HCC (Fig. 7J–L).

### Elevated PSMD11, PSMD14 and PPAR-γ expression predicts poor prognosis in HCC

**Fig. 8:**
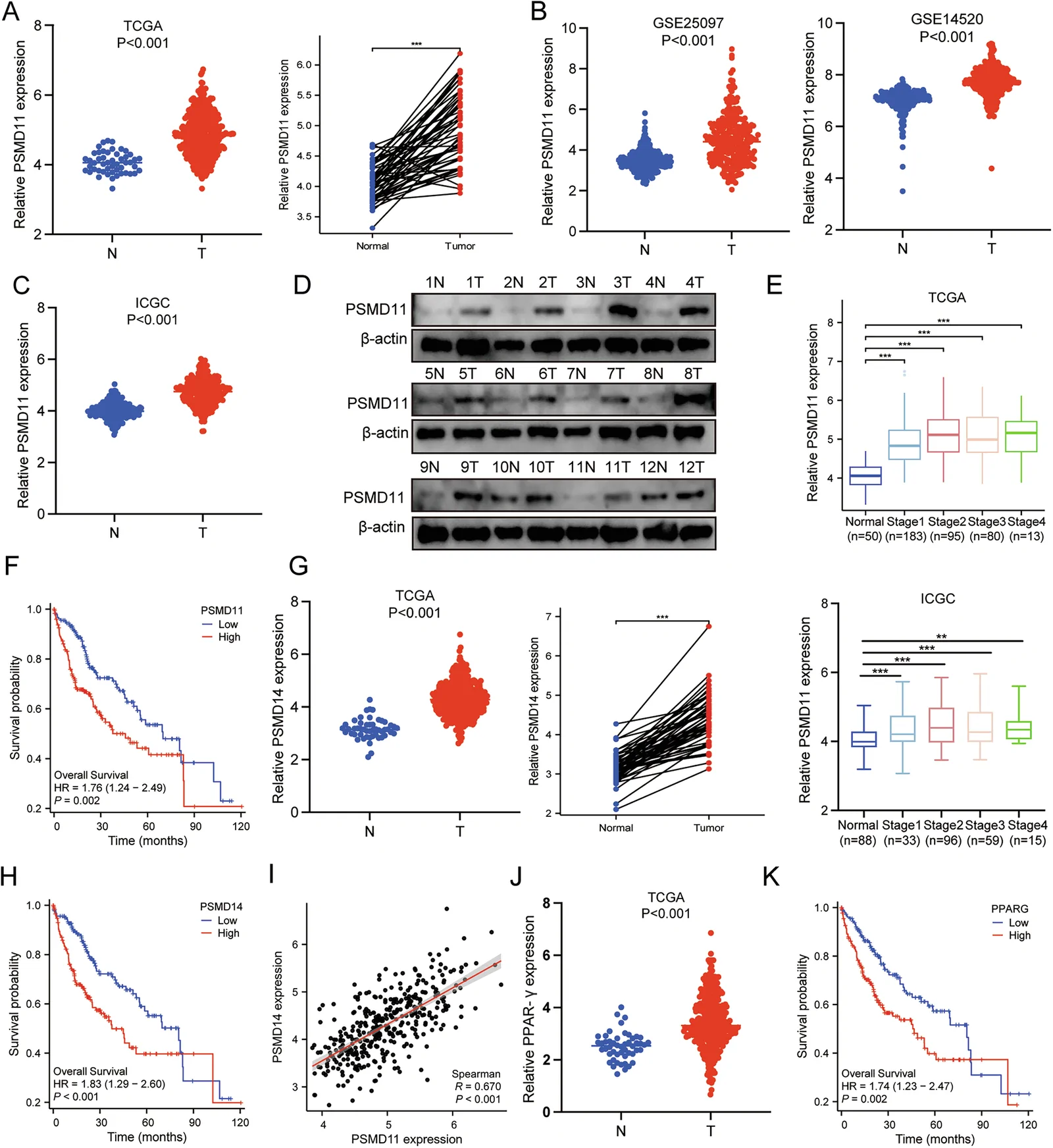
Overexpression of PSMD11, PSMD14 and PPAR-γ is associated with a poor prognosis in HCC patients. **A**–**D** The expression of PSMD11 in HCC tissues was compared with that in the corresponding noncancerous liver tissues in the TCGA datasets (**A**), the GSE25097 and GSE14520 (**B**) and the ICGC-LIRI-JP dataset (**C**). **D** The expression of PSMD11 was detected by Western blotting in HCC tissues and adjacent non-tumor tissues. **E** The expression of PSMD11 in HCC tissues of different stages was analyzed in the TCGA. **F** Overall survival analysis of HCC patients by the PSMD11 expression level in the TCGA cohort. Kaplan–Meier method and Log-rank test. **G** The expression of PSMD14 in HCC tissues was compared with that in the corresponding noncancerous liver tissues in the TCGA datasets. **H** Overall survival analysis of HCC patients by the PSMD14 expression level in the TCGA cohort. Kaplan–Meier method and Log-rank test. **I** The correlation between PSMD11 and PSMD14 expression in HCC tissues using TCGA data. **J** The expression of PPAR-γ in HCC tissues was compared with that in the corresponding noncancerous liver tissues in the TCGA datasets. **K** Overall survival analysis of HCC patients by the PPAR-γ expression level in the TCGA cohort. Kaplan–Meier method and Log-rank test. Data were shown as mean ± SD. The data were analyzed by two-tailed unpaired t-test.*p < 0.05; **p < 0.01, **p < 0.001.

To investigate the clinical relevance of PSMD11, PSMD14, and PPAR-γ in HCC, we analyzed their expression using datasets from The Cancer Genome Atlas (TCGA), the Gene Expression Omnibus (GEO), and the ICGC-LIRI-JP project. Across multiple cohorts, *PSMD11* mRNA levels were significantly elevated in HCC tissues compared to paired non-tumor liver tissues (Fig. 8A–C). This upregulation was further confirmed at the protein level by Western blotting, which showed marked PSMD11 overexpression in HCC specimens relative to adjacent normal tissues (Fig. 8D). Moreover, PSMD11 expression exhibited a positive correlation with tumor malignancy stage and grade in both the TCGA and ICGC-LIRI-JP cohorts (Fig. 8E and Supplementary Fig. S13A). Kaplan–Meier survival analysis revealed that patients with high PSMD11 expression exhibited significantly shorter overall survival (OS), disease-free survival (DFS), progression-free survival (PFS), and disease-specific survival (DSS**)** compared to those with low expression (Fig. 8F and Supplementary Fig. S13B–D). Similarly, transcriptomic analysis of TCGA data demonstrated that both PSMD14 and PPAR-γ were significantly upregulated in HCC tissues compared to paired controls (Fig. 8G, J). High expression of either PSMD14 or PPAR-γ was also strongly associated with poor OS (Fig. 8H, K). Notably, we observed a significant positive correlation between PSMD11 and PSMD14 expression levels in HCC tissues (Fig. 8I), supporting the notion that these proteasome subunits are co-regulated and collectively contribute to HCC progression.

## Discussion

In this study, we demonstrate that upregulated PSMD11 drives HCC progression by reprogramming lipid metabolism through the stabilization of SREBP1/2 and PPAR-γ. Mechanistically, we demonstrate that PSMD11 forms a functional complex with both PSMD7 and PSMD14. Within this assembly, PSMD7 likely serves as a structural scaffold, while PSMD14 provides the catalytic deubiquitinase activity required to stabilize downstream substrates. Specifically, PSMD14 interacts with SREBP1/2 and PPAR-γ to facilitate their deubiquitination, thereby removing ubiquitin tags and preventing proteasomal degradation. Consequently, the stabilized transcription factors SREBP1/2 and PPAR-γ upregulate the expression of key metabolic enzymes, including *FASN*, *ACC1*, and *SCD1* (SREBP1/2 targets) [21–23], as well as *CPT1A* (PPAR-γ targets). Through this mechanism, PSMD11 promotes both fatty acid synthesis and fatty acid oxidation, ultimately driving HCC progression.

HCC is a highly prevalent and lethal malignancy worldwide. Its pathogenesis is marked by substantial etiological heterogeneity, with major risk factors including hepatitis B/C virus infection, chronic alcohol consumption, and non-alcoholic steatohepatitis (NASH)-associated metabolic dysfunction [24, 25]. HCC driven by distinct etiologies exhibits significant variations in genomic mutation profiles, tumor microenvironment characteristics, clinical prognosis, and therapeutic responses, suggesting that the etiological background profoundly influences the functional patterns of tumor driver genes. In this study, we observed a consistent upregulation of PSMD11 in HCC tissues. To investigate potential expression differences and functional heterogeneity across various HCC etiologies, we systematically re-analyzed HCC cohorts stratified by underlying causes (viral hepatitis, NAFLD/NASH, and alcohol-related). Although the sample size, particularly within certain etiological subgroups, was relatively limited, potentially affecting statistical power, our analysis revealed no significant correlation between PSMD11 expression levels and specific etiological factors (e.g., HBV vs. NASH vs. Alcohol; *p* > 0.05) (Supplementary Figs. S14, 15). These findings indicate that PSMD11 dysregulation is a ubiquitous feature of HCC, independent of the initial triggering factor (viral, metabolic, or toxic). This etiology-independent expression pattern strongly supports the potential of PSMD11 as a broad-spectrum therapeutic target for HCC patients, regardless of their specific disease background.

In cancer cells, UPS dysregulation frequently leads to aberrant stabilization of oncoproteins and anti-apoptotic factors, as well as accelerated degradation of tumor suppressors and pro-apoptotic proteins. It is well established that the expression of proteasome-related genes is commonly elevated in tumors [6, 7, 26, 27]. In this study, we also found that PSMD11 was upregulated in HCC and associate with poor patient prognosis. PSMD14 (also known as POH1), a deubiquitinating enzyme (DUB) integral to the 19S regulatory particle, is also overexpressed in HCC and has been shown to promote tumorigenesis by stabilizing key oncoproteins such as E2F1 and TGF-β receptors through deubiquitination [20, 28]. Notably, our results reveal a functional interaction between PSMD11 and PSMD14 that drives HCC progression. Based on our in vitro and in vivo experimental data, we conclude that targeting the PSMD11–PSMD14 interaction or deubiquitinase activity is biologically feasible; however, significant translational challenges remain. The UPS is fundamental to cellular homeostasis, and systemic inhibition often leads to severe adverse effects such as bone marrow suppression and neurotoxicity. Clinically, while proteasome inhibitors like bortezomib have become a cornerstone of multiple myeloma treatment by inducing proteotoxic stress and apoptosis, their utility is frequently limited by dose-limiting toxicities, including peripheral neuropathy, thrombocytopenia, and gastrointestinal complications [29]. Although solid tumors have shown more limited responses, preclinical evidence suggests context-specific vulnerabilities. For instance, in intrahepatic cholangiocarcinoma (ICC), PTEN deficiency has been associated with increased sensitivity to bortezomib [30]. Although combinatorial regimens involving proteasome inhibitors have shown enhanced efficacy [31, 32], there is a critical need for more selective approaches. To overcome these limitations, there is a critical need for more selective therapeutic approaches. Targeting the specific interface between PSMD11 and PSMD14, or modulating complex-associated deubiquitinase activities, presents a compelling solution. Unlike broad-spectrum inhibitors, this targeted strategy aims to selectively disrupt the turnover of key oncogenic substrates while preserving essential global proteasome function, thereby offering a potentially superior safety profile. Consequently, the development of small molecules or stapled peptides designed to block the PSMD11–PSMD14 interaction represents a technically feasible and high-priority objective.

In cancer metabolism, dysregulated FAO is increasingly recognized as a key metabolic adaptation [33]. Solid tumor cells often rely on exogenous fatty acids as a critical energy and biosynthetic substrate, particularly under metabolic stress conditions such as nutrient deprivation or hypoxia. This metabolic flexibility supports tumor survival, growth, and resistance to therapy [34, 35]. Notably, elevated FAO has been shown to accelerate hepatocarcinogenesis in certain contexts [35]. CPT1A is the predominant isoform catalyzing the rate-limiting step of FAO, particularly under conditions of metabolic stress or high energy demand [36]. In non-steatotic HCC cases, CPT1A expression is downregulated in tumor tissues compared to adjacent non-tumor liver; notably, low CPT1A levels are negatively correlated with patient prognosis [37]. Similarly, reduced CPT1A expression is associated with TP53 mutations and poor clinical outcomes in HCC [38]. In contrast, during the progression from non-alcoholic steatohepatitis (NASH) to HCC, CPT1A is upregulated and promotes oncogenic signaling pathways and driving liver cancer cell proliferation [39, 40]. This dichotomy likely stems from divergent sources of fatty acids. In non-steatotic HCC cells, de novo synthesized fatty acids are primarily directed toward phospholipid synthesis (e.g., phosphatidylcholine and phosphatidylethanolamine), facilitating the generation of new membranes essential for rapid cell proliferation [41]. By contrast, HCC patients with metabolic syndrome display an overload of oxidizable fatty acids, thereby driving a preference for acute fatty acid oxidation to meet energy demands [42]. Consistent with this, we observed that PSMD11 promotes lipid droplet accumulation in HCC cells. Moreover, previous studies have demonstrated that a high-fat microenvironment enhances fatty acid β-oxidation, contributing to the aggressive progression of steatotic HCC [39]. Together, these findings support a context-dependent role for FAO in liver cancer, in which enhanced β-oxidation promotes HCC development, particularly in the setting of hepatic steatosis and lipid abundance.

Increased FAO drives the activation of OXPHOS to meet the energy demands of cancer cells [43]. In this study, we observed that mitochondrial activity was significantly higher in PSMD11-overexpressing HCC cells compared to control cells. Furthermore, we demonstrated that inhibition of FAO, either pharmacologically or through treatment with a CPT1A inhibitor, effectively abrogated the enhanced mitochondrial OXPHOS induced by PSMD11 overexpression. These findings suggest that the PSMD11-FAO-OXPHOS axis is a key metabolic dependency in HCC. Consequently, targeting this pathway via proteasome or FAO inhibitors represents a promising therapeutic strategy for HCC, particularly in tumors with elevated PSMD11 expression.

The PPAR signaling pathway plays a critical role in regulating fatty acid oxidation and lipogenesis [44]. In this study, we found that PSMD11 stabilizes PPAR-γ by reducing its ubiquitination, leading to increased PPAR-γ protein levels. As a transcription factor, PPAR-γ directly binds to the CPT1A promoter to activate its transcription. While previous studies have shown that HIF1α and PPARα can also regulate CPT1A expression through direct promoter binding [45, 46], our findings identify PPAR-γ as a novel transcriptional activator of CPT1A in HCC, revealing an additional layer of regulatory control within the PPAR signaling network.

In conclusion, we present evidence highlighting the clinical importance of increased PSMD11 levels in HCC. Upregulation of PSMD11 promotes HCC progression by reprogramming fatty acid (FA) metabolism. We have elucidated a novel mechanistic pathway in which PSMD11 stabilizes the PPAR-γ protein, leading to enhanced transcriptional activation of CPT1A, a key regulator of fatty acid oxidation. Together, our findings suggest that PSMD11 and may serve as prognostic biomarkers and therapeutic targets in HCC. Importantly, targeting fatty acid metabolism may represent a promising therapeutic strategy, particularly for HCC patients with high PSMD11 expression who exhibit increased dependence on lipid-derived energy sources.

## Materials and methods

### Cell lines and cell culture

Huh7 cell lines was purchased from Riken Cell Bank (Tsukuba, Japan). Hep3B and HEK-293T cell lines were obtained from the American Type Culture Collection (Manassas, VA, USA). MHCC-LM3 and MHCC-97H cell lines were provided by the Liver Cancer Institute, Zhongshan Hospital of Fudan University (Shanghai, China). All cell lines were maintained in Dulbecco’s Modified Eagle’s Medium (DMEM; Gibco) supplemented with 10% heat-inactivated fetal bovine serum (FBS; Gibco), and cultured at 37 °C in a humidified incubator containing 5% CO₂. Cell lines were used within six months of resuscitation to minimize genetic drift. Authentication was performed by the respective suppliers using short tandem repeat (STR) profiling.

### Lentivirus production and cell transduction

Lentiviral overexpression plasmids for PSMD11, PSMD14, PPAR-γ, and CPT1A, as well as corresponding short hairpin RNA (shRNA) plasmids, were obtained from the CCSB-Broad Lentiviral Expression Library and the Human TRC shRNA Library (The RNAi Consortium). The plasmid was sequenced from the 5′ and 3′ ends to confirm its sequence. The target sequences are listed in Supplementary Table S1.

Viral particles were produced by transient transfection of HEK-293T cells using Lipofectamine 2000 (Invitrogen) according to the manufacturer’s protocol [47]. For packaging, cells were co-transfected with the lentiviral transfer plasmid, the packaging plasmid psPAX2, and the envelope plasmid pMD2.G (both obtained from Addgene). Viral supernatants were collected 48 hours post-transfection, and either used immediately or aliquoted and stored at −80 °C. HCC cells were transduced with 1 × 10^6^ recombinant lentivirus-transducing units in the presence of 6 μg/ml polybrene (Sigma, USA).

### Quantitative real-time RT‒PCR (qRT‒PCR)

Total RNA extraction and reverse transcription were performed as previously described [48]. qRT‒PCR analyses were conducted using an ABI Prism 7500 System (Applied Biosystems, Carlsbad, CA, USA) with SYBR® Premix Ex Taq (Takara, Dalian, China). Relative mRNA expression levels were normalized to housekeeping gene GAPDH. The sequences of all primers used in this study are listed in Supplementary Table S2.

### Western blotting

Total protein was extracted using a RIPA lysis buffer supplemented with Protease and phosphatase inhibitor cocktail. The protein concentrations were quantified using a BCA Protein Assay Kit in accordance with the manufacturer’s instructions [49]. Equal amounts of protein from whole-cell lysates were separated by SDS‒polyacrylamide gel electrophoresis and transferred onto PVDF membranes (Millipore, USA). Membranes were blocked for 2 h at room temperature with 5% non-fat milk in Tris-buffered saline containing 0.1% Tween 20 (TBST). Following blocking, the membrane was incubated overnight at 4 °C with primary antibodies, followed by incubation with horseradish peroxidase (HRP)-conjugated secondary antibodies for 1 h at room temperature. The immune complex was visualized using an enhanced chemiluminescence reagent (Pierce, Rockford, IL, USA). β-Actin was used as a loading control. Detailed information on the antibodies used is provided in Supplementary Table S3.

### Cell proliferation and colony formation assays

Cell proliferation was measured by the Cell Counting Kit-8 (CCK-8; Bimake, USA) according to the manufacturer’s instructions [50]. Briefly, 5000 cells were seeded into each well of 96-well plates and cultured in a complete medium. CCK-8 reagent was added to each well and the absorbance was measured at 450 nm using a microplate reader.

Cell proliferation was further evaluated using an EdU Incorporation Assay Kit (Abbkine, Wuhan, China) following the manufacturer’s protocol [7]. After EdU staining, cells were visualized and imaged using a fluorescence microscope.

For colony formation assays, HCC cells were seeded into 6-well plates at a density of 1000 cells per well and incubated at 37 °C for 2 weeks. Colonies were fixed with 4% phosphate-buffered formalin (pH 7.4) for 10 min, followed by staining with Giemsa solution for 15 min. Each experiment was performed independently in triplicate to ensure reproducibility.

### Migration and invasion assays

Cell migration and invasion assays were performed using Transwell inserts with 8-μm pore size or Matrigel-coated inserts (BD Biosciences, NJ) for invasion, according to the manufacturer’s instructions [51]. Briefly, cells were seeded in the upper chamber in serum-free media. The lower chamber contained DMEM supplemented with 10% FBS as a chemoattractant. After incubating for 24 or 48 h, the non-migrated or non-invaded cells were gently removed from the upper chamber. The migrated or invaded cells were fixed with 4% formalin for 15 min and stained with Giemsa solution for 20 min. The number of cells in five randomly chosen fields of view was counted under a microscope.

### Flow cytometry analysis

For cell cycle analysis, cells were washed twice with cold PBS, fixed in 70% cold ethanol and incubated overnight at 4 °C. Prior to analysis, cells were centrifuged, washed, and resuspended in a staining solution containing 100 μg/mL RNase A and 40 μg/mL propidium iodide (PI) in PBS. Samples were incubated for 30 min at 37 °C in the dark. Cell cycle distribution were analyzed by flow cytometry and the data were analyzed using FlowJo software.

For the apoptosis assay, cells were harvested, washed with cold PBS, and stained with PE-conjugated Annexin V and 7-AAD for 15 min at room temperature according to the manufacturer’s instructions. Stained cells were immediately analyzed by flow cytometry, and data were analyzed using FlowJo software. All results are representative of three independent experiments performed in triplicate.

### Immunofluorescence analysis via confocal imaging

Immunofluorescence analysis was performed according to the manufacturer’s instructions [52]. Briefly, cells were seeded on Lab-Tek chamber slides (Nunc), fixed with 4% paraformaldehyde in PBS for 30 min, and permeabilized with 0.1% Triton X-100 in PBS for 15 min. Primary antibodies were diluted in blocking solution and applied to the slides, which were then incubated overnight at 4 °C in a humidified chamber. After three washes with PBS, the slides were incubated with Alexa Fluor 488-conjugated and Alexa Fluor 555-conjugated secondary antibodies and 4′, 6- diamidino-2-phenylindole (DAPI) in blocking solution for 30 min at 37 °C in the dark. Fluorescence images were acquired using a Leica TCS SP8 confocal laser scanning microscope (Leica Microsystems, Germany). Antibody details are provided in Supplementary Table S3.

### Oxygen consumption rate (OCR) determination

OCR was measured with a Seahorse XFe 96 Extracellular Flux Analyzer according to the manufacturer’s instructions [53]. Briefly, cells were seeded in an XFe 96 plate with complete DMEM medium for 24 h. One hour prior to analysis, the medium was changed to Seahorse XF Base Medium. OCR was assessed by sequentially adding the following compounds: oligomycin (1.5 μmol/L), FCCP (1 μmol/L), and rotenone and antimycin A (each 0.5 μmol/L). The results were normalized to the number of cells in each plate determined at the time of measurement.

### Coimmunoprecipitation (Co-IP) assay

Co-IP assay was conducted as reported previously [54]. Cells were lysed on ice for 40 min in RIPA lysis buffer (Millipore) supplemented with freshly added protease inhibitors. Lysates were cleared by centrifugation at 12,000 × *g* for 10 min at 4 °C, and the supernatants were collected for immunoprecipitation. Protein A/G agarose beads were pre-incubated with either anti-PSMD11 antibody or normal IgG overnight at 4 °C. The cell lysates were then added to the antibody-bound beads and incubated for overnight with gentle rotation. Immune complexes were washed five times with ice-cold lysis buffer to reduce non-specific binding, eluted by boiling in SDS-PAGE sample buffer, and subjected to Western blot analysis.

### Luciferase assay

The CPT1A promoter region (–945 to +16 bp relative to the ATG start codon) was amplified by PCR and cloned into the pGL3-Enhancer (Promega) to generate the pGL3-CPT1A-promoter construct. Three 5’-truncated sequences of CPT1A promoter were generated by PCR and cloned and inserted into the luciferase reporter gene vector pGL3-Enhancer. The fidelity of the constructs was confirmed by sequencing. Primer sequences used for cloning are provided in Supplementary Table S2. Luciferase assay was conducted as reported previously [55]. Briefly, cells were seeded in 48-well plates and co-transfected with the pGL3-CPT1A-promoter plasmid and the indicated expression or control plasmids using Lipofectamine 2000. To normalize for transfection efficiency, the pRL-TK Renilla luciferase plasmid (Promega) was included in each transfection as an internal control. After 48 h of incubation, luciferase activity was measured using the Dual-Luciferase Reporter Assay System (Promega) according to the manufacturer’s instructions. All experiments were performed in triplicate and repeated independently at least three times.

### Chromatin immunoprecipitation assay

Chromatin immunoprecipitation (ChIP) assays were performed using a ChIP Assay Kit according to the manufacturer’s instructions and as previously described [55]. Briefly, HCC cells were cultured to 90% confluence, and crosslinked with 1% formaldehyde for 10 min at room temperature. Cell lysates were sonicated to shear the DNA to fragments of 500 to 1000 bp. A rabbit anti-PPAR-γ antibody or rabbit IgG was used to immunoprecipitate DNA-containing complexes. Protein–DNA crosslinks were reversed by heating at 65 °C, followed by proteinase K treatment to purify DNA. The enrichment of PPAR-γ binding at the CPT1A promoter region was quantified by qRT-PCR using primers listed in Supplementary Table S2.

### In vivo growth assays

To assess the in vivo growth of HCC cells, 6-week-old male BALB/c nude mice were injected subcutaneously with 2 × 10^6^ cells suspended in 100 μL of PBS into the right flank. Tumor growth was monitored every three days using a Vernier caliper, and tumor volume was calculated using the formula: tumor volume = (length × width^2^)/2. All animal experiments were approved by the Renji Hospital Institutional Animal Care and Use Committee (RT2022-122u) and performed in accordance with the Institutional Guide for the Care and Use of Laboratory Animals.

### Statistical analysis

All the data are presented as the means ± standard deviations (SDs). Unpaired *t* test was used to compare the means between two groups, and one-way ANOVA was used to compare the means among multiple groups. The Kaplan‒Meier method was used to plot survival curves, which were compared by the log-rank test. *p* < 0.05 was considered to indicate statistical significance.

## Supplementary information

Supplementary information

WB images

aj-checklist

## Data Availability

The data that support the findings of this study are available from the corresponding author upon reasonable request.
