# Supplementary material for: PSMD11 promotes hepatocellular carcinoma progression by reprogramming lipid metabolism via SREBP1/2 and PPAR-γ
Source: Cell Death Dis. 2026 Aug 18;17(1):925. doi: 10.1038/s41419-026-09175-1 (PMC13722701; doi:10.1038/s41419-026-09175-1)
Supplement: Supplementary file 1 [file 41419_2026_9175_MOESM1_ESM.pdf]

Supplementary FigureS1-S15

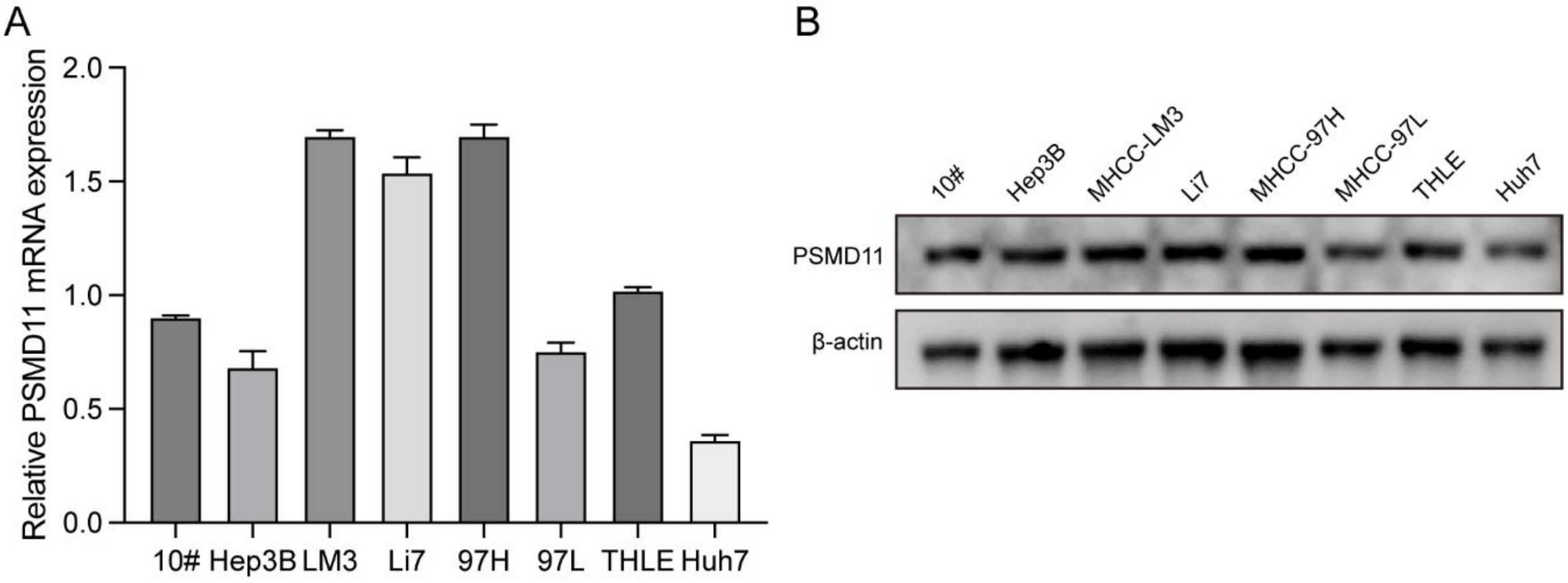

Supplementary Figure S1. The expression of PSMD11 in HCC cell lines was detected by qRT-PCR (A) and WB (B).

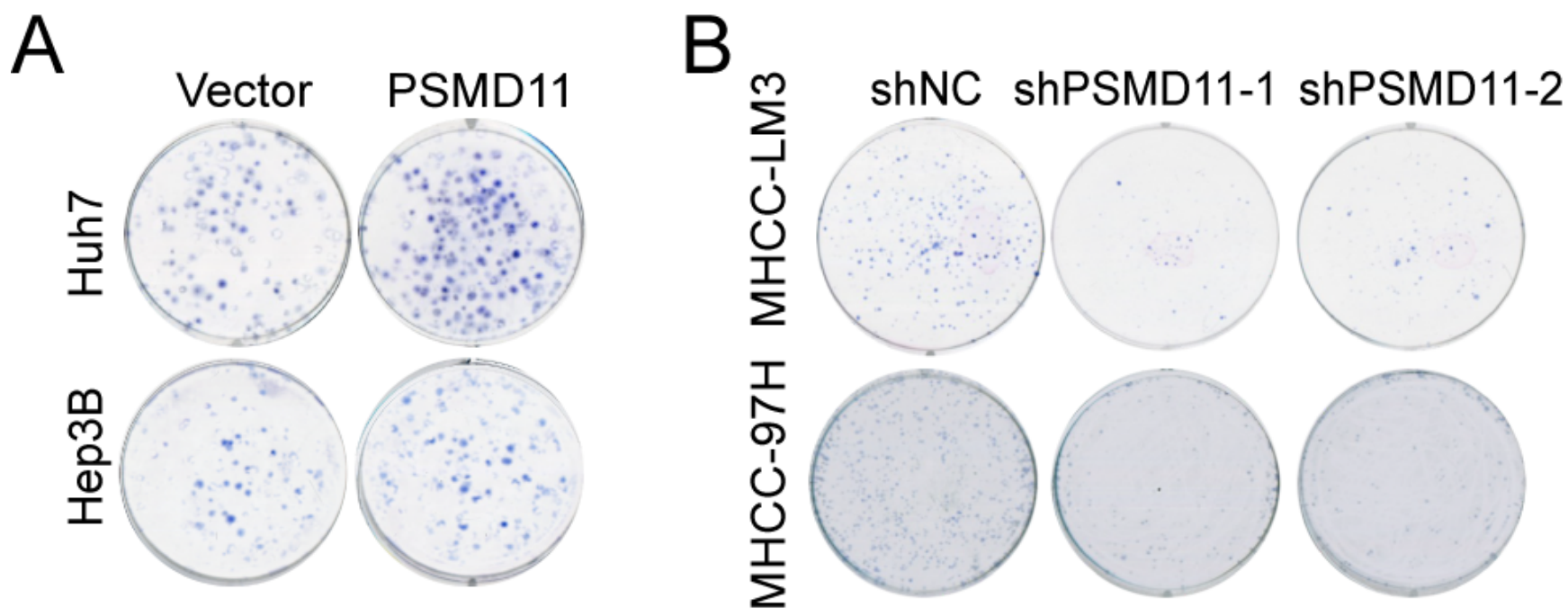

Supplementary Figure S2. Impact of PSMD11 overexpression (A) or knockdown (B) on proliferation in HCC cells, evaluated by colony formation assay.

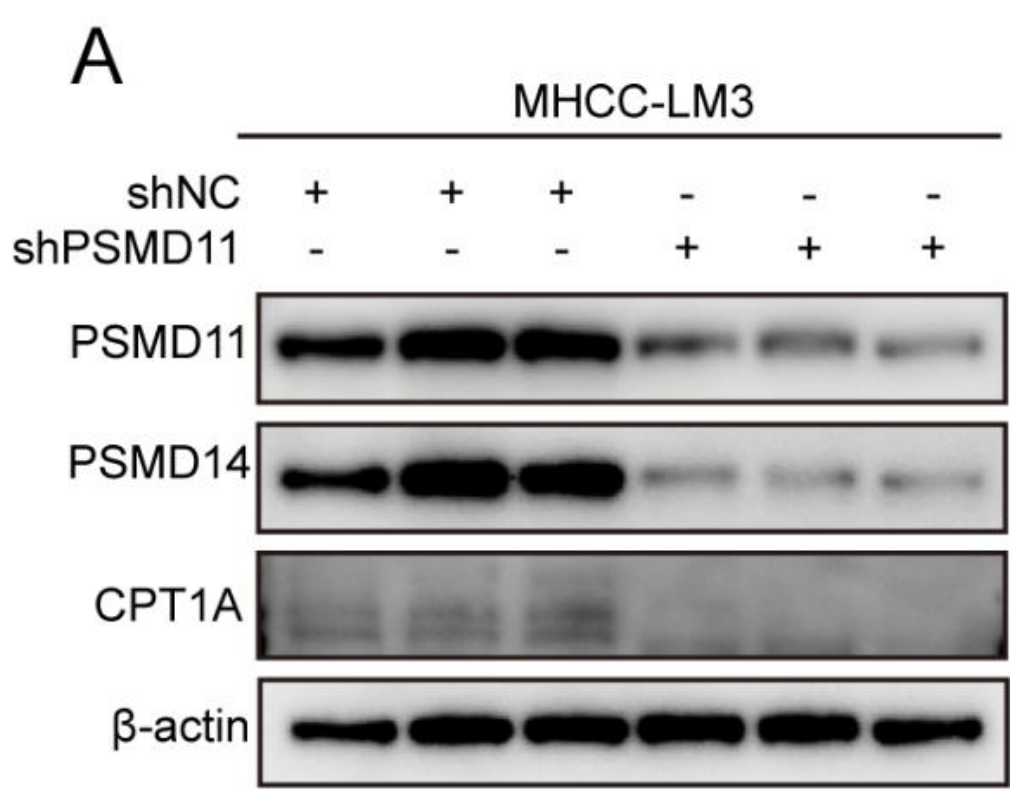

Supplementary Figure S3. The protein levels of CPT1A, PSMD14, and PSMD11 in PSMD11-knockdown MHCC-LM3 xenograft tissues were assessed by Western blotting.

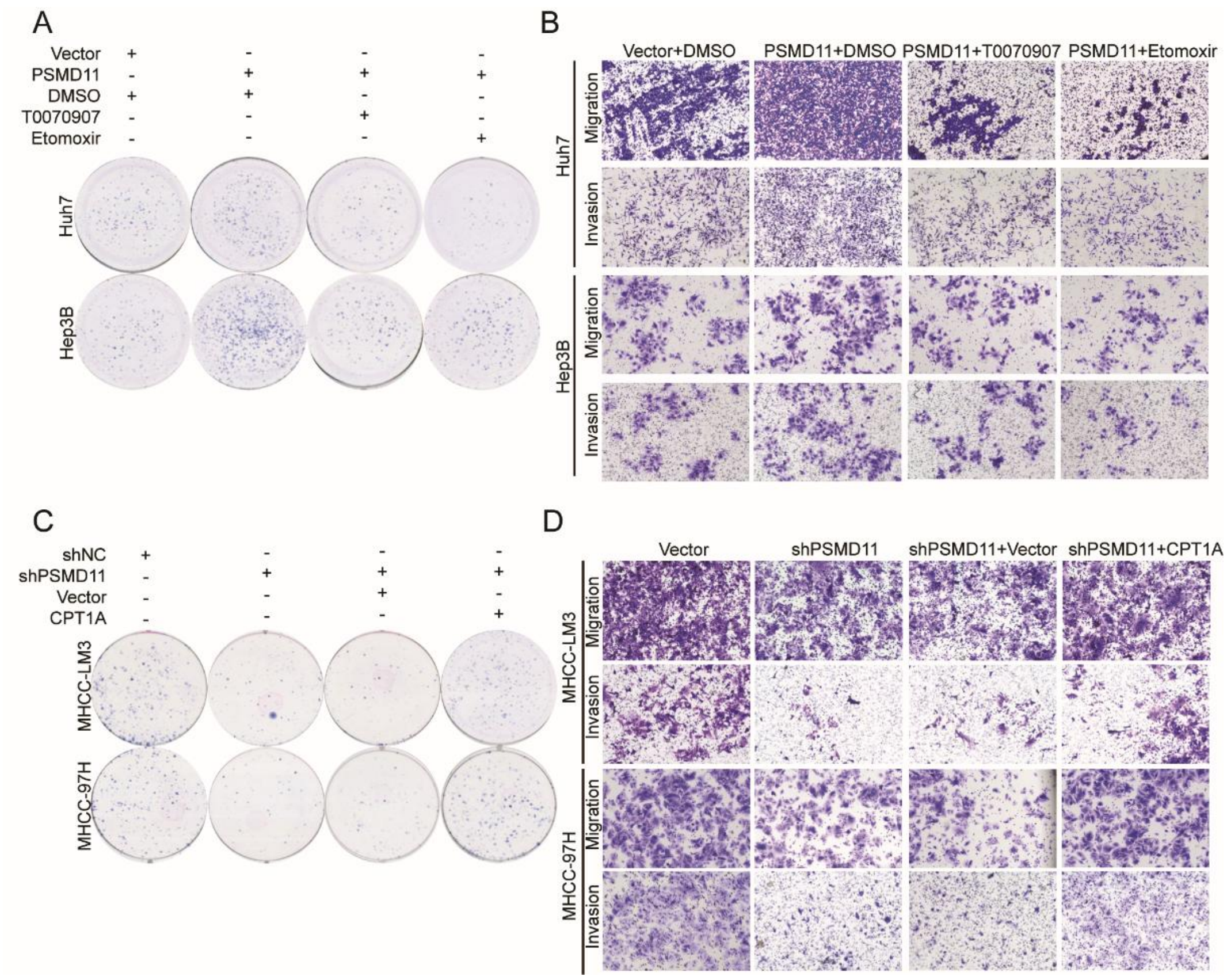

Supplementary Figure S4. PSMD11-overexpressing HCC cells were treated with etomoxir or or T0070907 or DMSO as indicated, and cell proliferation (A), migration and invasion (B) were evaluated by colony formation assay and Transwell assays. (C-D) PSMD11 knockdown HCC cells were transfected with CPT1A or vector as indicated, and cell proliferation (C), migration and invasion (D) were evaluated by colony formation assay and Transwell assays.

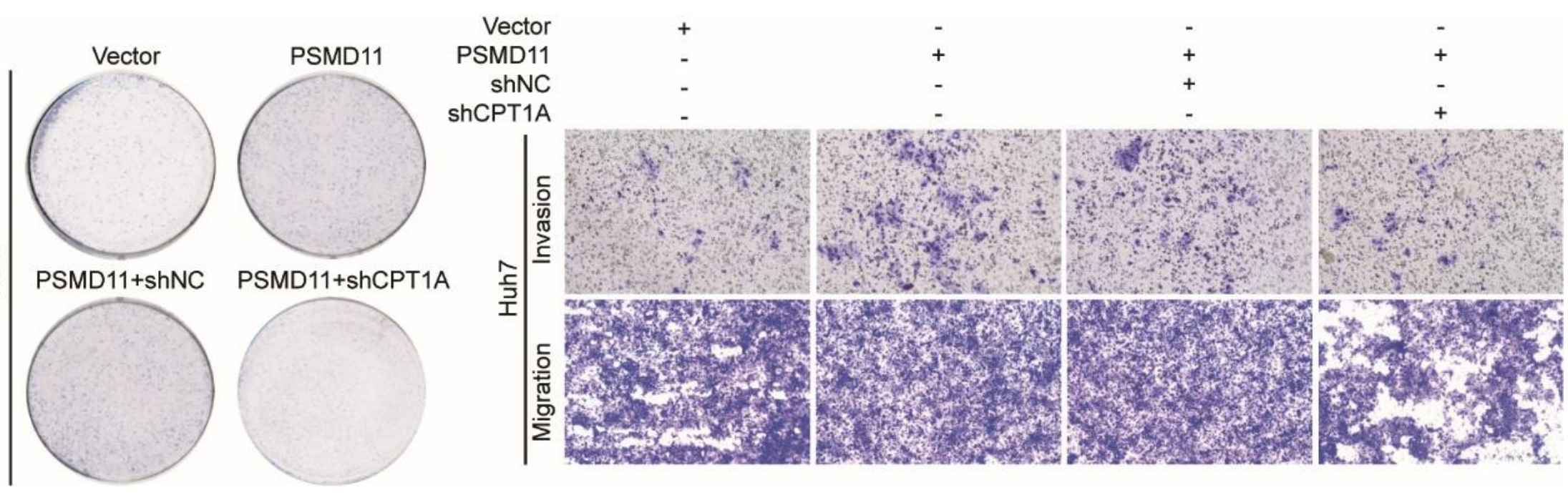

Supplementary Figure S5. PSMD11-overexpressing Huh7 cells were transfected with CPT1A shRNA or shNC as indicated, and cell proliferation, migration, and invasion were evaluated by colony formation, and Transwell assays.

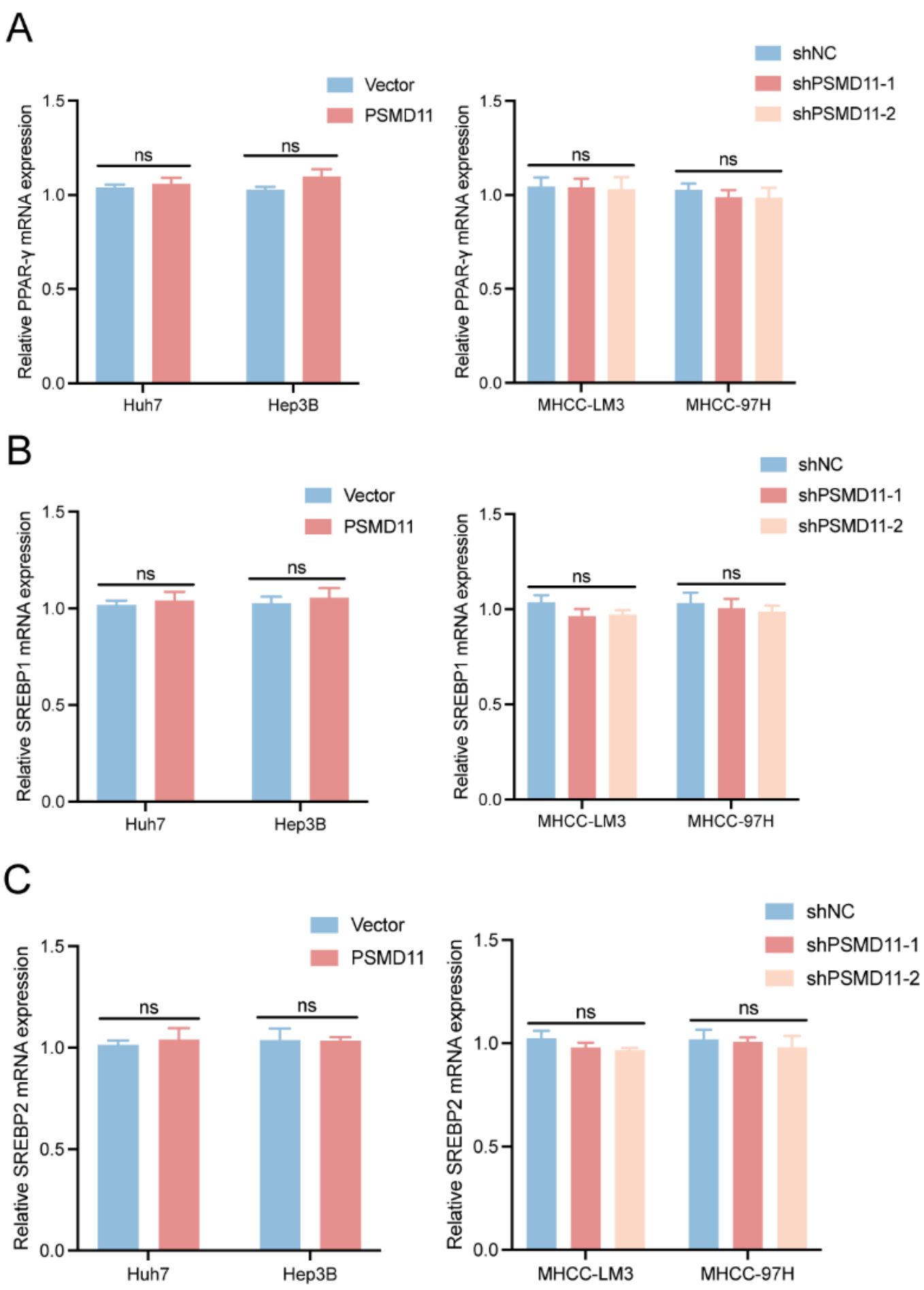

Supplementary Figure S6. The expression of SREBP1/2 and PPAR-γ was detected by qRT-PCR in PSMD11 overexpression and knockdown HCC cells.

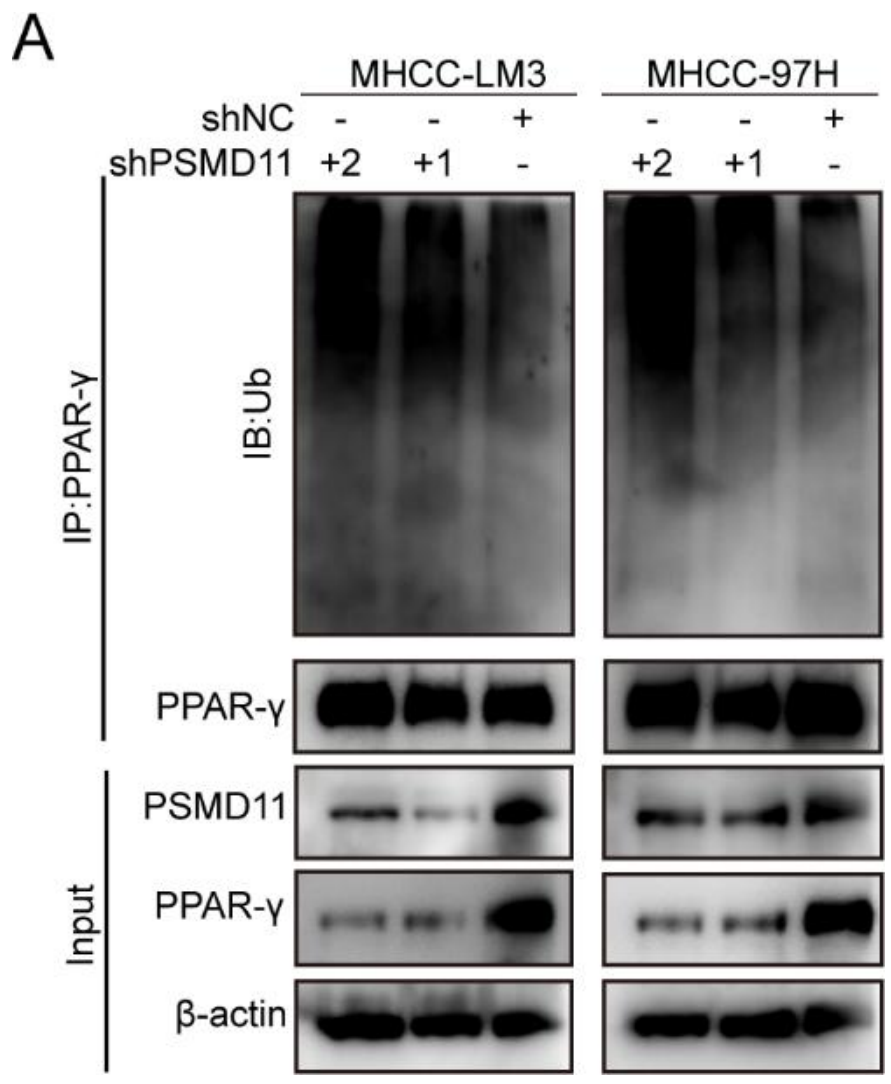

Supplementary Figure S7. Lysates of PSMD11-knockdown and control MHCC-LM3 and MHCC-97H cells were immunoprecipitated with antibody for PPAR-γ, and the immunocomplexes were immunoblotted with antibodies against ubiquitinated proteins.

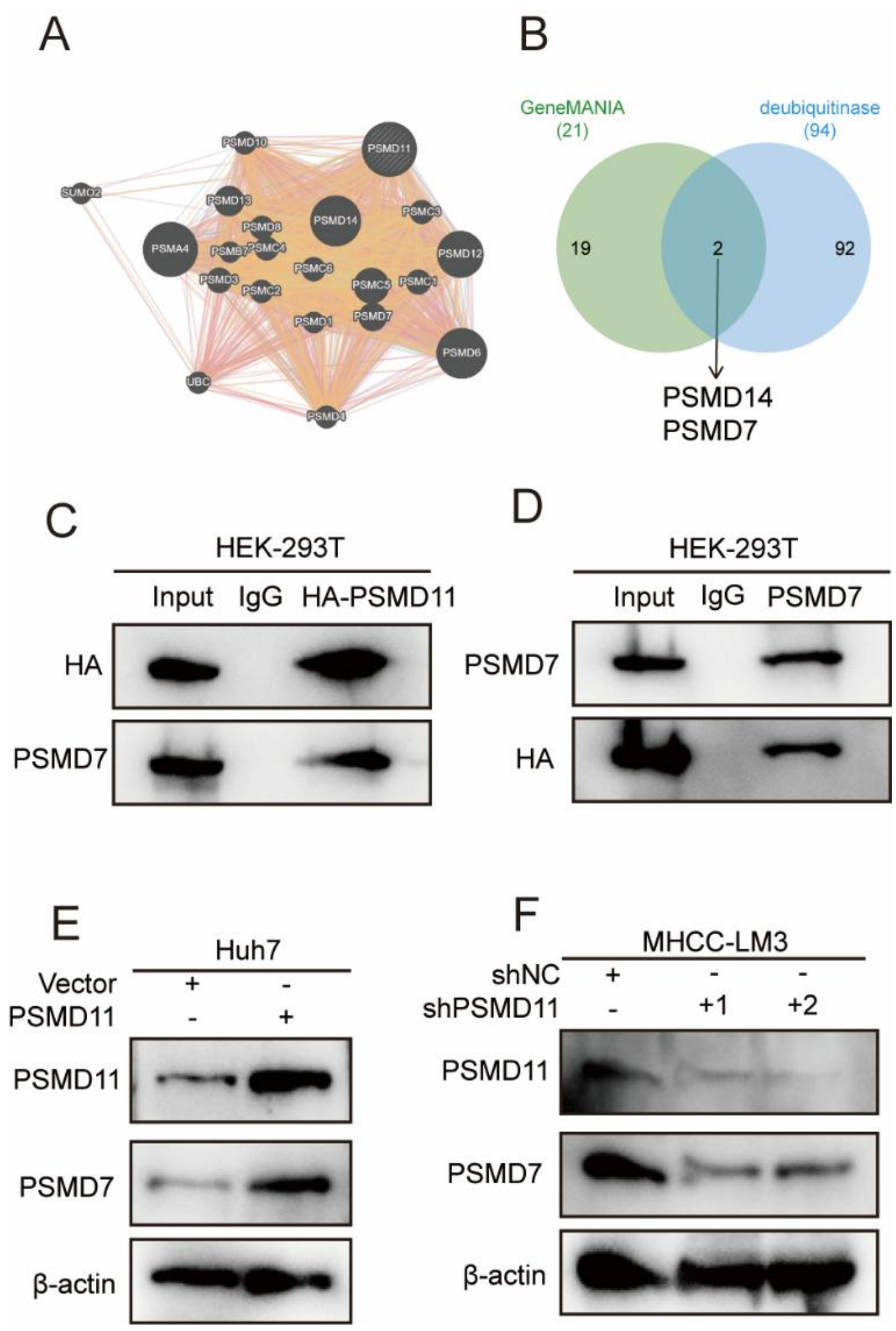

Supplementary Figure S8. (A) GeneMANIA showing PSMD11-interacting proteins. (B) Venn diagram showing the overlap between PSMD11-interacting proteins identified by GeneMANIA and deubiquitinase. (C) PSMD11 interacts with PSMD7 in HEK-293T cells using Co-IP assay. (D) The effects of PSMD11 overexpression or knockdown on the expression of PSMD7 in Huh7 and MHCC-LM3 cells.

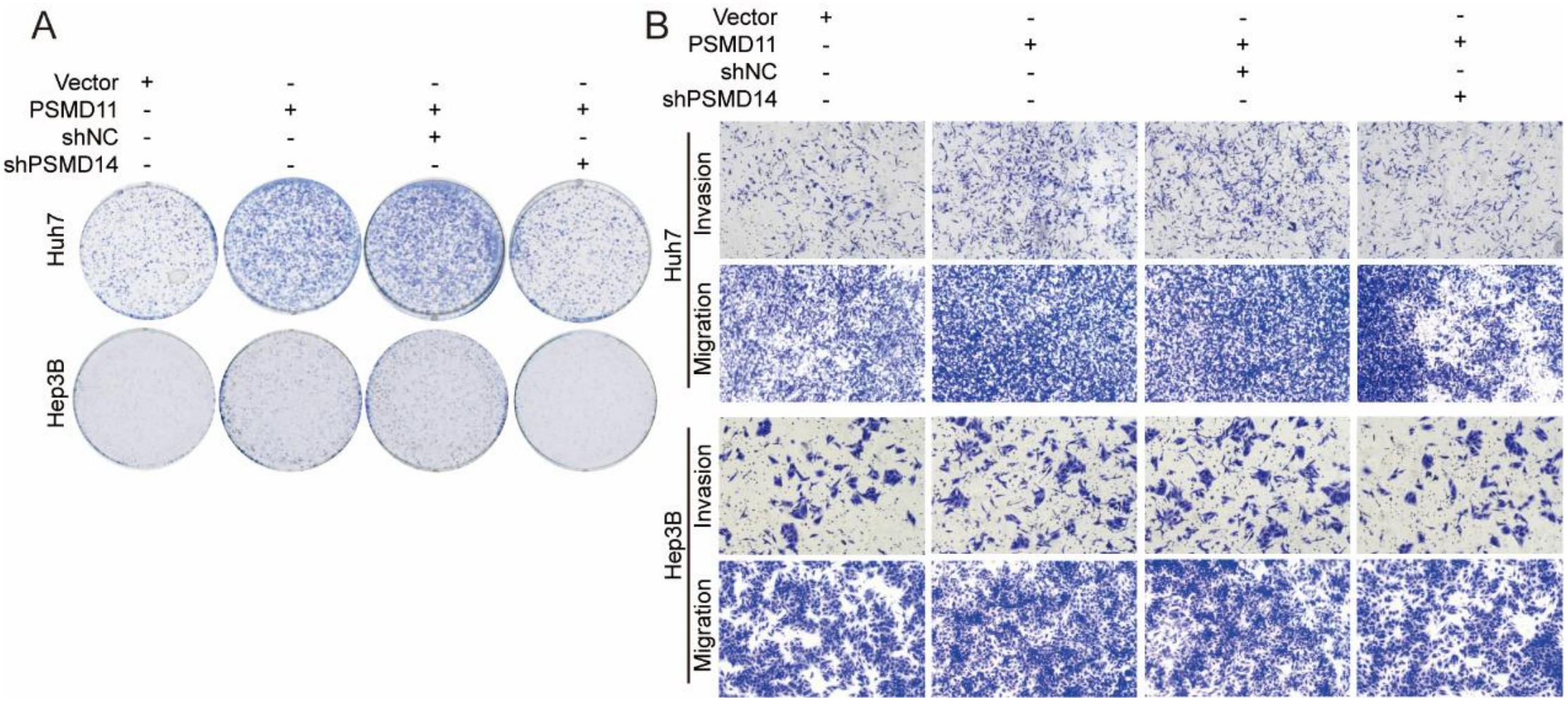

Supplementary Figure S9. (A-B) PSMD11-overexpressing HCC cells were transfected with PSMD14 shRNA or shNC as indicated, and cell proliferation, migration, and invasion were evaluated by colony formation (A), and Transwell assays (B).

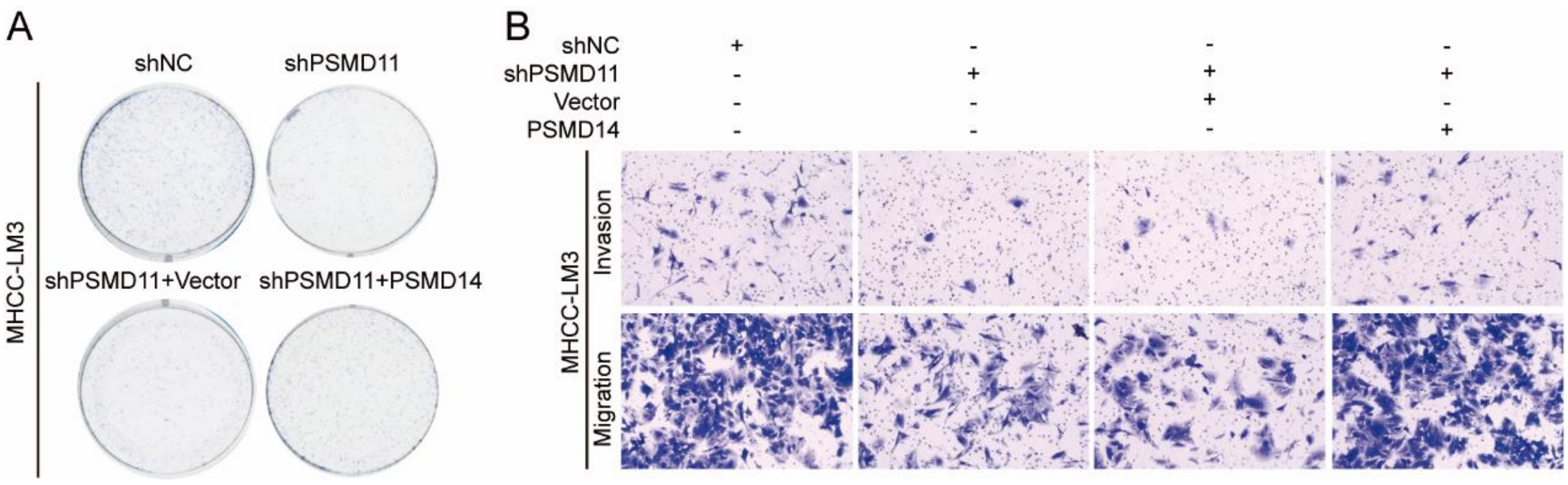

Supplementary Figure S10. PSMD11-knockdown MHCC-LM3 cells were transfected with PSMD14 or vector as indicated, and cell proliferation, migration, and invasion were evaluated by colony formation (A), and Transwell assays (B).

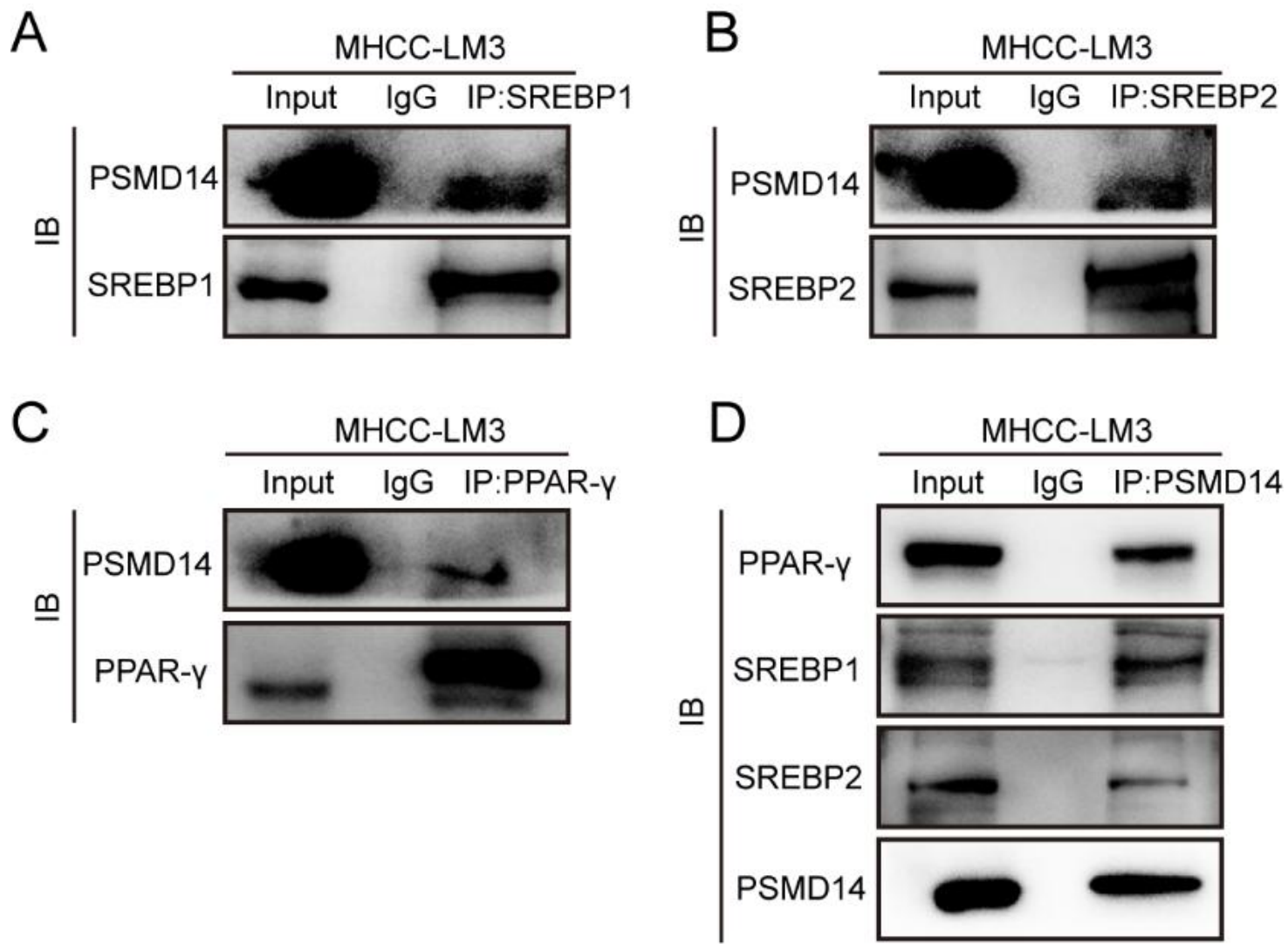

Supplementary Figure S11. PSMD14 interacts SREBP1/2 and PPAR-γ in MHCC-LM3 cells. (A–C) Co-immunoprecipitation (Co-IP) assays were performed in MHCC-LM3 cells to confirm the endogenous interaction of PSMD14 with (A) SREBP1, (B) SREBP2, and (C) PPAR-γ, using specific antibodies against the indicated proteins for immunoprecipitation. (D) Reciprocal Co-IP assays in MHCC-LM3 cells validated the binding of PSMD14 to PPAR-γ, SREBP1, and SREBP2 using an anti-PSMD14 antibody for immunoprecipitation.

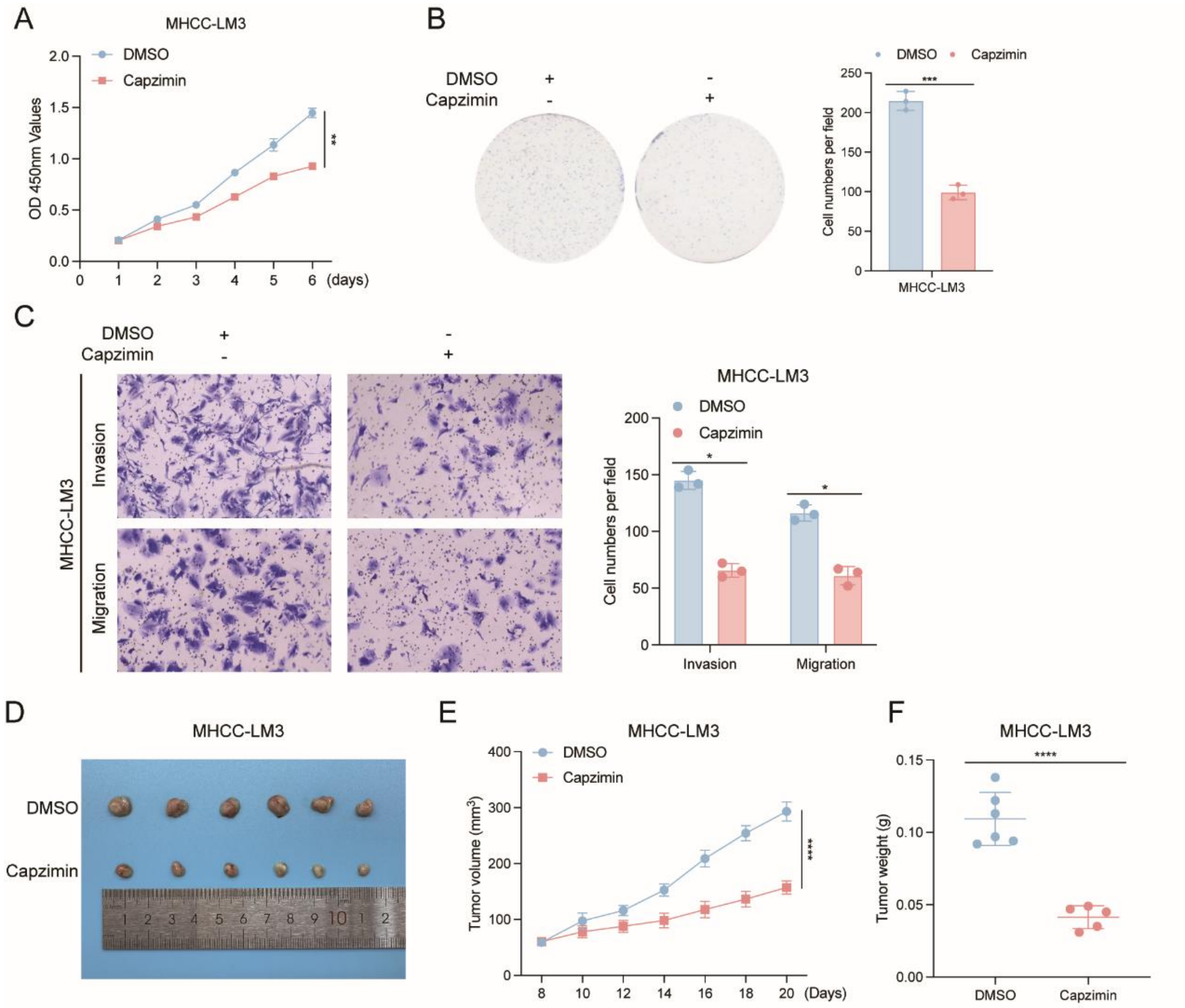

Supplementary Figure S12. Capzimin suppressed HCC cell proliferation, migration, and invasion. (A–C) Evaluation of the effects of Capzimin on proliferation, migration, and invasion in MHCC-LM3 cells. Assays include CCK-8 (A), colony formation (B), and Transwell assays (C). (D–F) *In vivo* evaluation of Capzimin on MHCC-LM3 tumor growth. Representative macroscopic images of the resected tumors are shown in (D). Tumor volumes were measured every 2 days and plotted in (E). The dot plots showed the results of the quantitative analysis of tumor weight (F). *p<0.05; **p<0.01; ***p<0.001; ****p<0.0001.

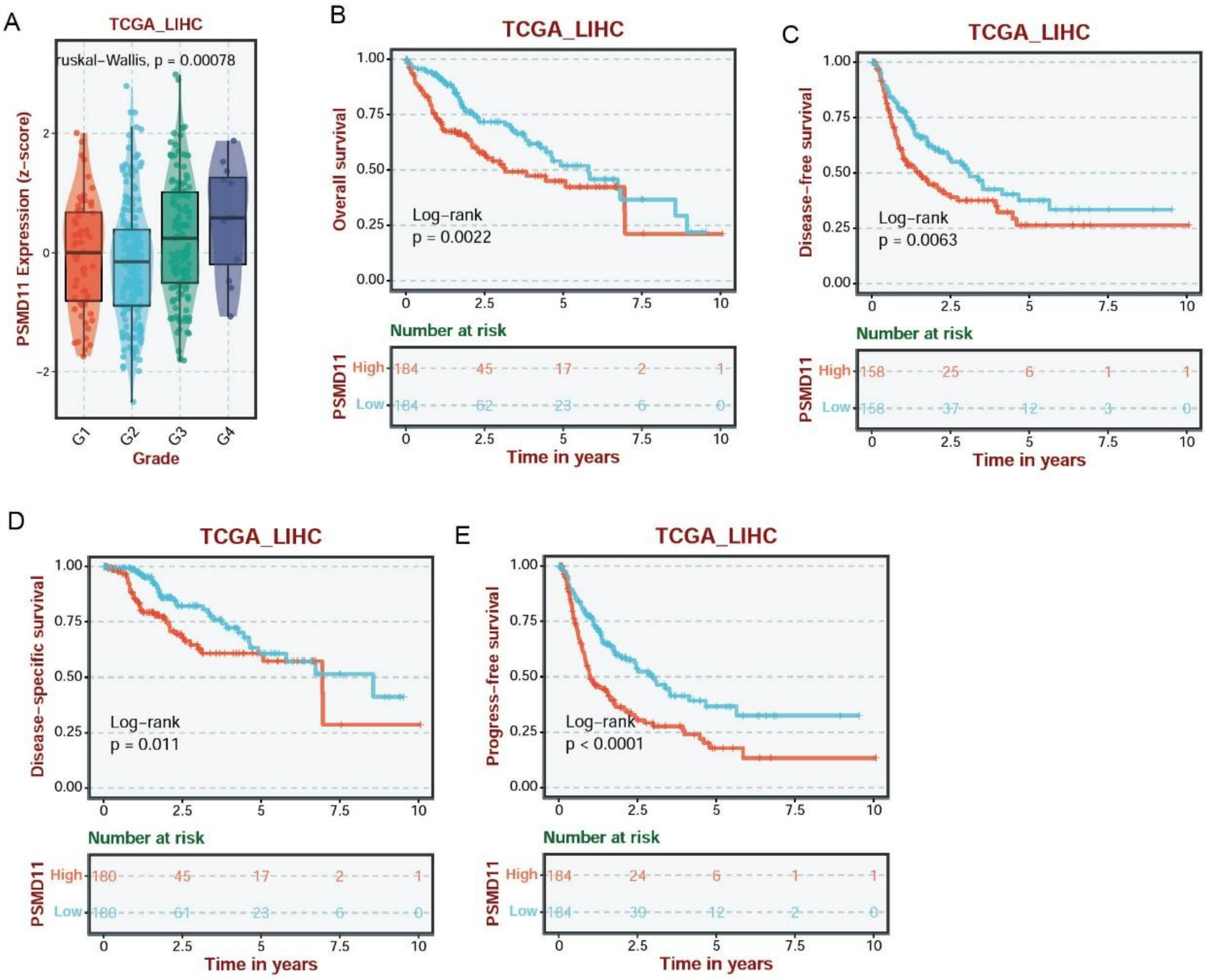

Supplementary Figure S13. Correlation analysis between PSMD11 expression in HCC patient tissues and tumor grade (A), OS (B), DFS (C), DSS (D) and PFS (E).

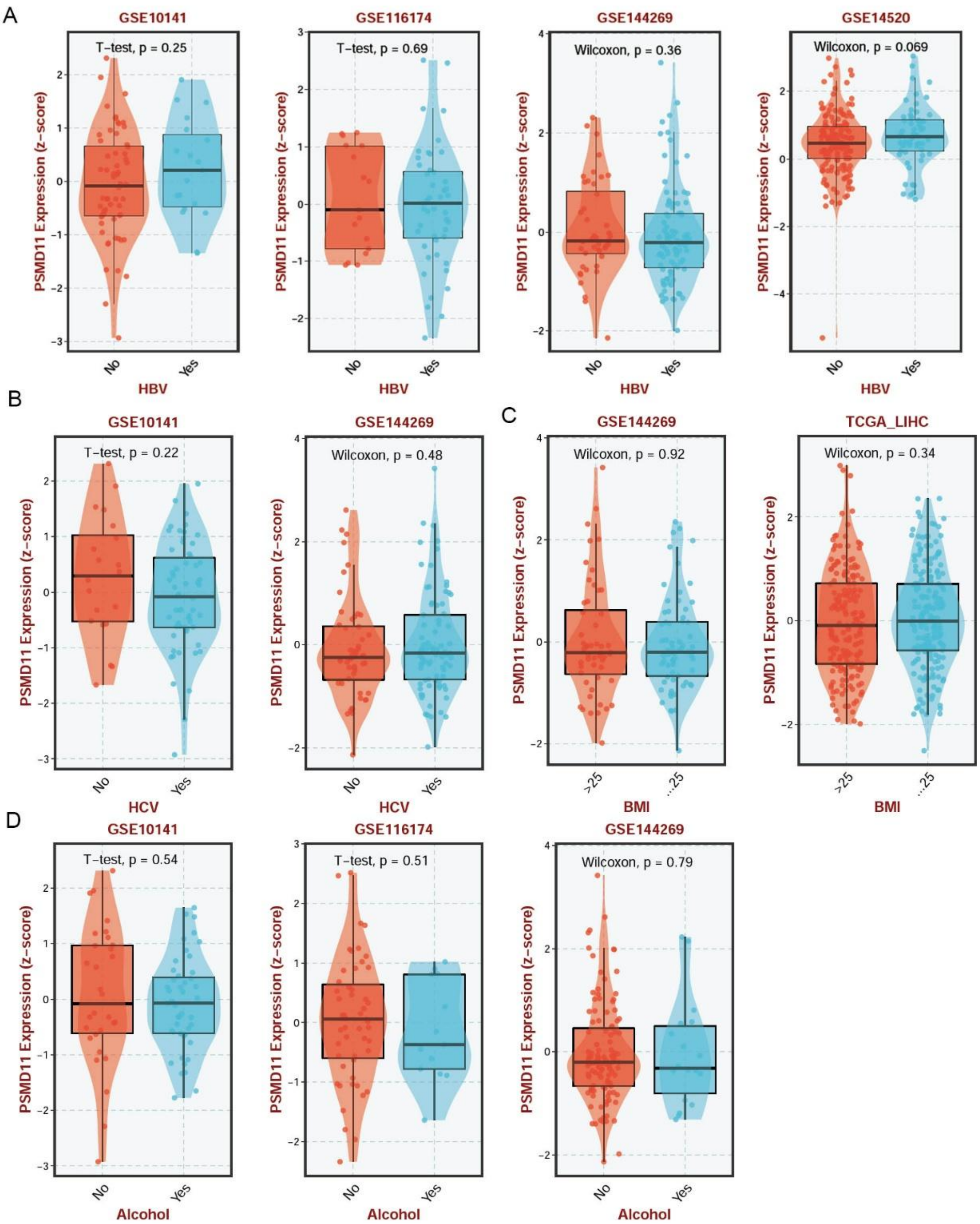

Supplementary Figure S14. Expression of PSMD11 was evaluated in HCC patients stratified by etiology: HBV (A), HCV (B), BMI (C), and alcohol consumption (D).

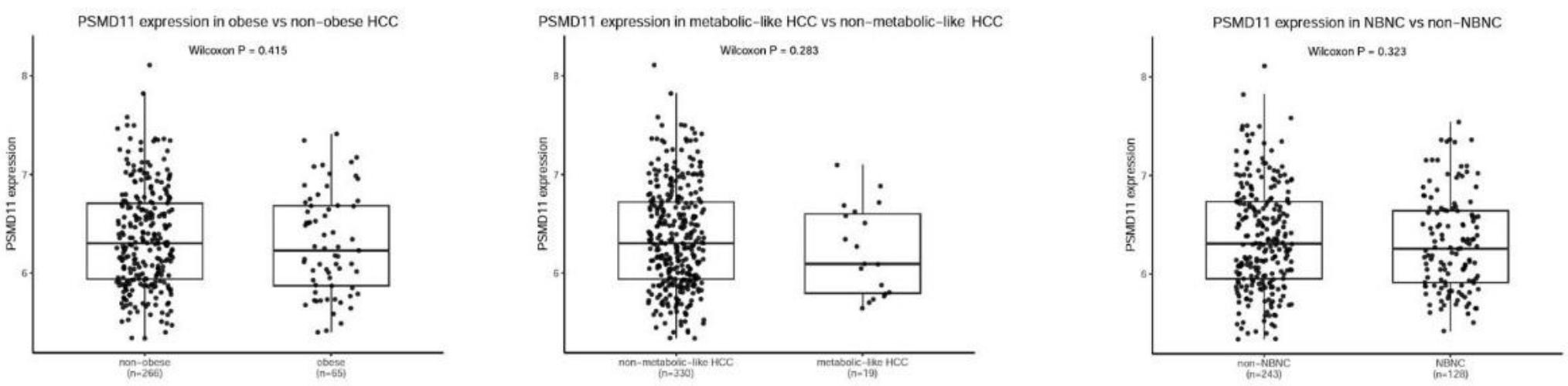

Supplementary Figure S15. Expression of PSMD11 was analyzed in metabolic syndrome-related HCC and non-metabolic syndrome-related HCC.

Supplementary Tables S1-S3:

Supplementary Table S1. The sequences of shRNA target

Identifier	Forward (5'-3')
shPSMD11-1	TGGTGTCTTTGTACTTTGAT
shPSMD11-2	CGACGTGGAAAGGAAATTAT
shCPT1A-1	CATGAAGCTCTTAGACAAA
shCPT1A-2	GTAGCCTTTGGTAAAGGAAT
shPSMD14	AGTGAACATTGTAAACACAA
shSREBP1	CCTGTGCTGACGGAAGCCAA
shSREBP2	CCTCTATTGGATGATGCAA

Supplementary Table S2. The sequences of gene-specific primers used for qRT-PCR, vector constructs and ChIP assay

Gene name	Forward (5'-3')	Reverse (5'-3')
**Primers for qRT-PCR**		
PSMD11	CTCCATCGTGAAGCGTGACA	TGCCCTGTAGCTGCTTCCAT
CPT1A	GATGGCAGAAGCTCACCAAG	CGATCTTGGCGTACATCGTT
GAPDH	AGAAGGCTGGGGCTCATTTG	AGGGGCCATCCACAGTCTT C
PPAR-γ	AGGATGCAAGGGTTTCTTCC	TCTGGATTCAGCTGGTCGAT
SREBP1	GACAGCAAGGCAAAGCCAG	GGGCTATGGTAGACGCTGGT
SREBP2	TCAAGTACCAACCCTGGTGG	ACTTGTGCATCTTGGCGTCT
**Primers for vector constructs**		
CPT1A (-945/+16)	cgACGCGTAAGGGTCTCTCGGTCGCT	cccAAGCTTGGTGAGCTTCTGCCATCTTC
CPT1A (-772/+16)	cgACGCGTCCACTCGTGGTATAAGGTCT	cccAAGCTTGGTGAGCTTCTGCCATCTTC
CPT1A (-535/+16)	cgACGCGTAACTTATTTCCTGGCCCG	cccAAGCTTGGTGAGCTTCTGCCATCTTC
CPT1A (-335/+16)	cgACGCGTTGAGGAATCTCGTCCTAGAC	cccAAGCTTGGTGAGCTTCTGCCATCTTC
CPT1A (-772/+16, Mut)	cgACGCGTCCAtTaGcGacAcAgGaTCggatttggggcatgggc	cccAAGCTTGGTGAGCTTCTGCCATCTTC
CPT1A (-535/+16,Mut)	cgACGCGTcgACGCGTAACTTATgTCCTcGtCCGctcgcattgtCTGCATGAAAGAGT	cccAAGCTTGGTGAGCTTCTGCCATCTTC
**Primers for ChIP**		
CPT1A promoter for PPARγ	CCTCGAACTCCTGGCTTCA	CTCGCGACTTATCTATCGGC

Supplementary Table S3. Antibodies used in this study

Antibody	catalog	Dilution	Company
**For Western blotting**			
PSMD11	14786-1-AP	1:500	Proteintech
Cyclin D1	SC450	1:200	Santa Cruz
p-Rb	8516S	1:200	CST
p-p53	9284	1:200	CST
PCNA	13110S	1:500	CST
PPAR-γ	2435S	1:500	CST
PSMD14	4187	1:500	CST
PSMD7	16034-1-AP	1:500	Proteintech
FASN	F0225	1:500	Selleck
HA	66006-2-1g	1:500	Proteintech
SREBP1	14088-1-AP	1:500	Proteintech
SREBP2	28212-1-AP	1:500	Proteintech
OXPHOS Kit	PK3006	1:500	Proteintech
β-actin	A3854	1:10000	Sigma
Secondary antibody	HRP conjugated goat anti-rabbit IgG	1:4000	Proteintech
Secondary antibody	HRP conjugated goat anti-mouse IgG	1:4000	Proteintech
**For Immunofluorescence staining**			
PSMD11	14786-1-AP	1:50	Proteintech
PSMD14	4187	1:50	CST
Secondary antibody	Alexa Fluor 555 anti-rabbit/mouse IgG	1:50	Invitrogen
