# Supplementary material for: PSMD11 promotes hepatocellular carcinoma progression by reprogramming lipid metabolism via SREBP1/2 and PPAR-γ
Source: Cell Death Dis. 2026 Aug 18;17(1):925. doi: 10.1038/s41419-026-09175-1 (PMC13722701; doi:10.1038/s41419-026-09175-1)

Fig1A

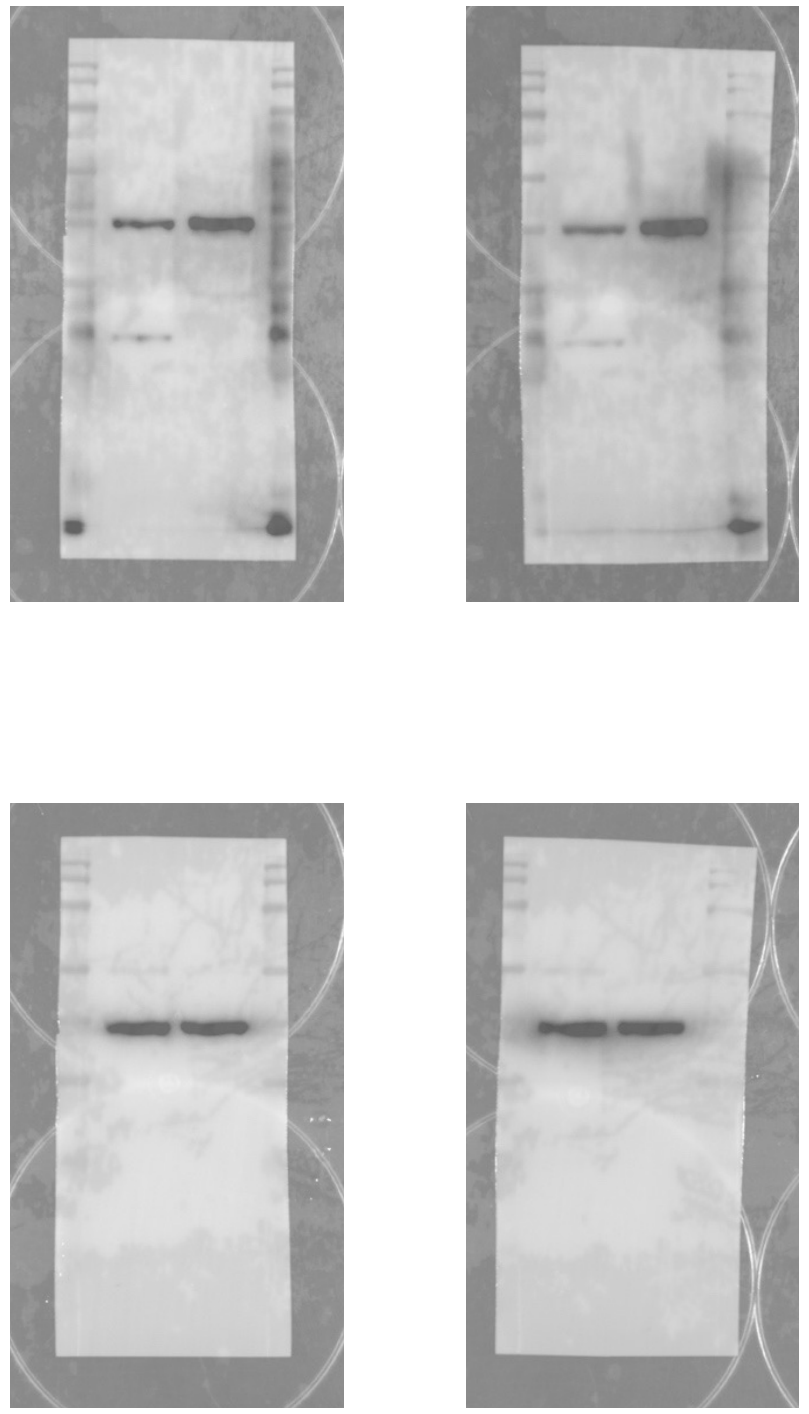

Fig1E

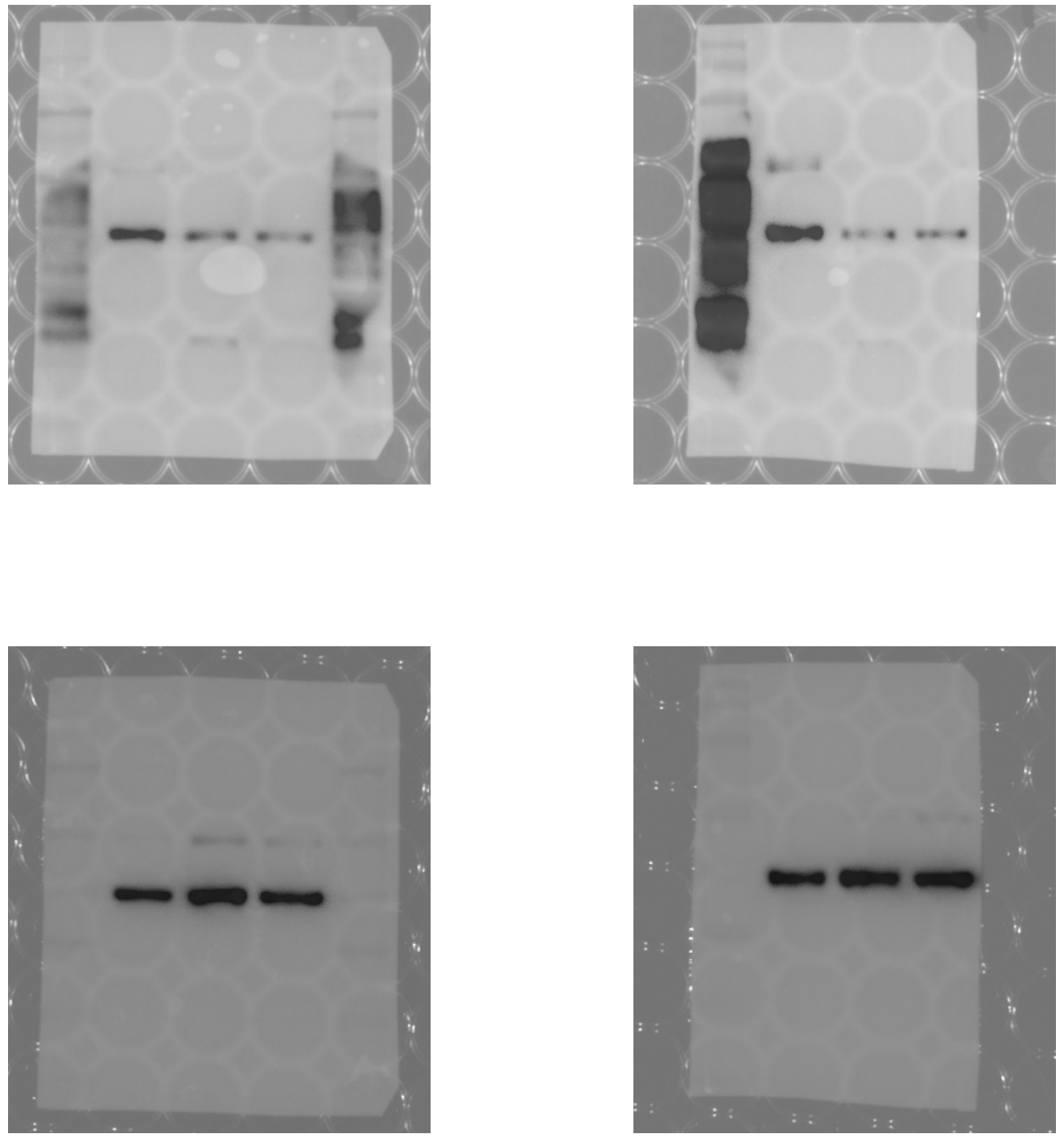

Fig2B

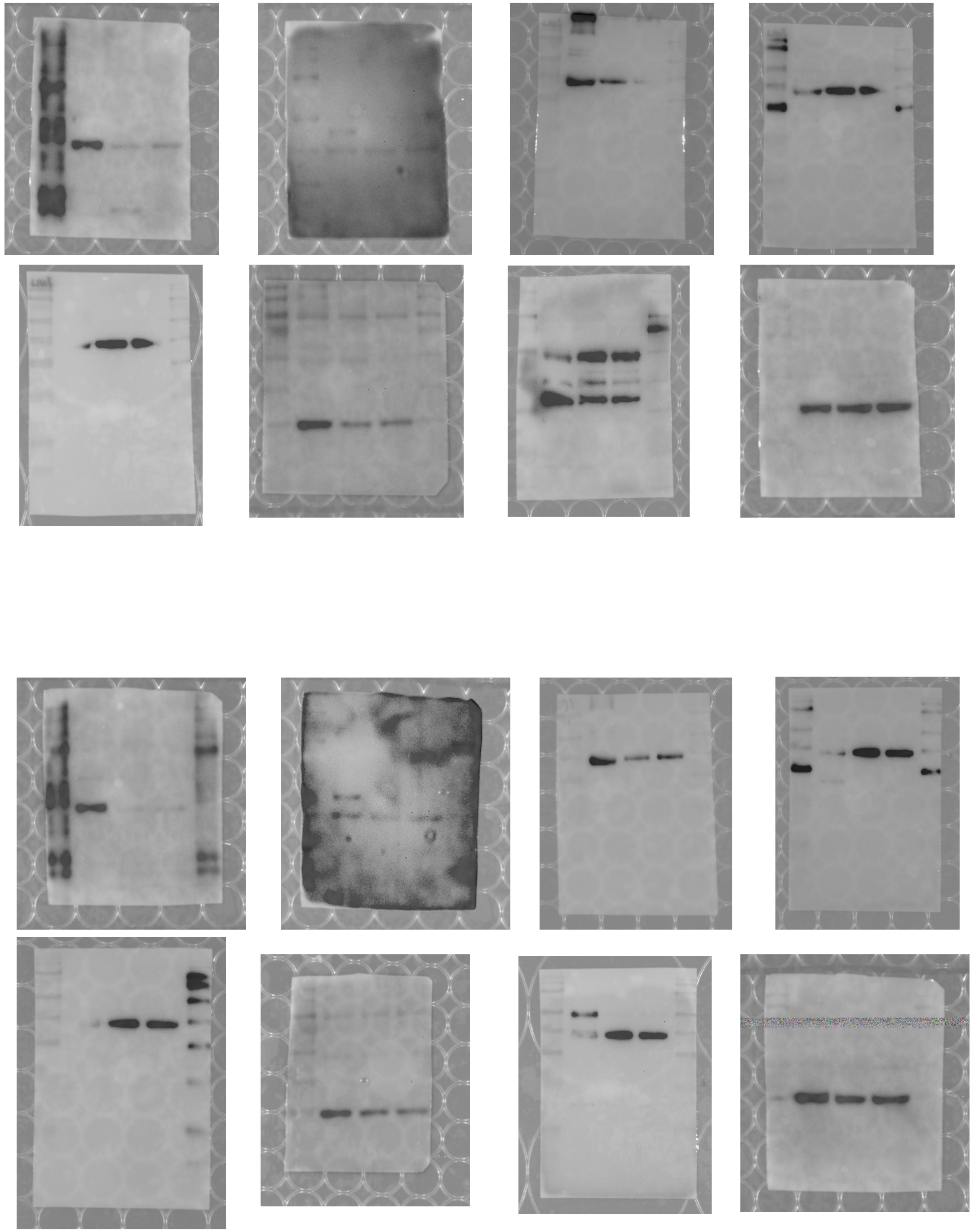

Fig2B

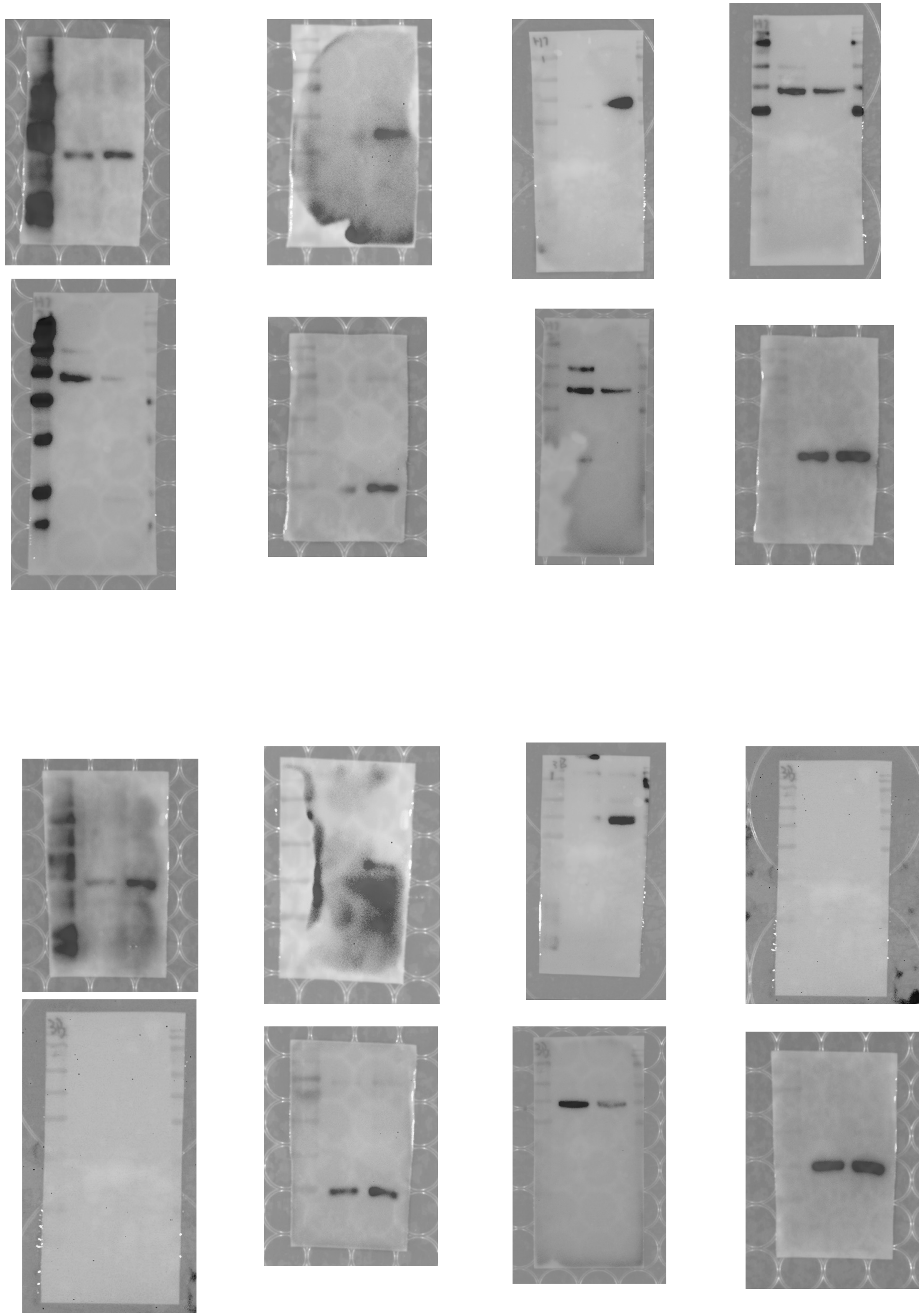

Fig2C

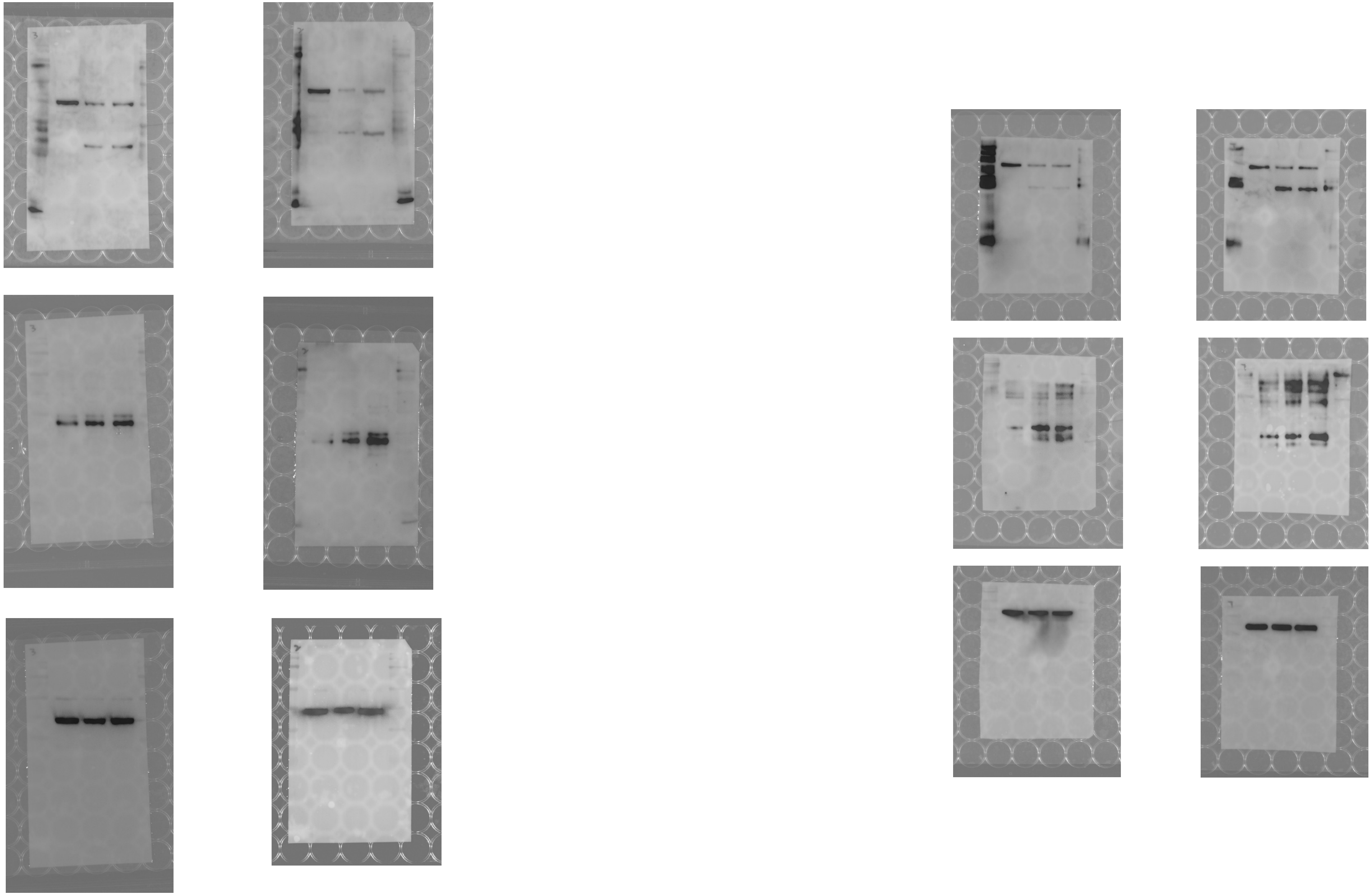

Fig3F

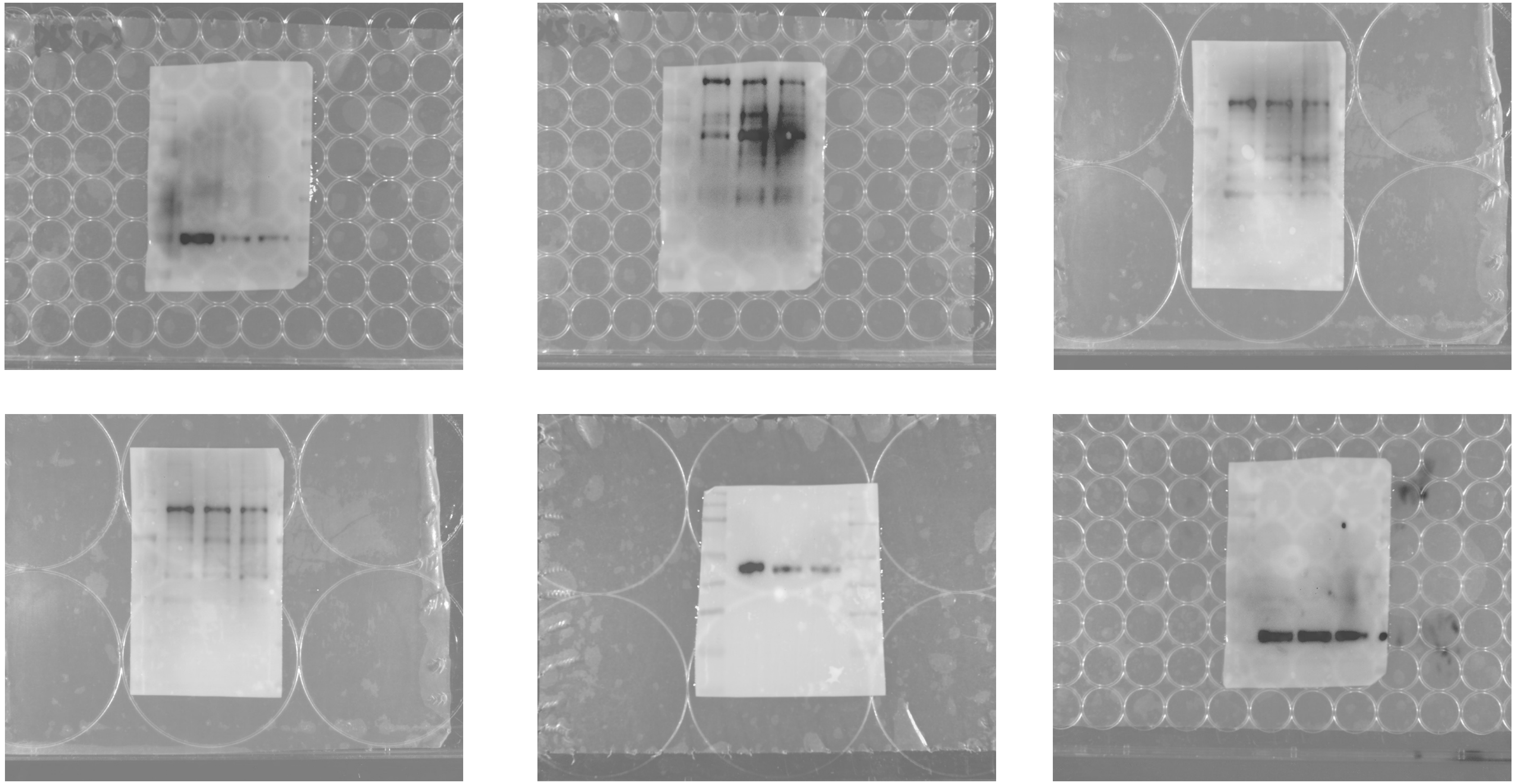

Fig3F

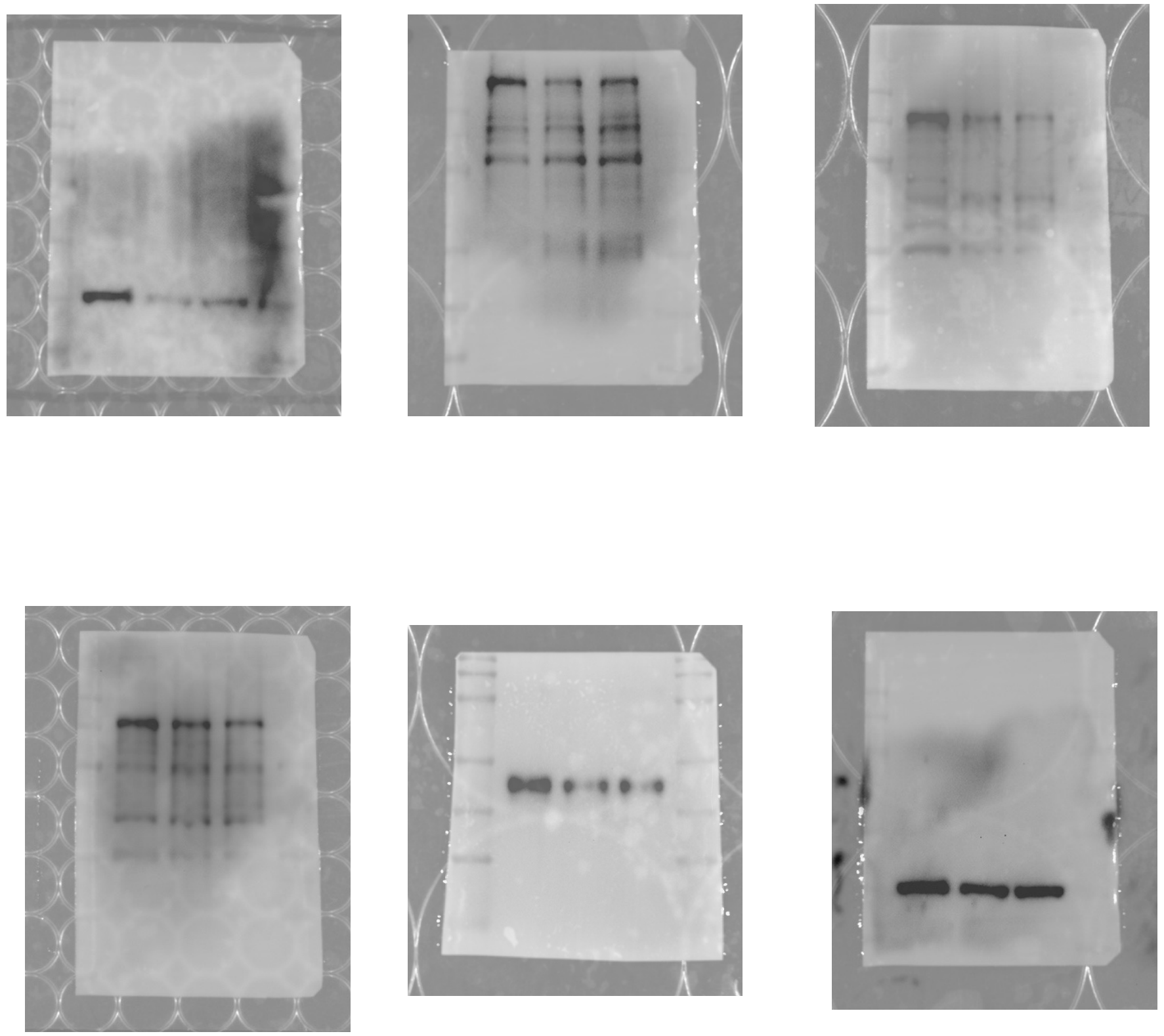

Fig3G

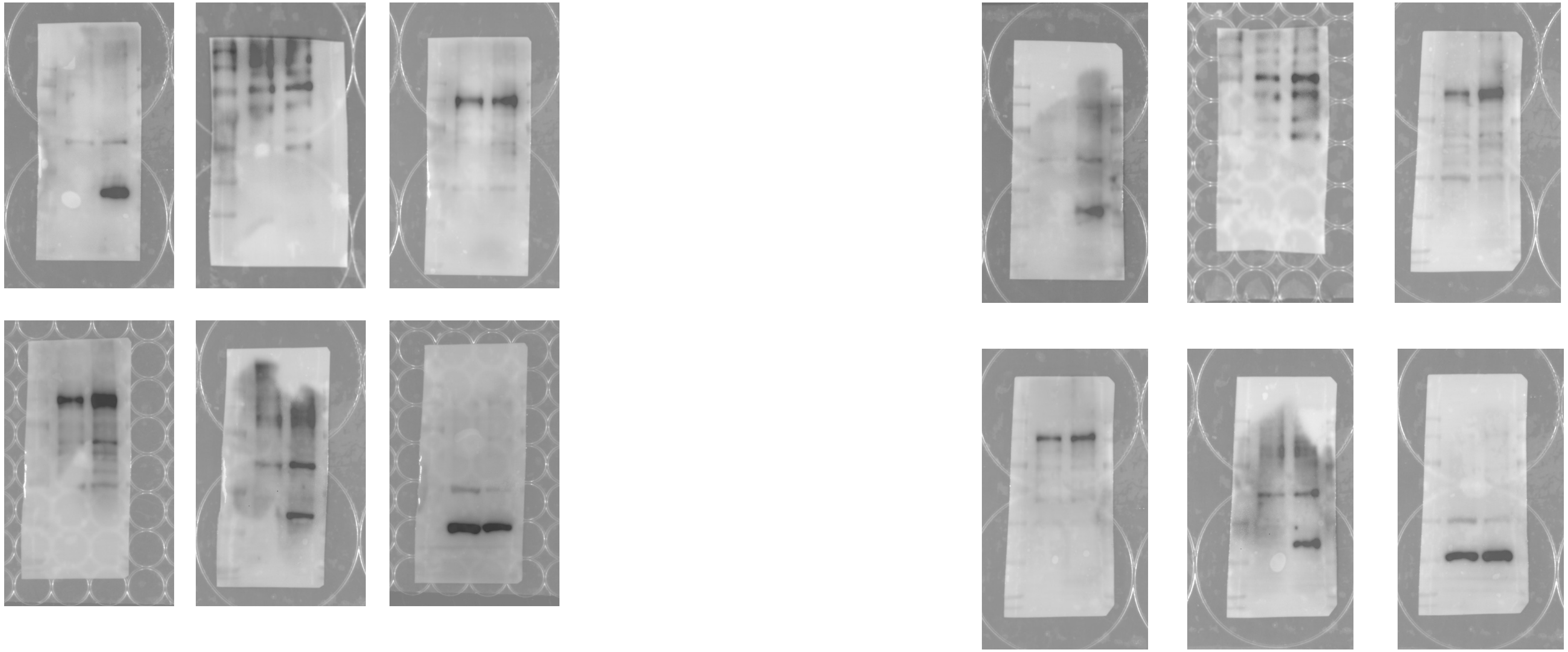

Fig3H

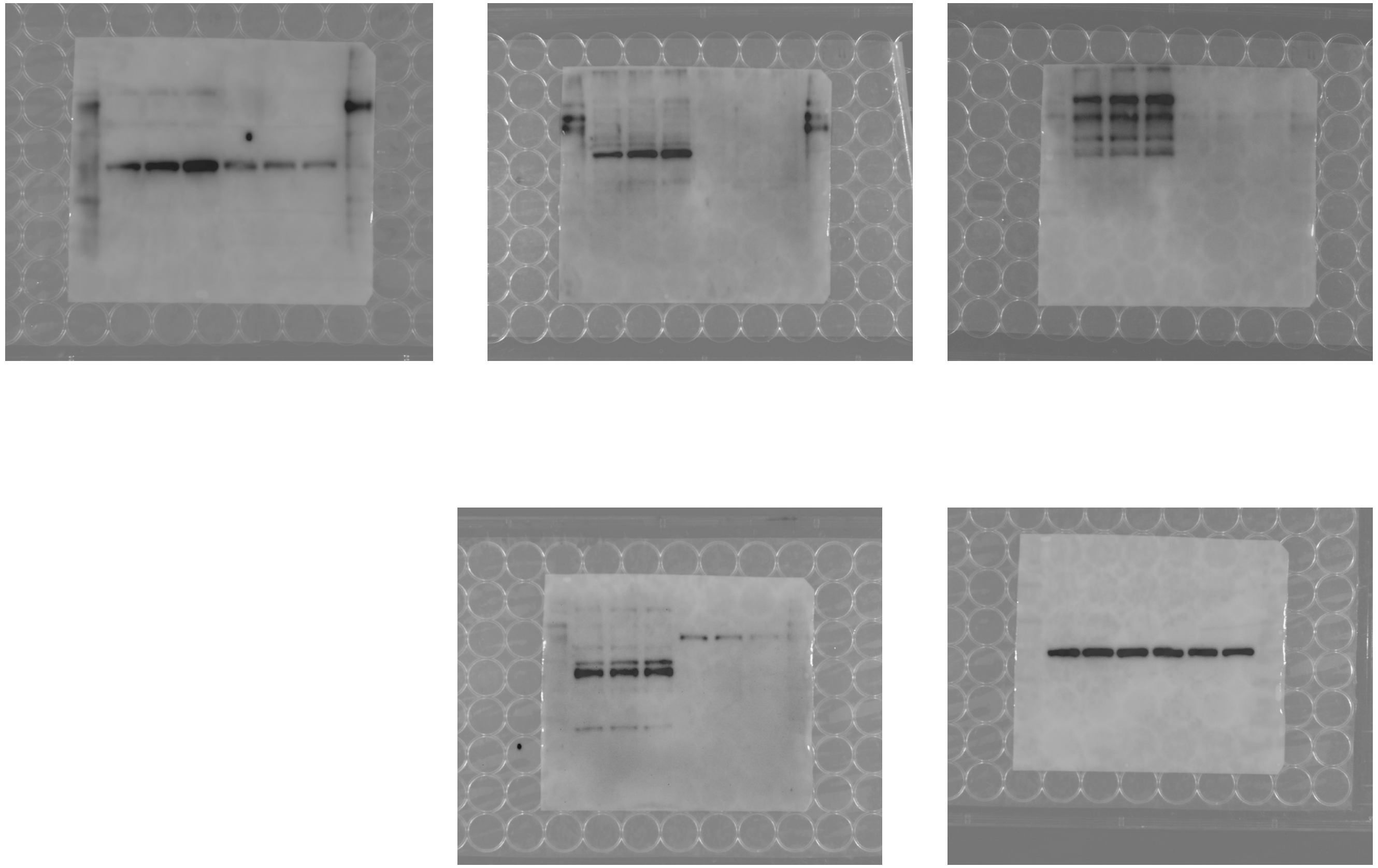

Fig4E

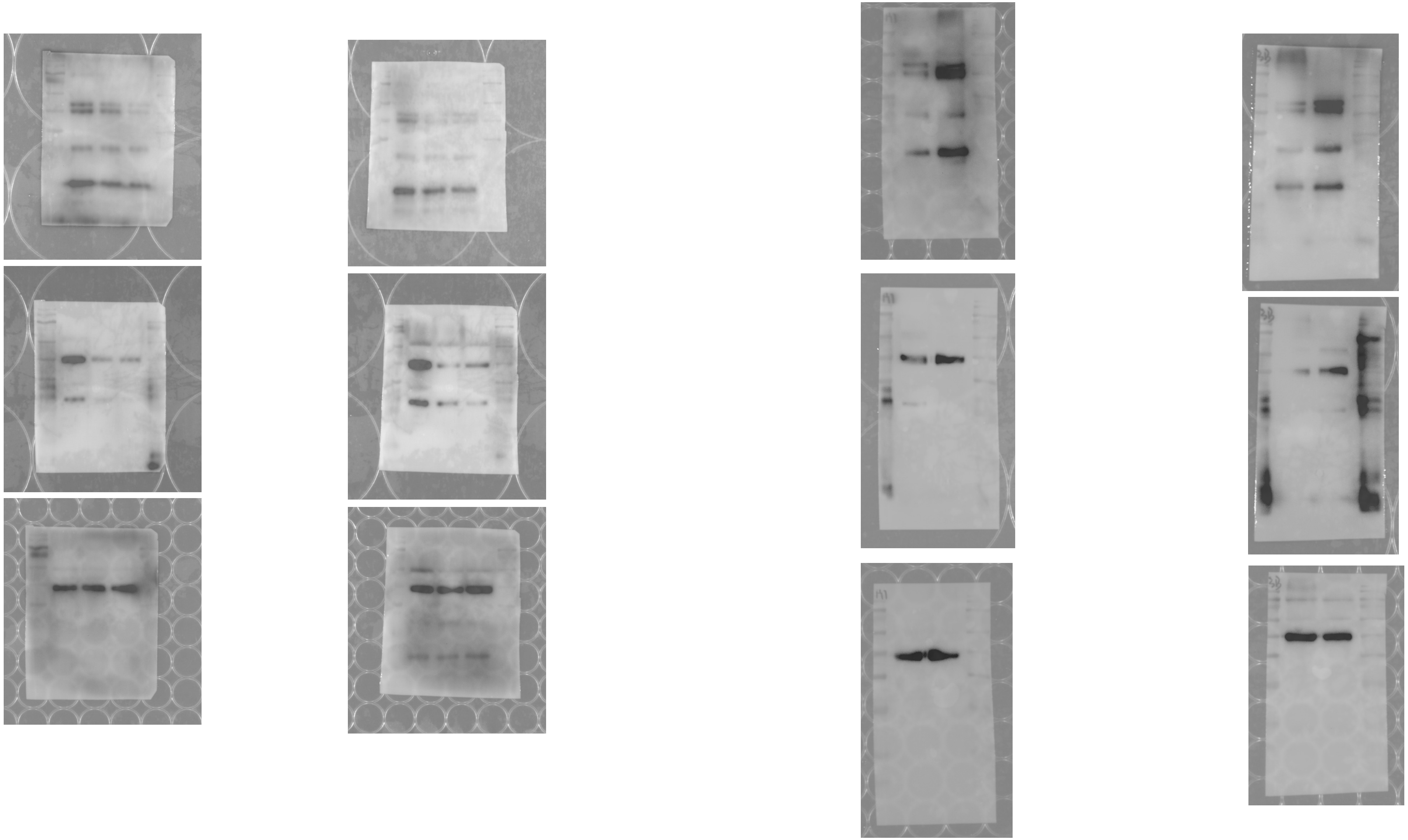

Fig4F

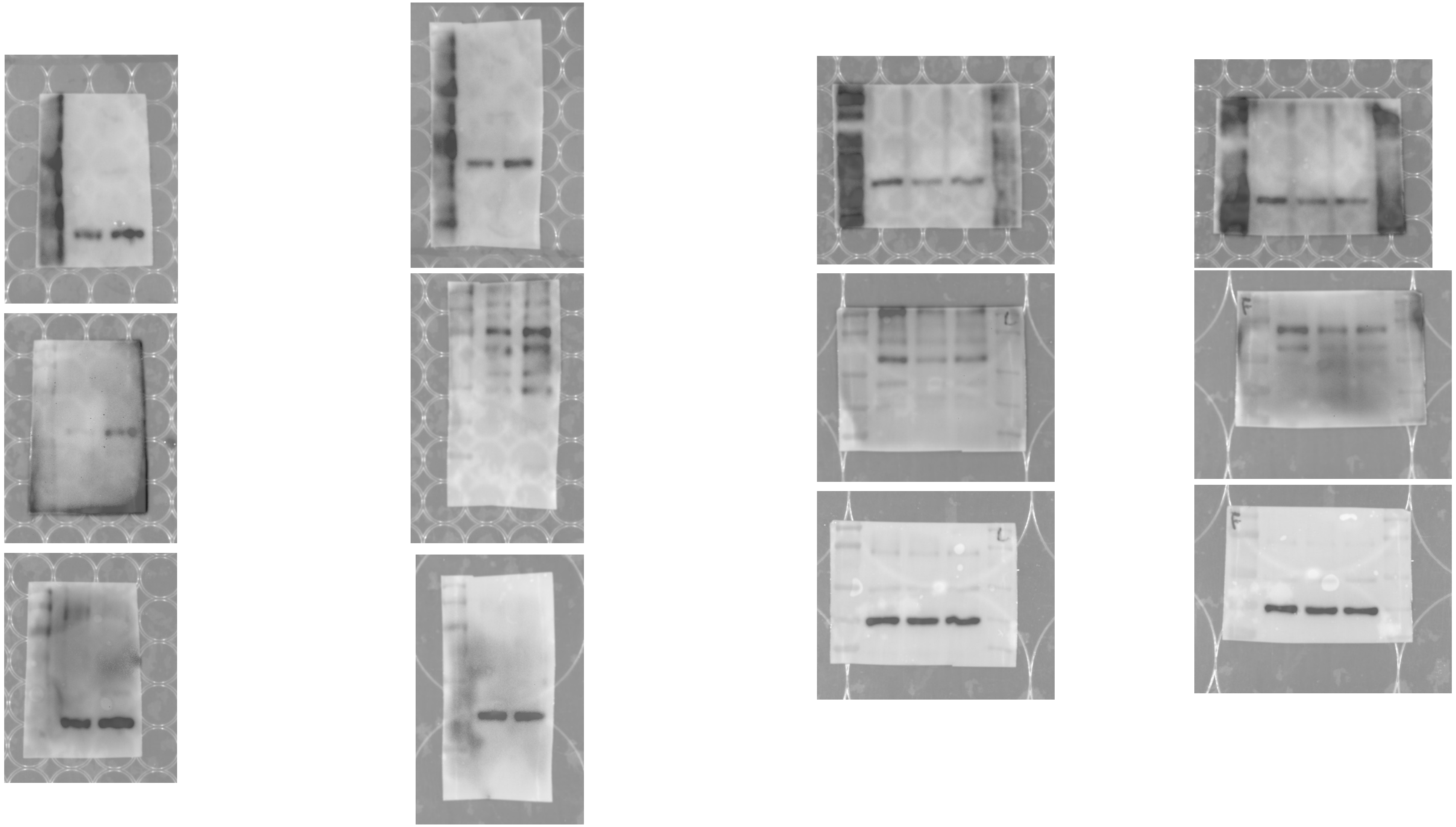

Fig4O

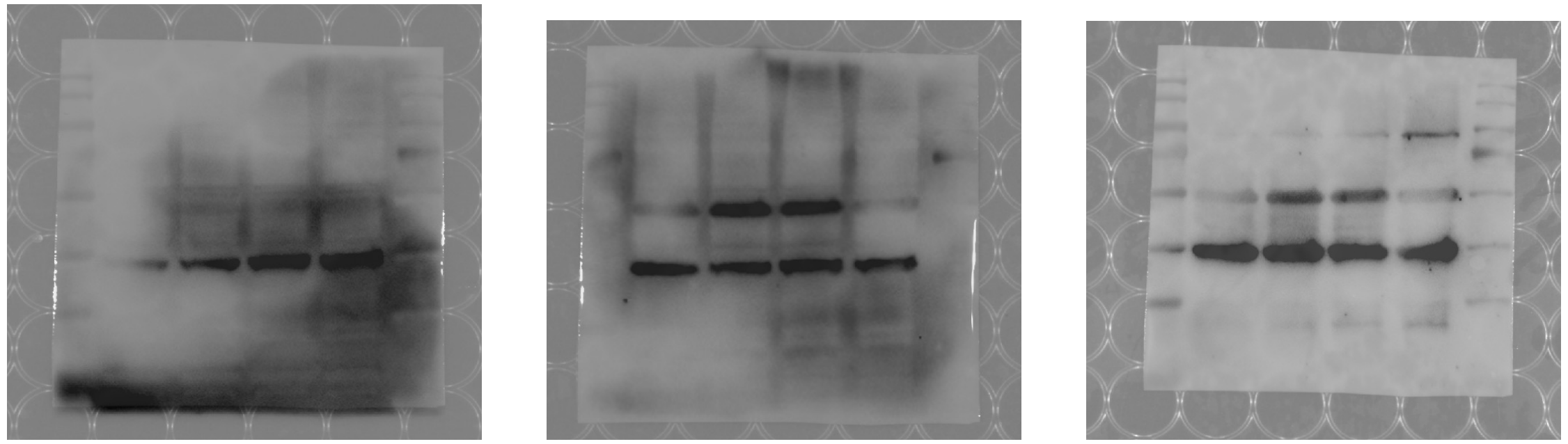

Fig5A

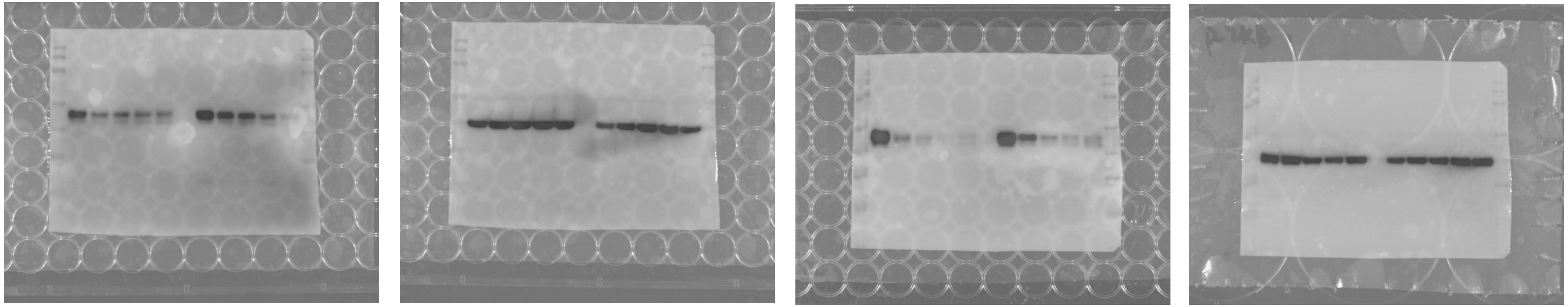

Fig5B

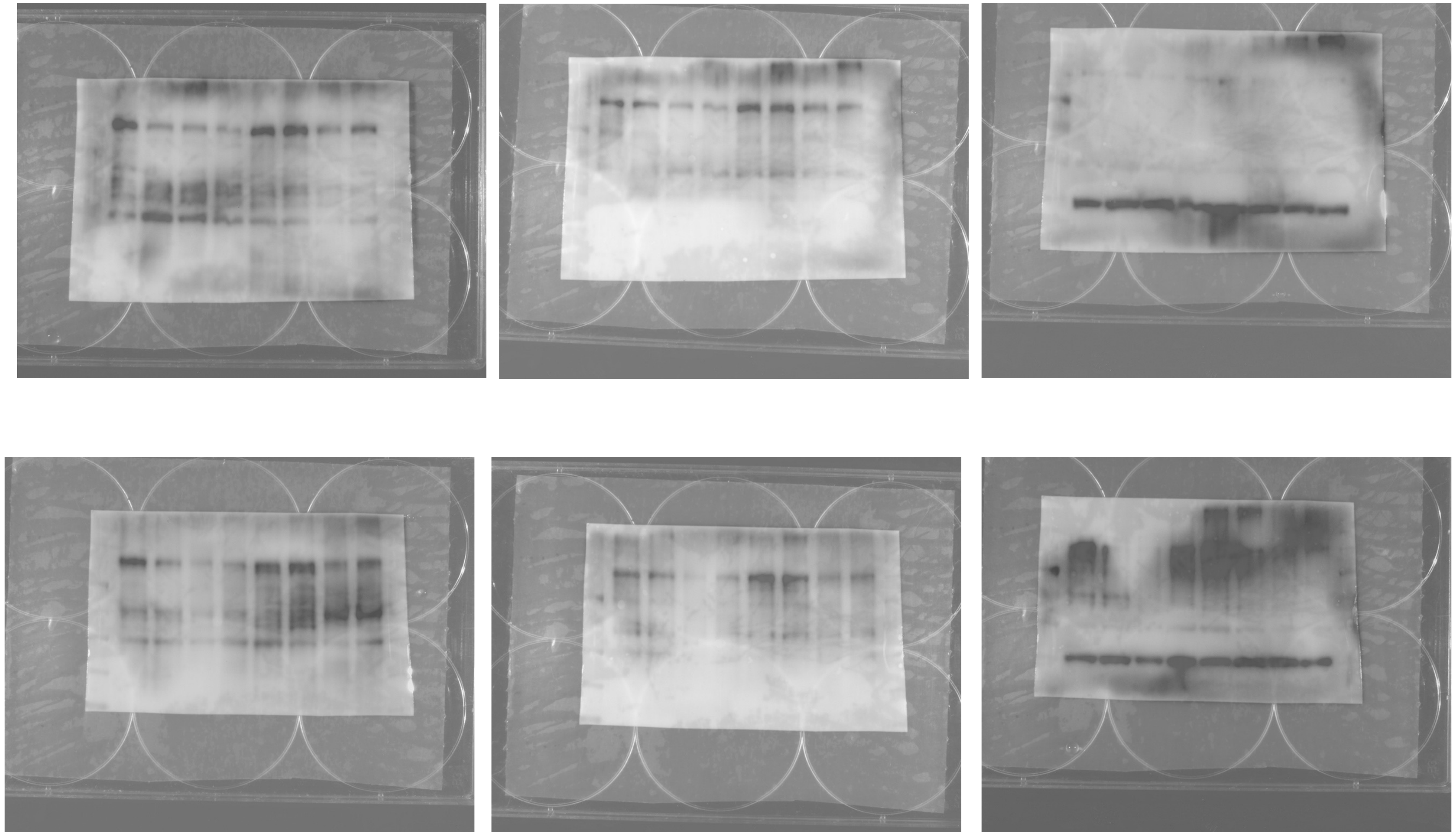

Fig5C

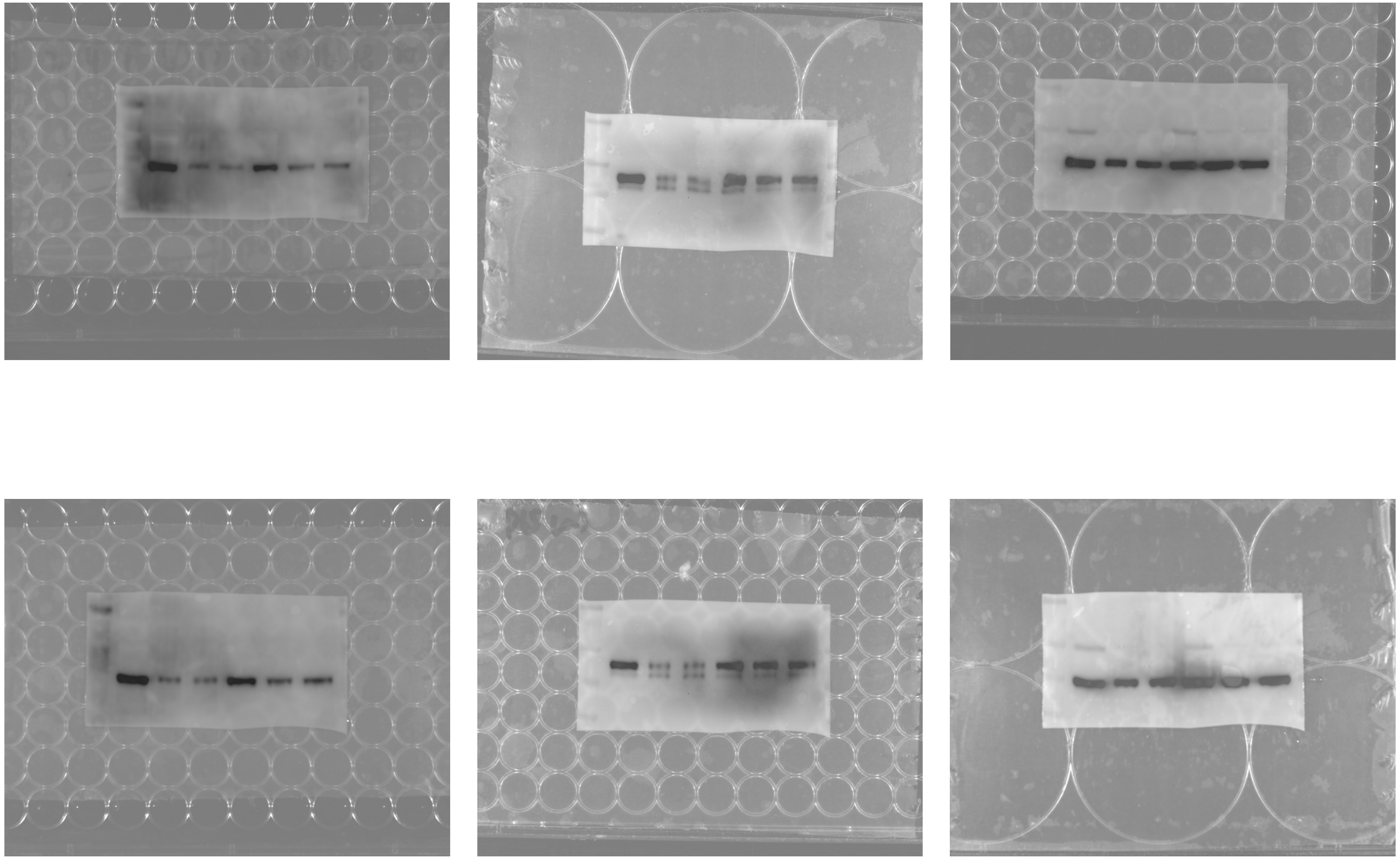

Fig5C

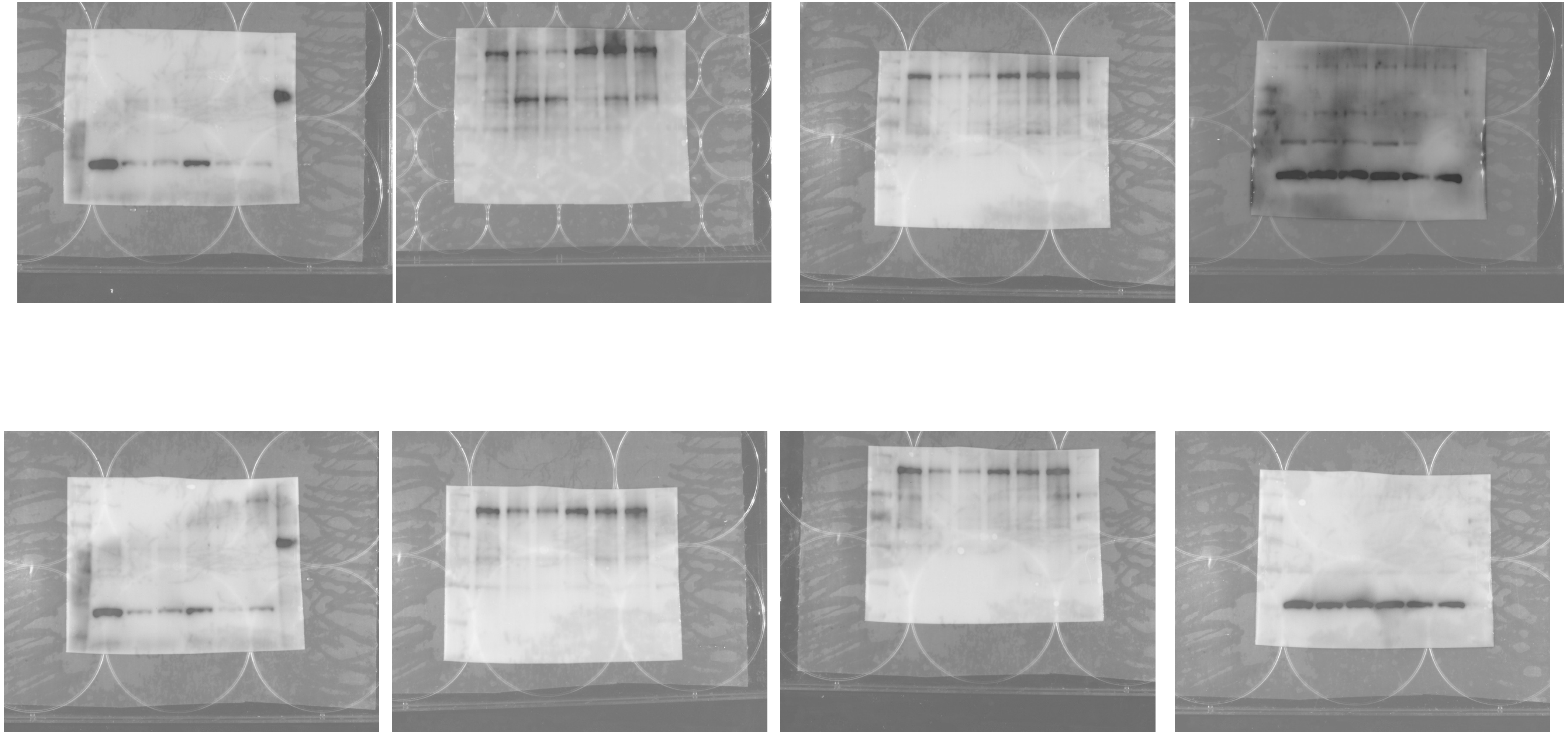

Fig5D

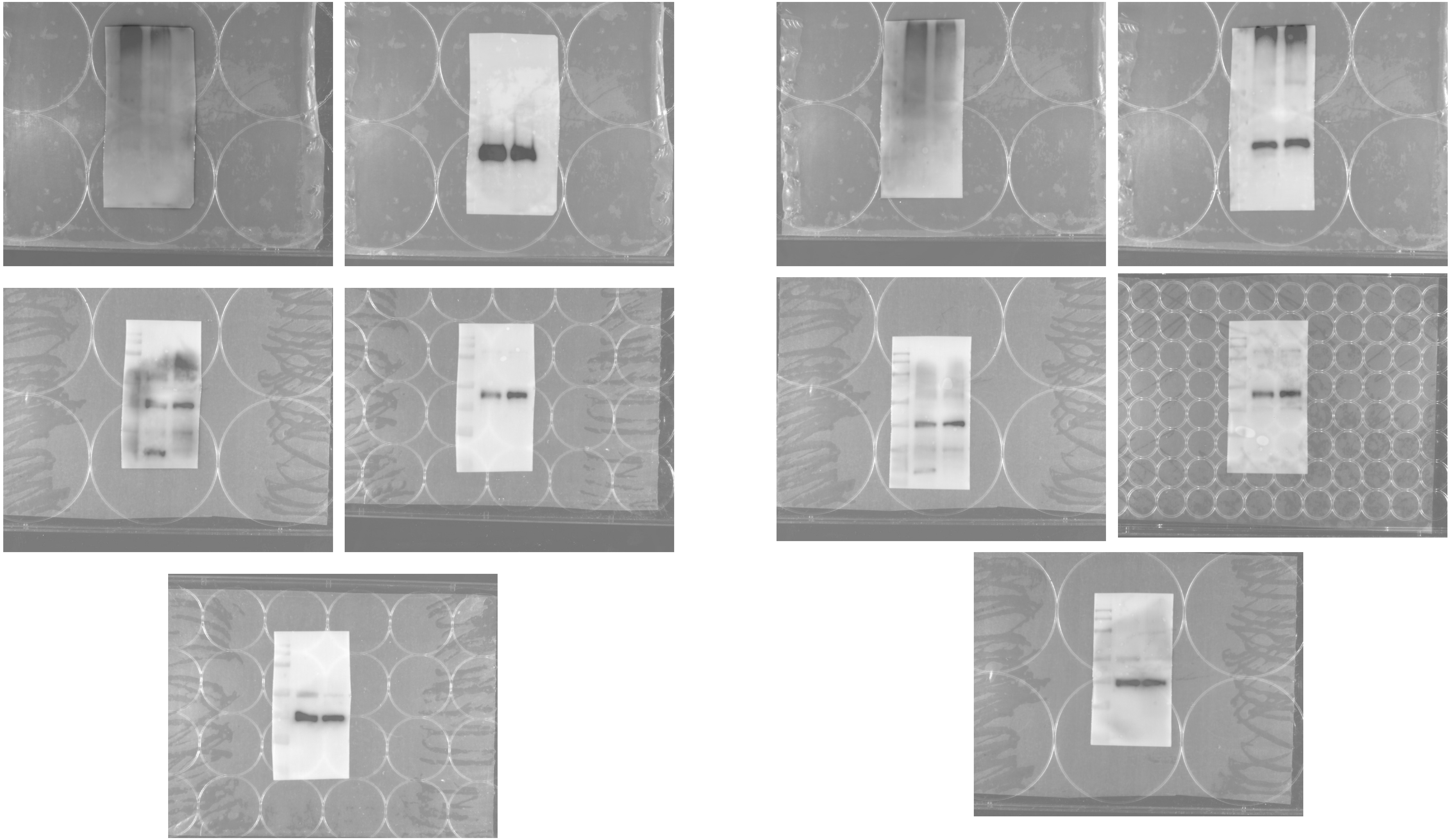

Fig5D

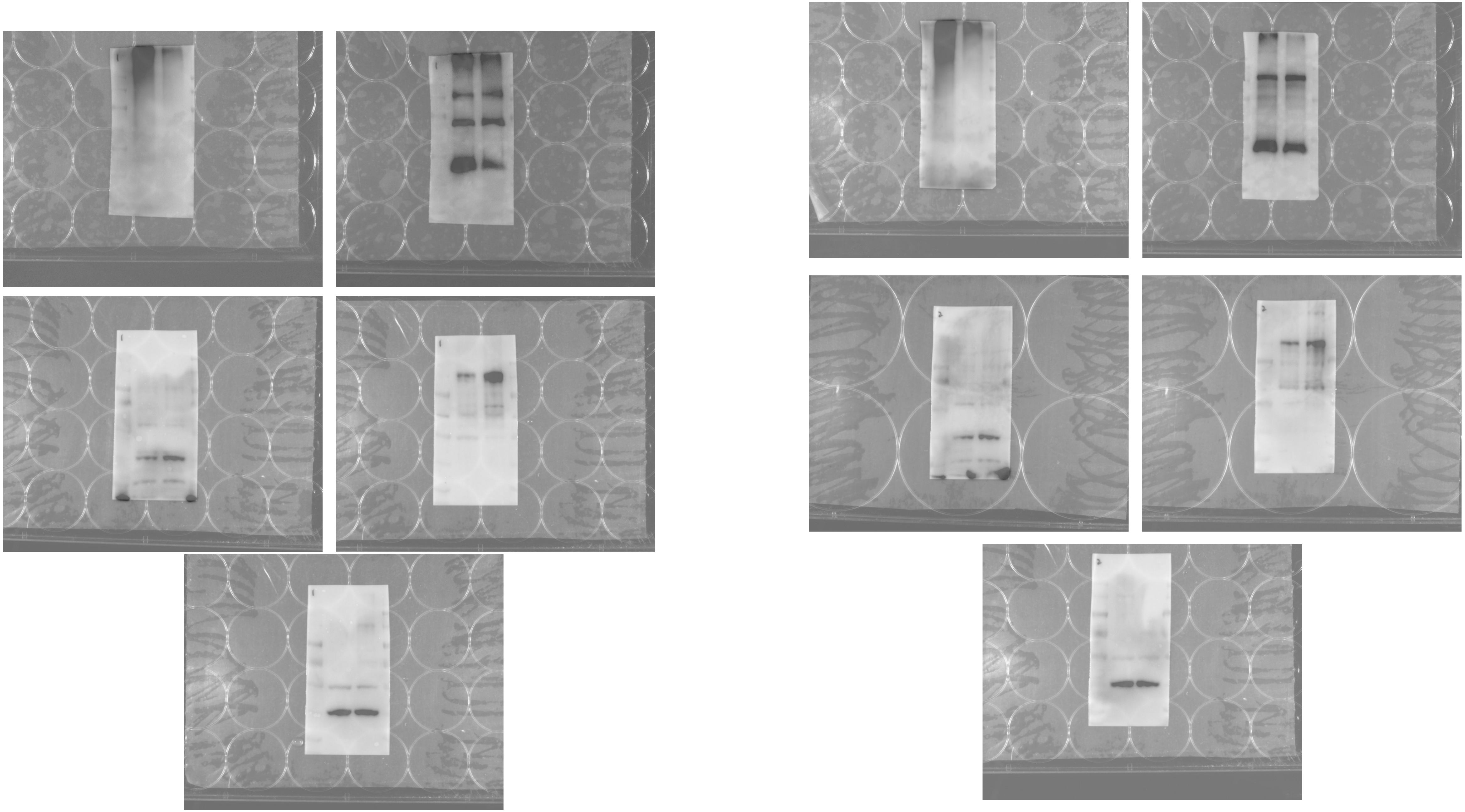

Fig5F

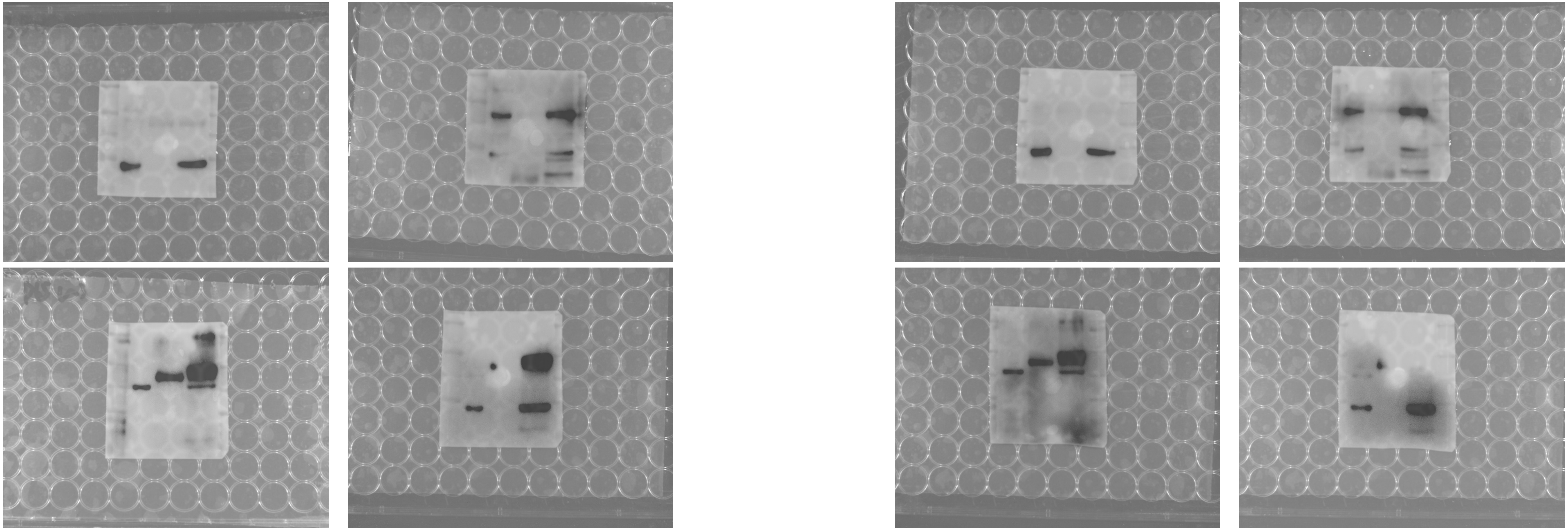

Fig5G

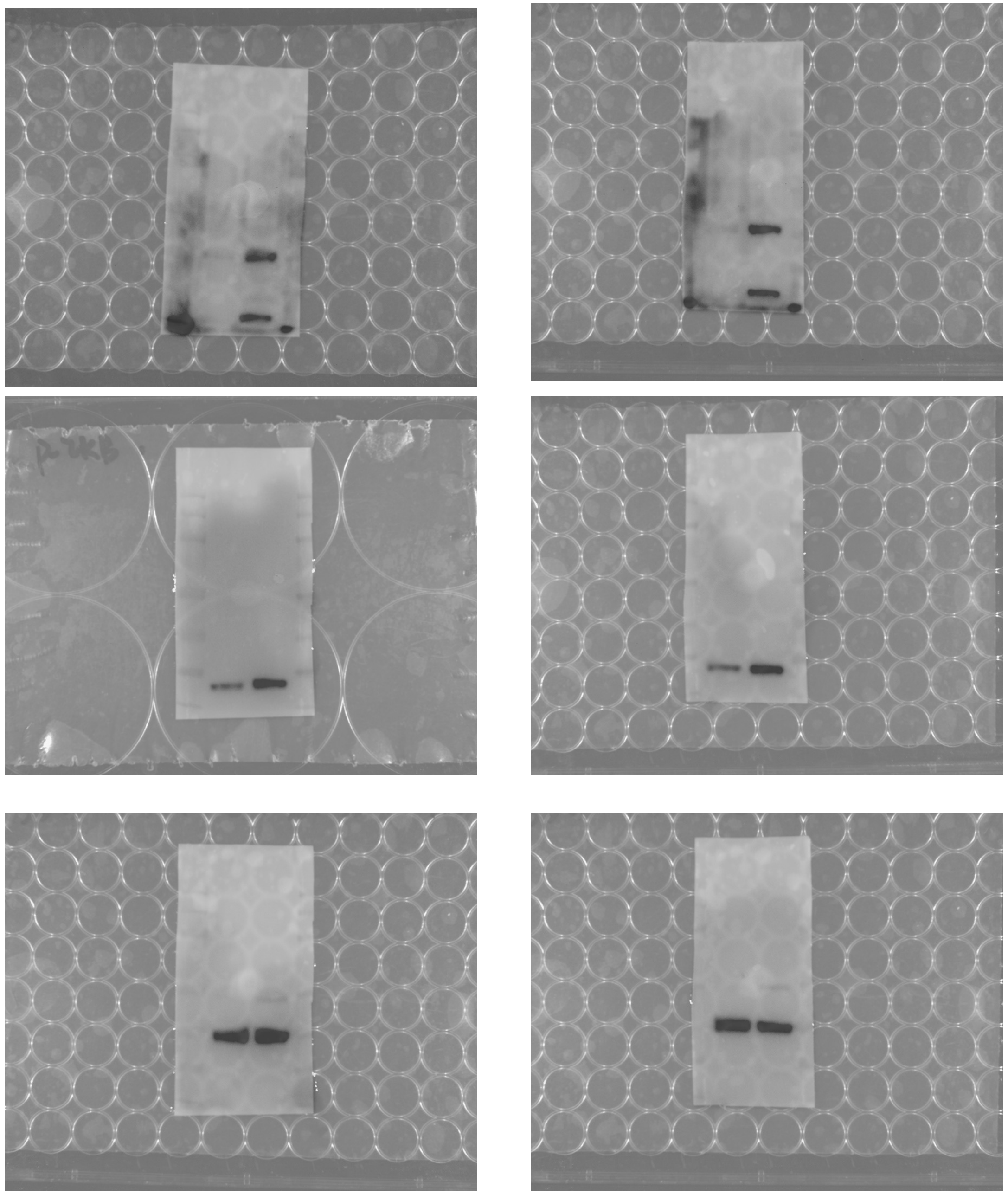

Fig5H

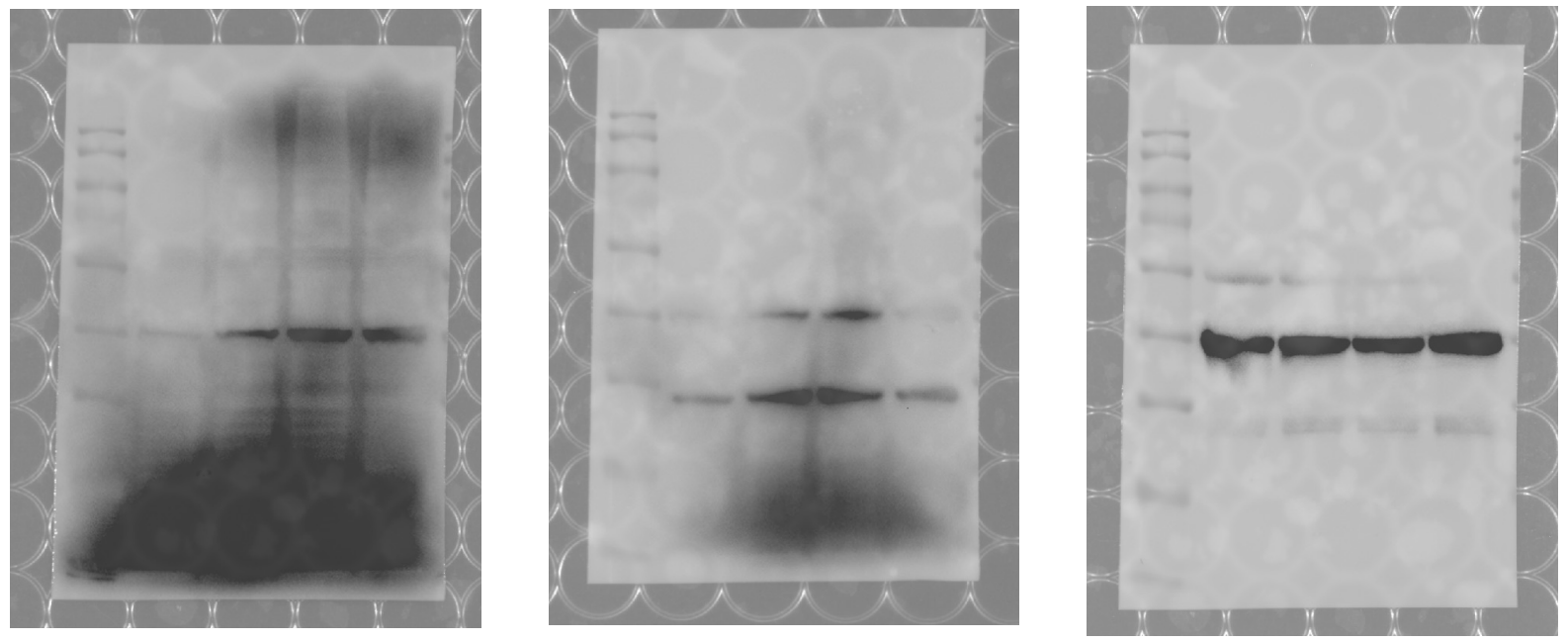

Fig6A

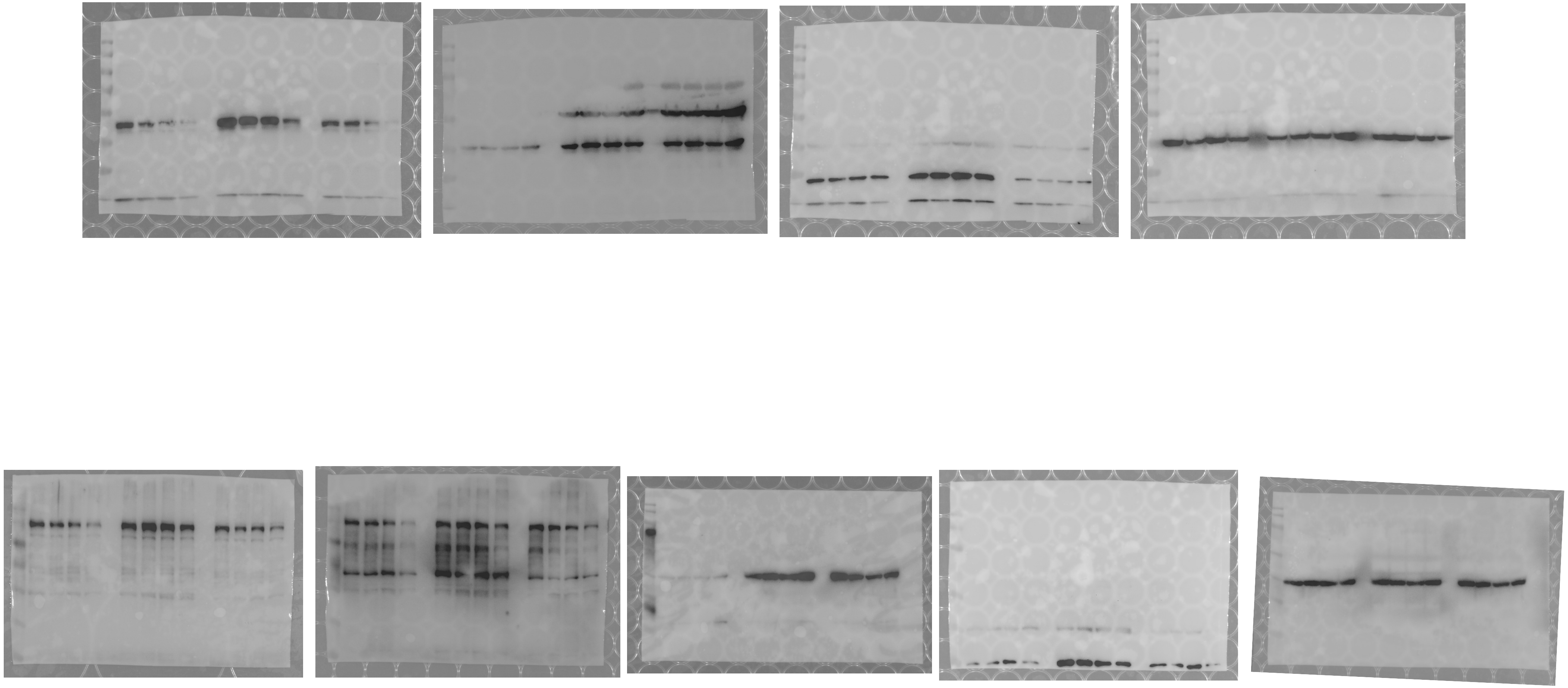

Fig6B

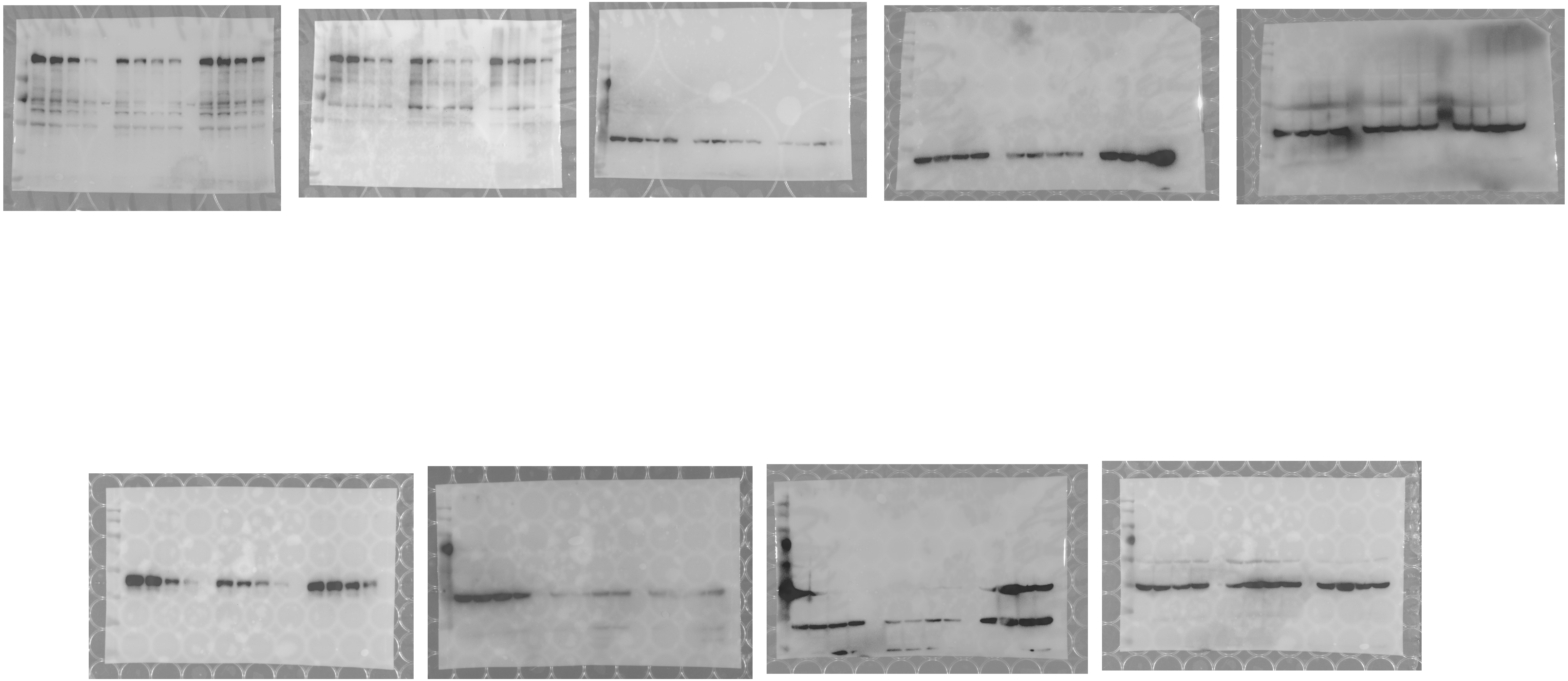

Fig6C

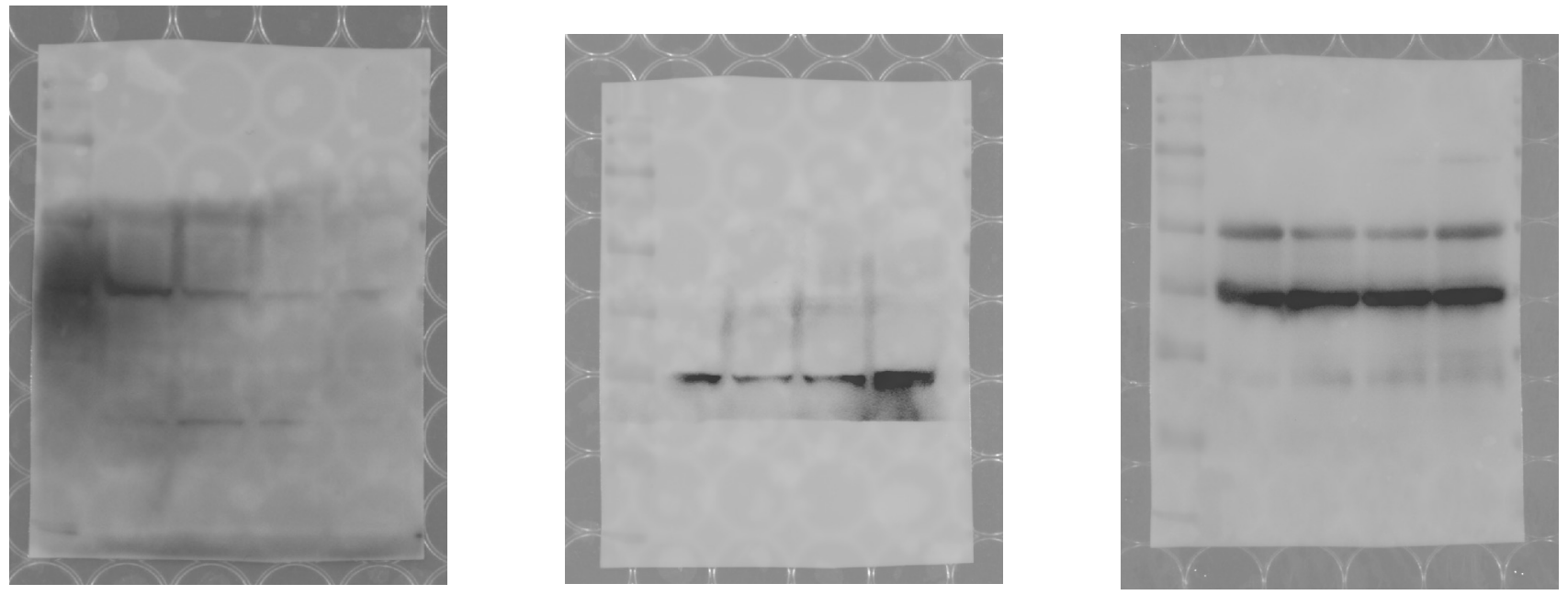

Fig6H

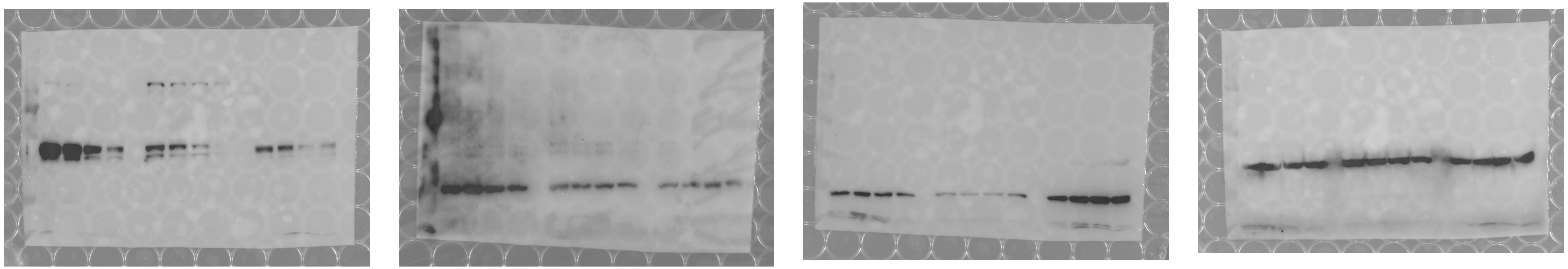

Fig6I

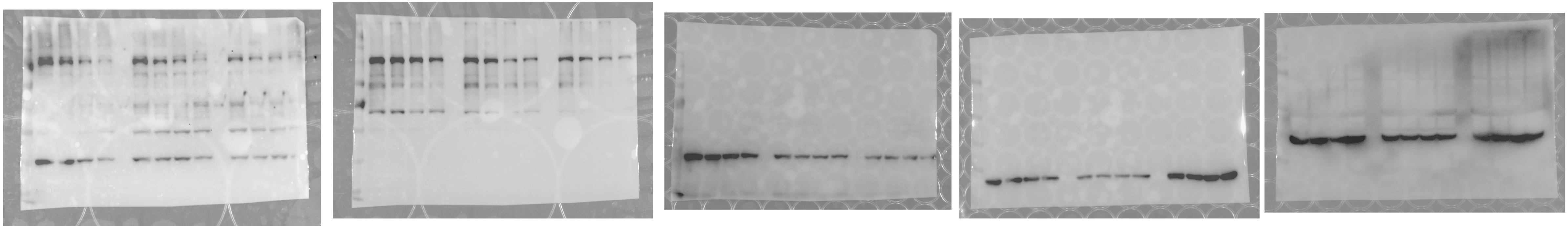

Fig6J

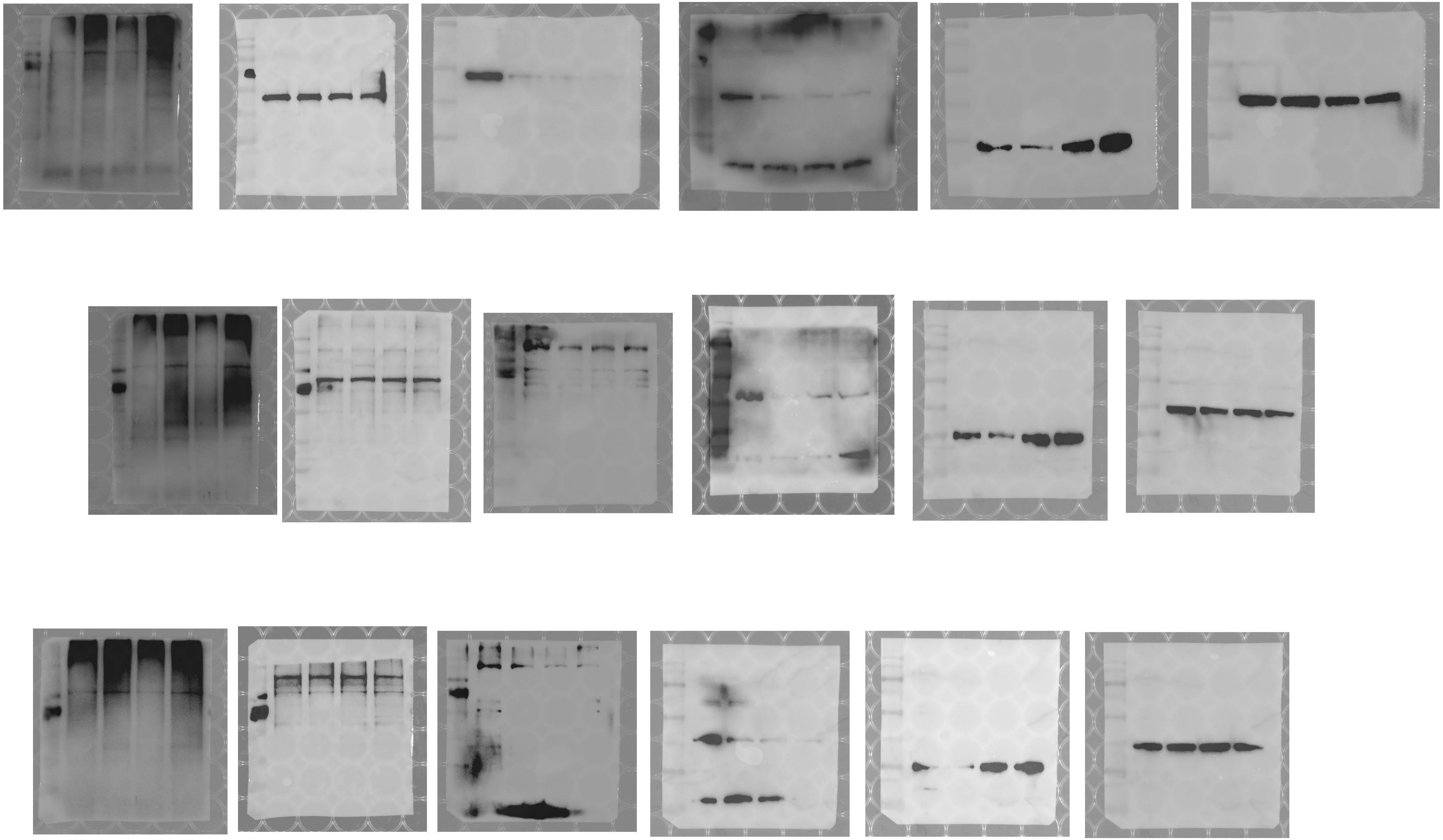

Fig7A

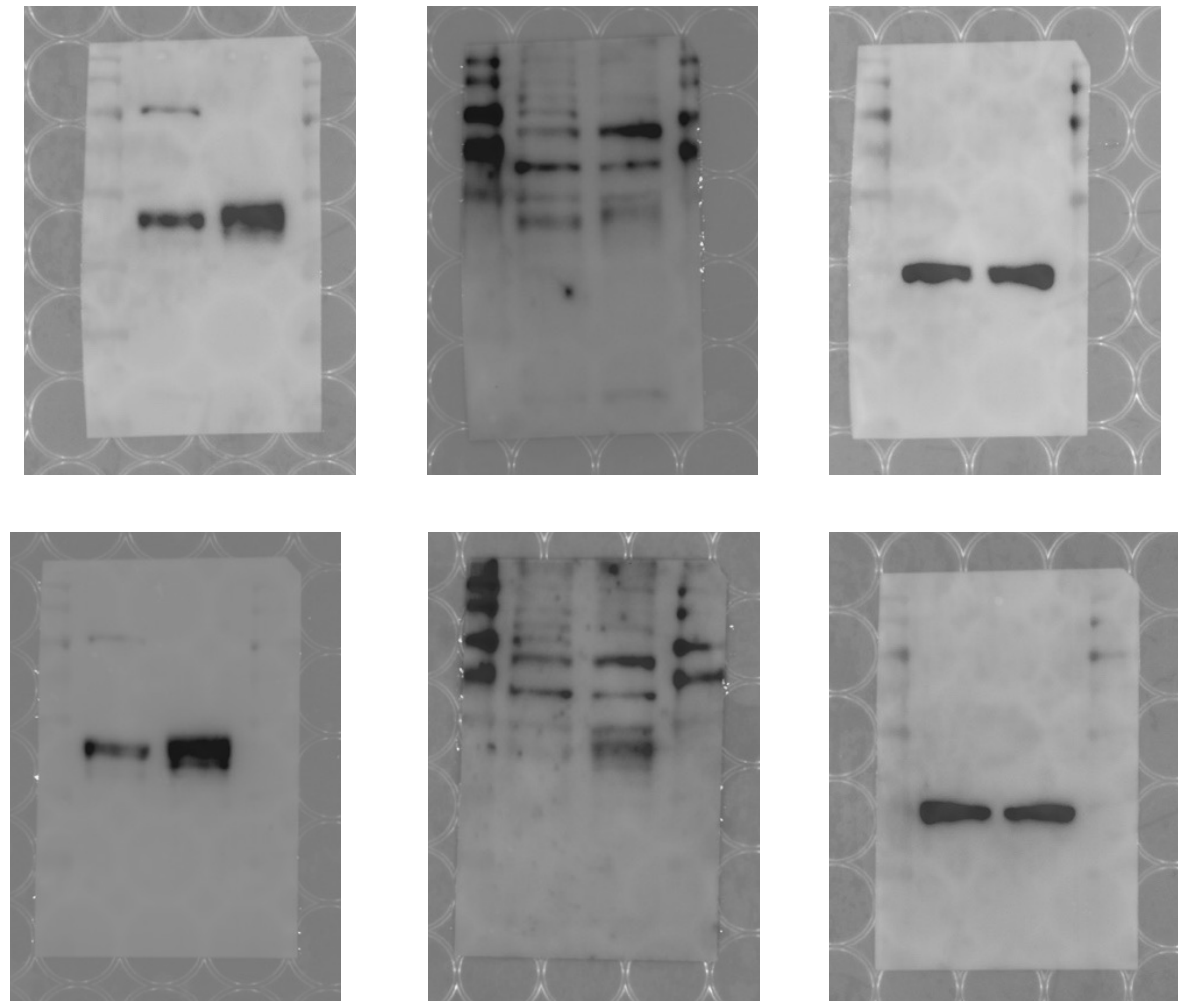

Fig7B

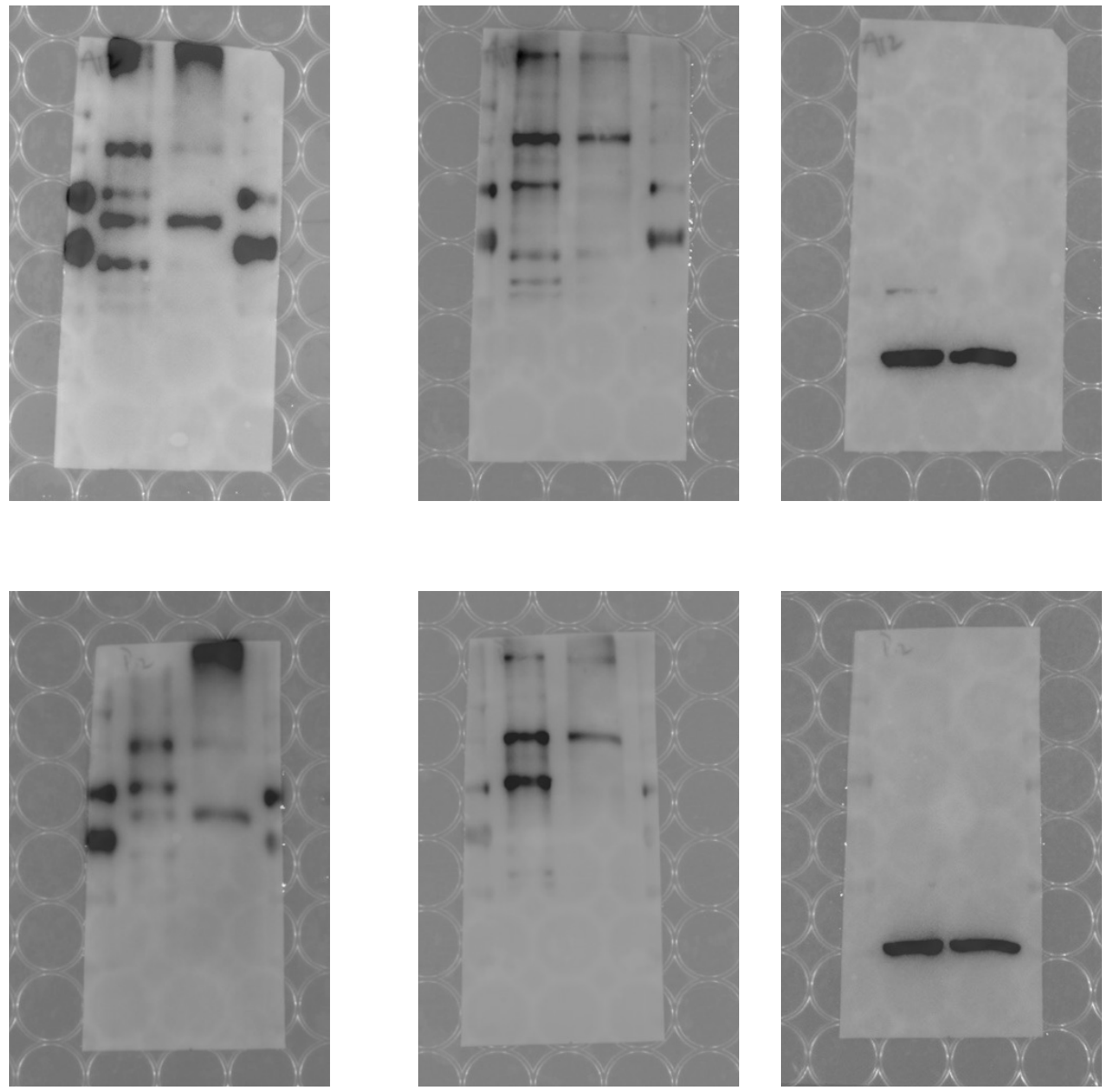

Fig7C

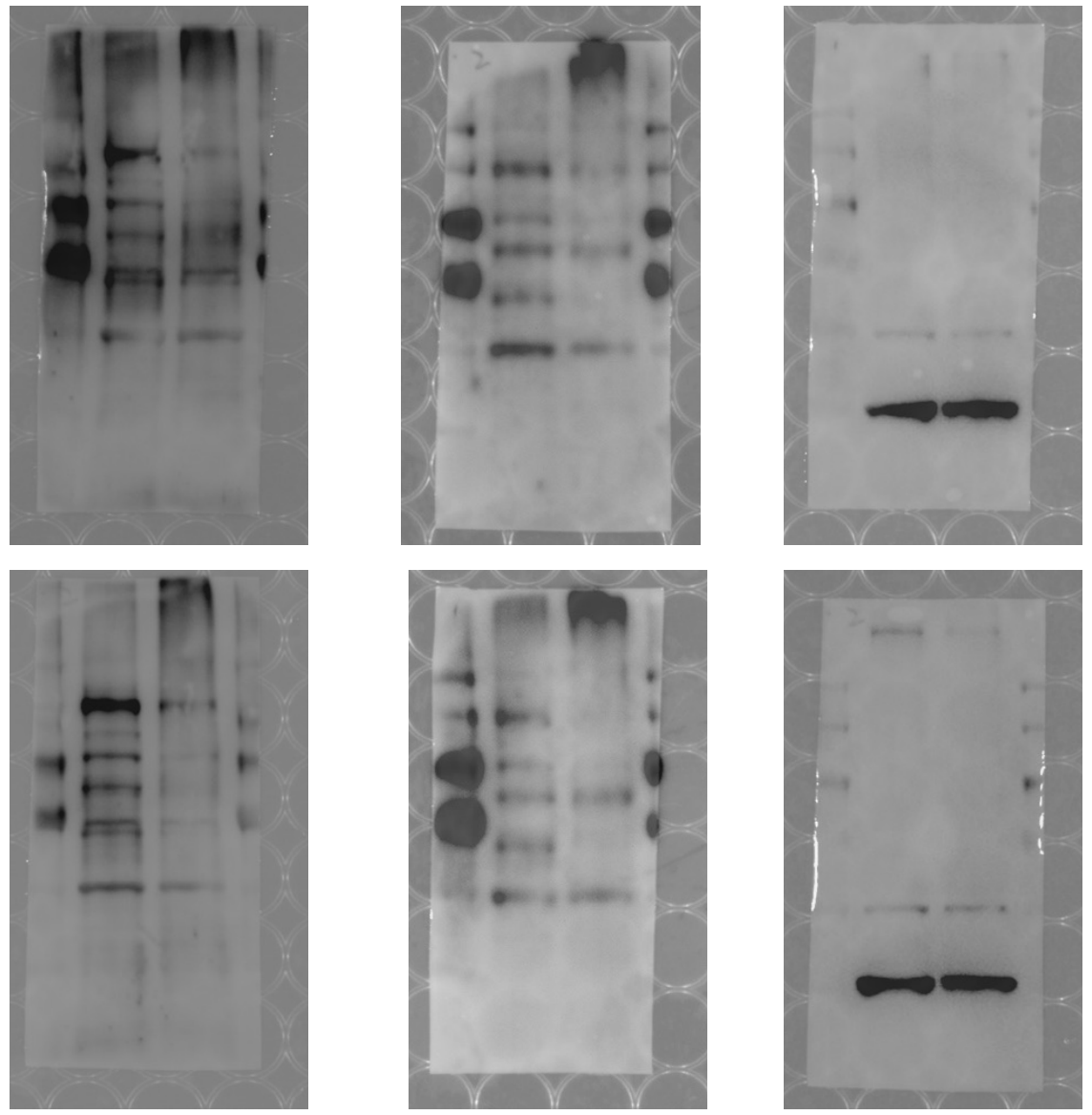

Fig8D

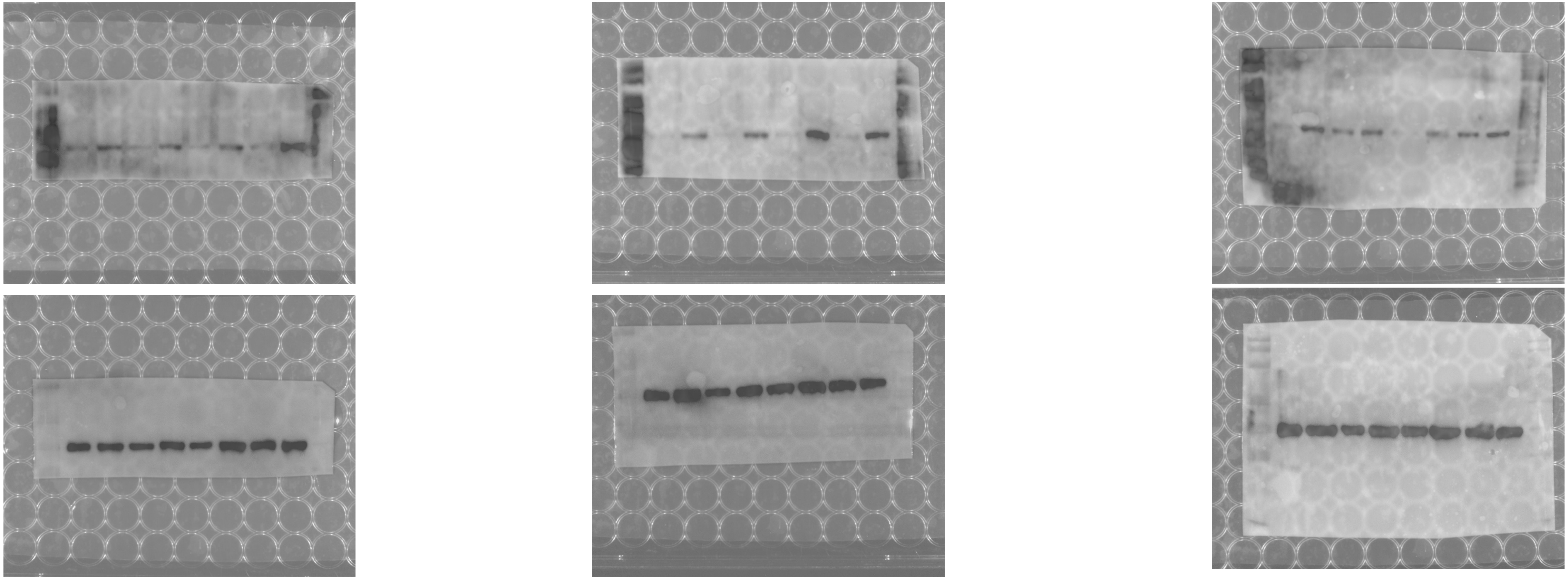

Supplement Fig.S1B

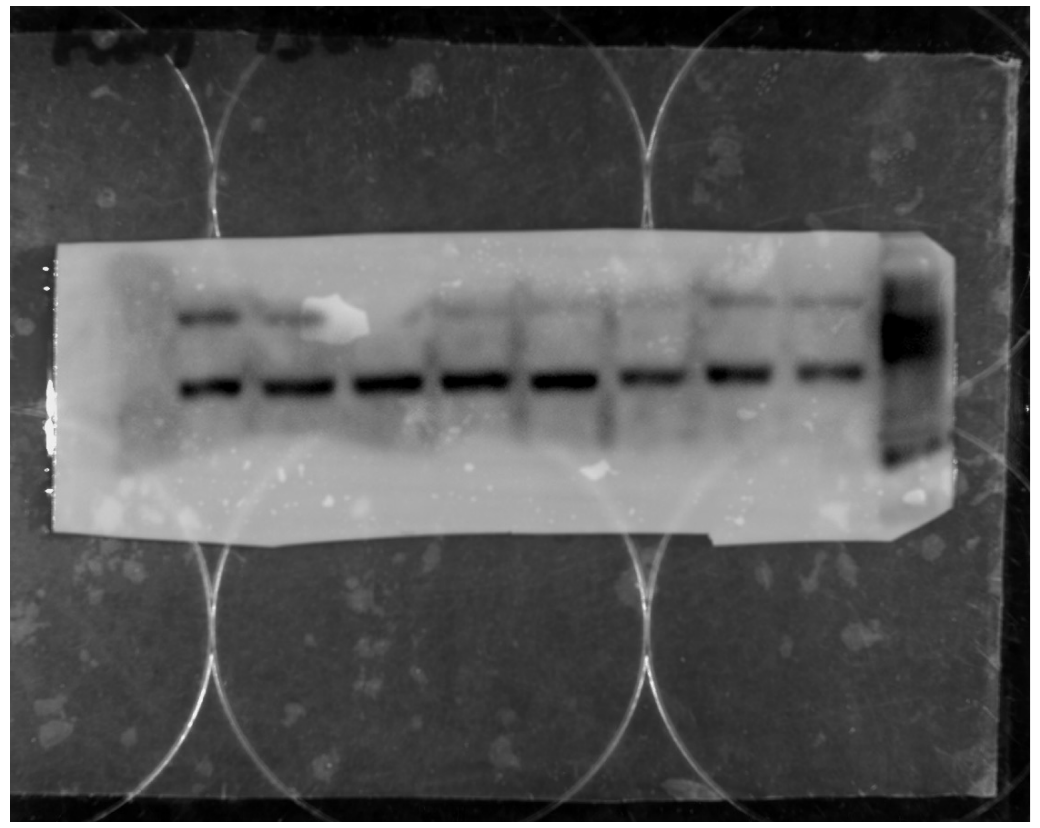

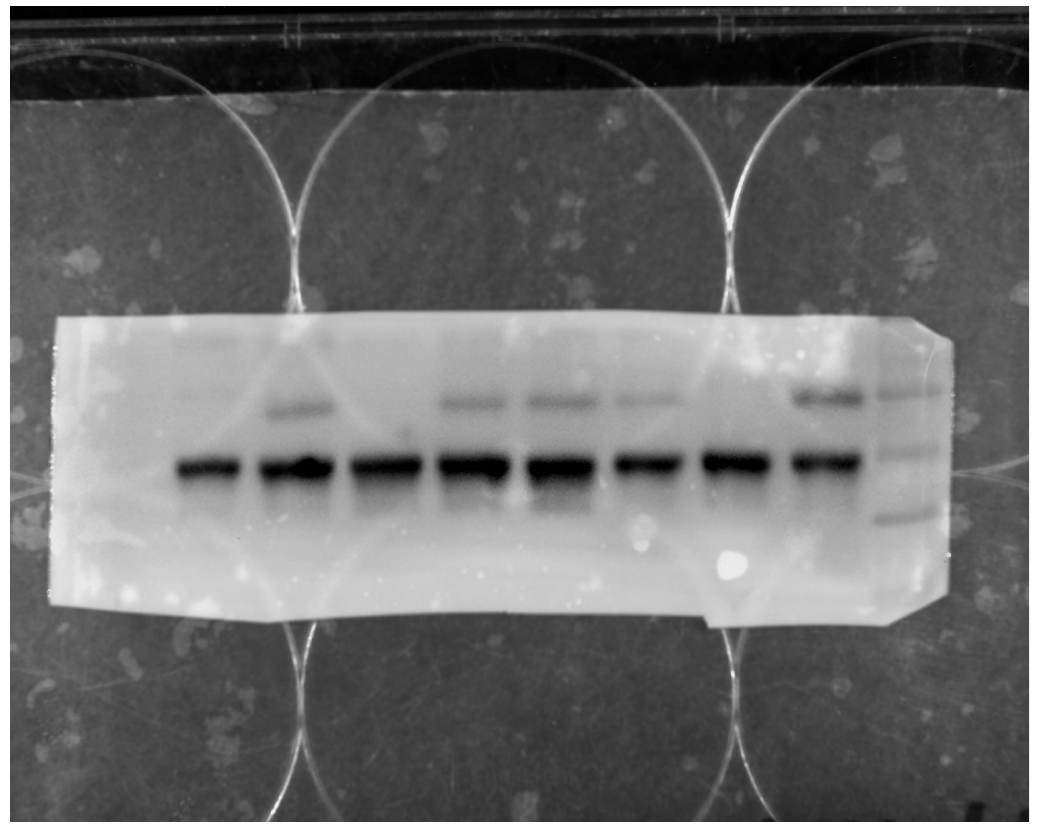

Supplementary Fig.S3A

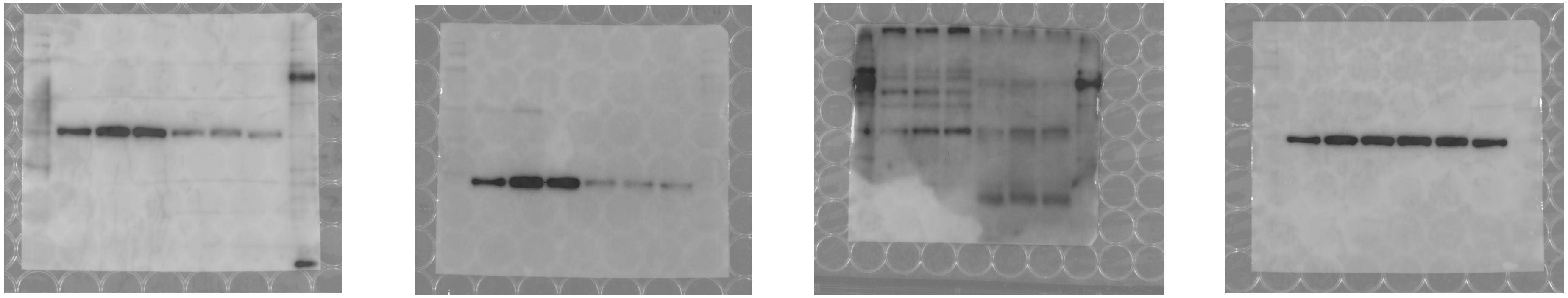

Supplementary Fig.S7A

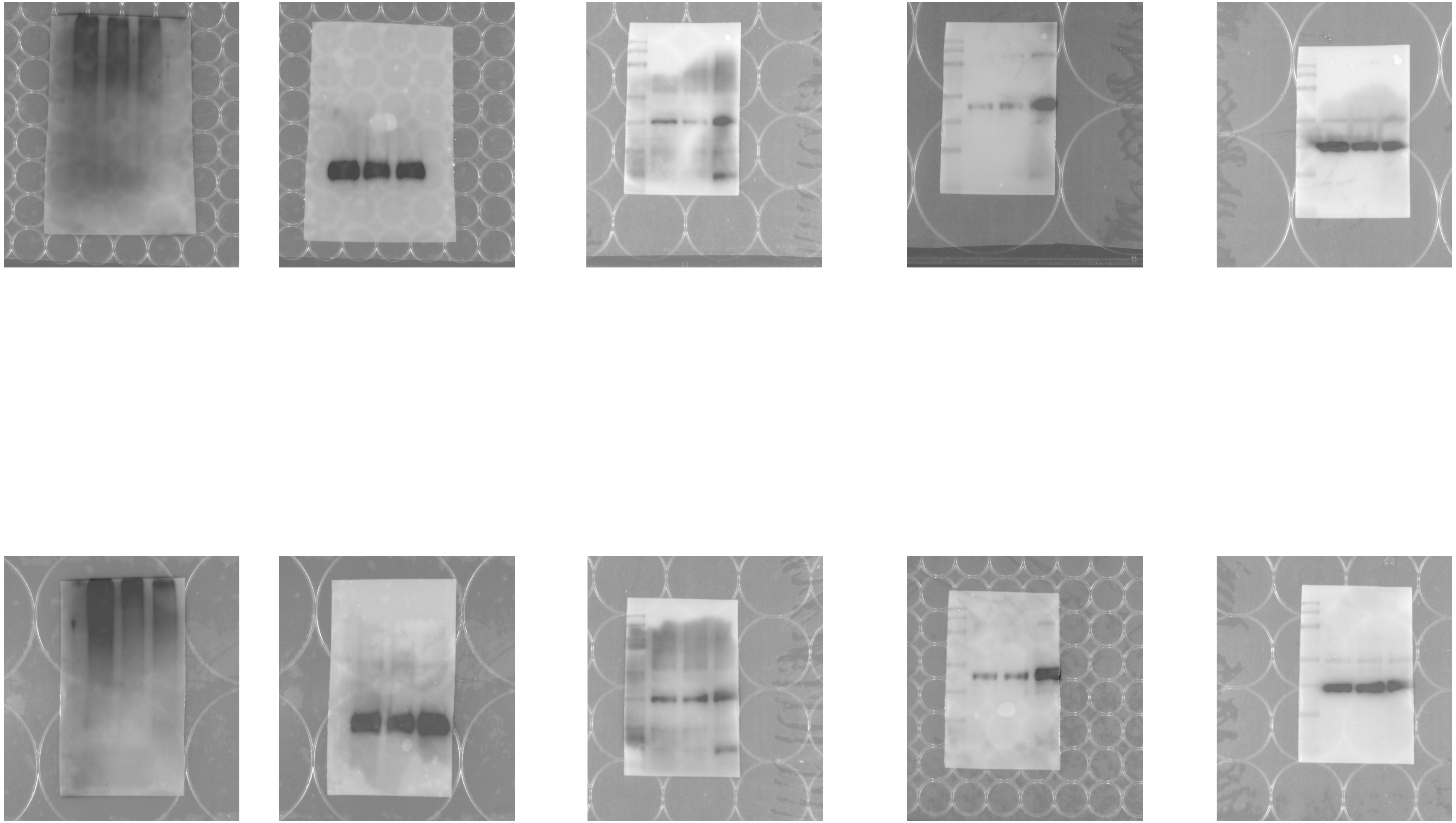

Supplementary Fig.S8C

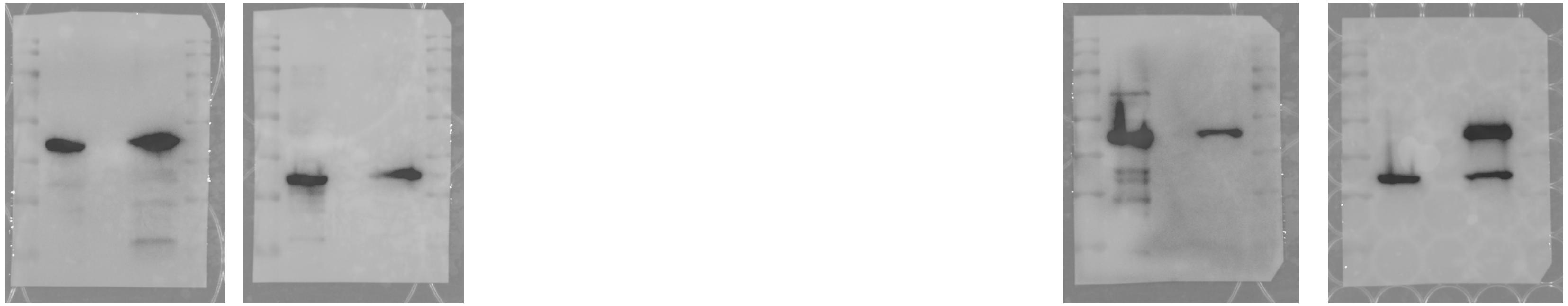

Supplementary Fig.S8D

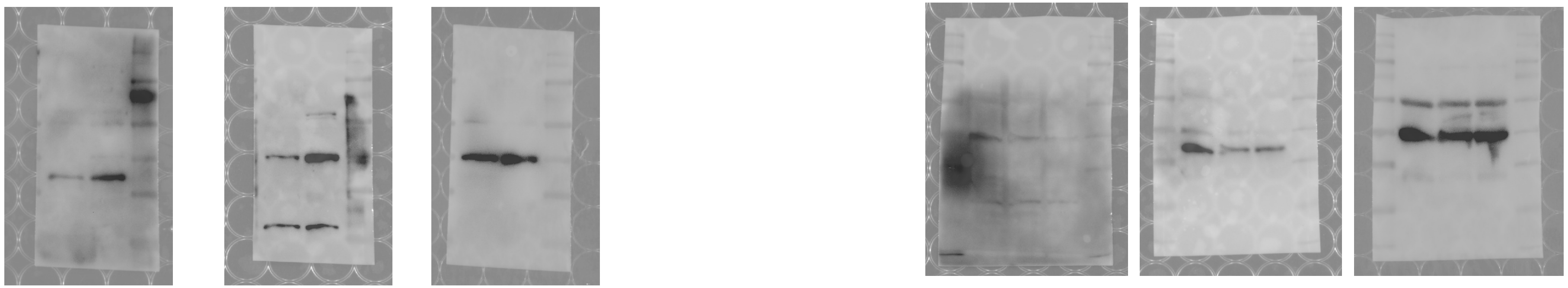

Supplementary Fig. S11A

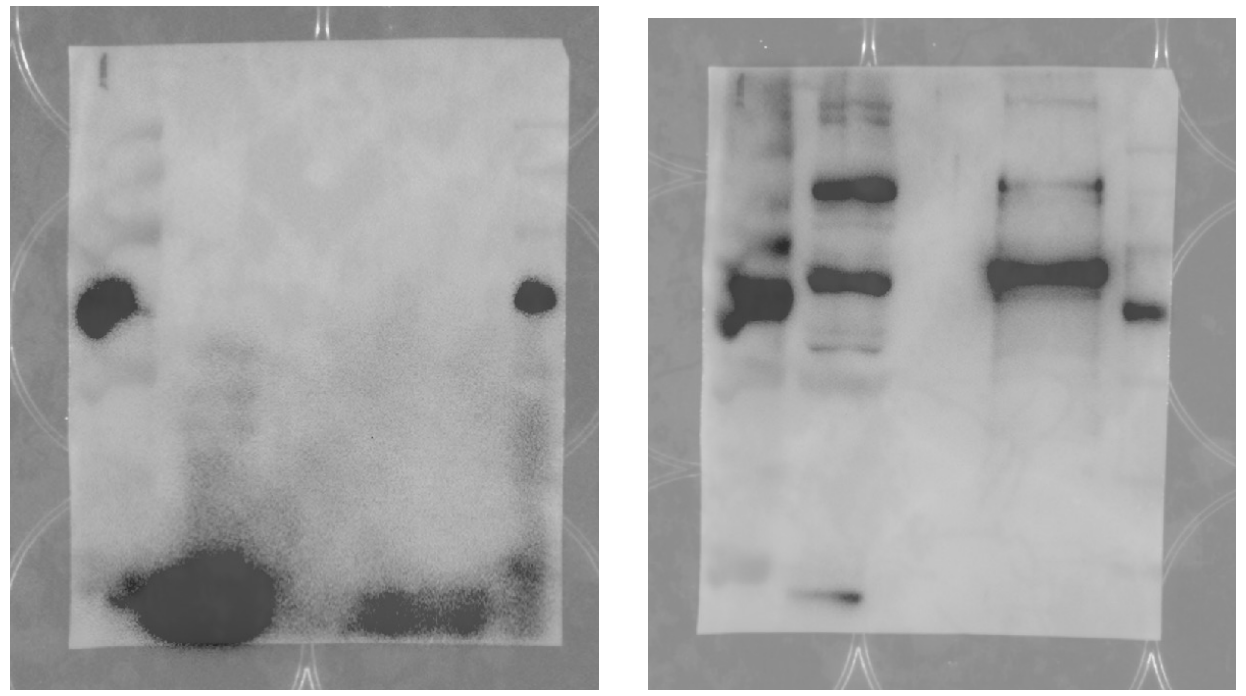

Supplementary Fig. S11B

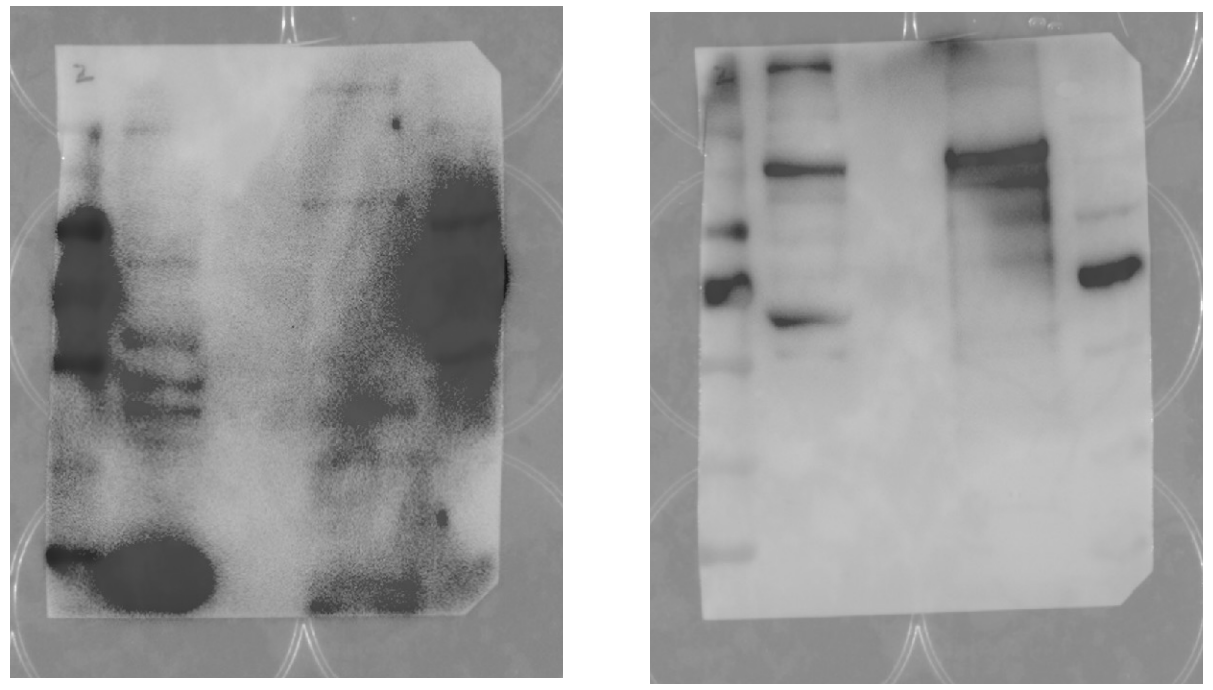

Supplementary Fig. S11C

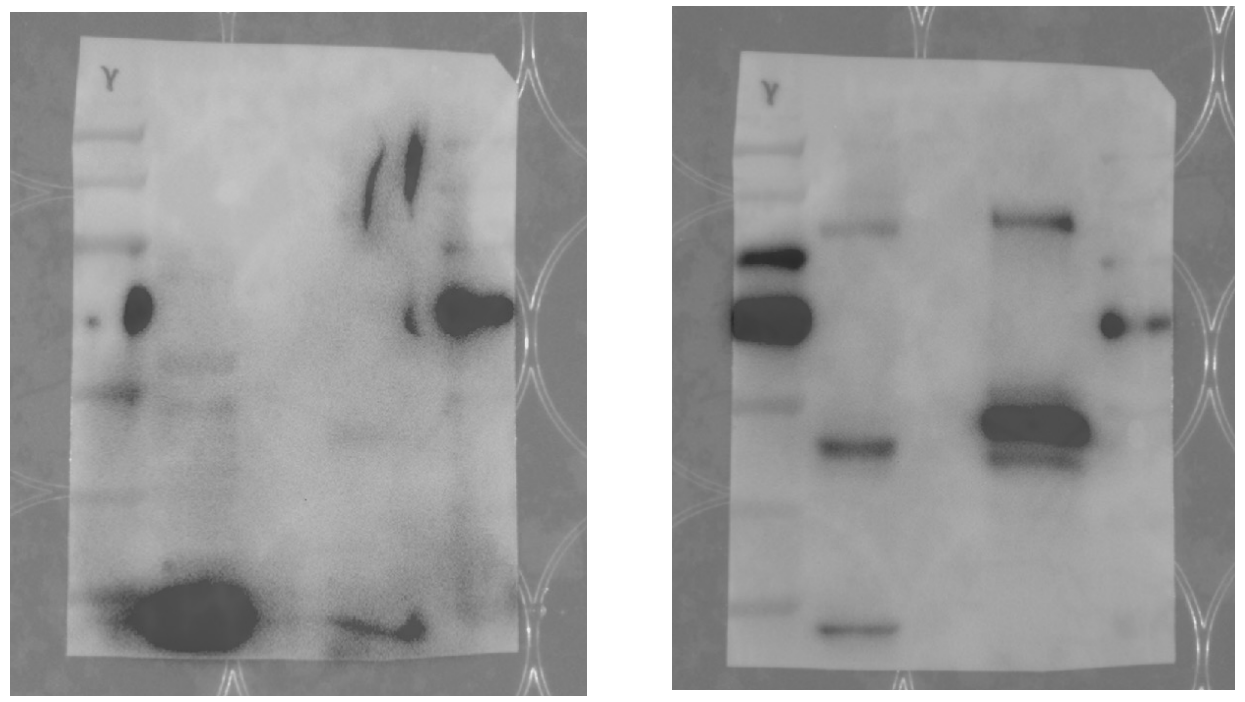

Supplementary Fig. S11D

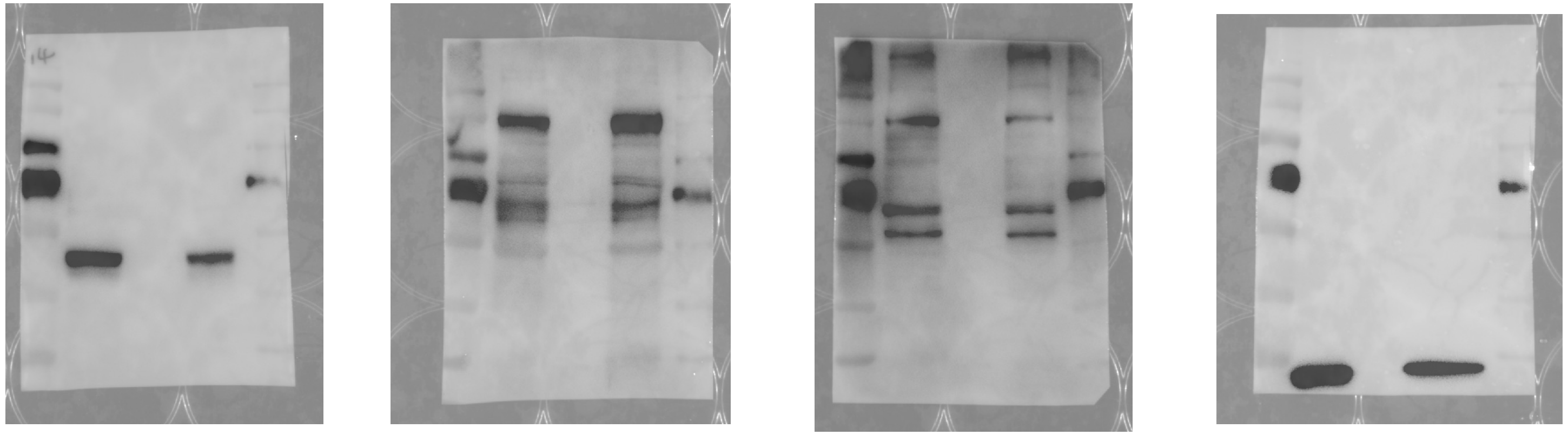

Supplement: Supplementary file 2 [file 41419_2026_9175_MOESM2_ESM.pdf]
